# The *α*/*β*3 complex of human voltage-gated sodium channel hNa_v_1.7 to study mechanistic differences in presence and absence of auxiliary subunit *β*3

**DOI:** 10.1007/s00894-025-06378-9

**Published:** 2025-05-21

**Authors:** Jordan Edilberto Ruiz-Castelan, Fernando Villa-Díaz, María Eugenia Castro, Francisco J. Melendez, Thomas Scior

**Affiliations:** 1 Laboratory of Computational Molecular Simulations, Faculty of Chemical Sciences, BUAP, C.P. 72570 Puebla, Mexico; 2 Laboratory of Basical Science, Tecnologico Nacional de Mexico, Campus Guaymas, C.P. 85480 Sonora, Mexico; 3 Center of Chemistry, Sciences Institute, BUAP, C.P. 72570 Puebla, Mexico; 4 Laboratory of Theoretical Chemistry, Faculty of Chemical Sciences, BUAP, C.P. 72570 Puebla, Mexico

**Keywords:** Sodium channel hNa_v_1.7, Molecular dynamics simulations, Molecular docking, Three-dimensional model, Protein–protein interfaces

## Abstract

**Context:**

In the context of structural interactomics, we generated a 3D model between α and β3 subunits for the hitherto unknown human voltage-gated sodium channel complex (hNa 1.7α/β3). We embedded our 3D model in a membrane lipid bilayer for molecular dynamics (MD) simulations of the sodium cation passage from the outer vestibule through the inner pore segment of our hNa 1.7 complex in presence and absence of auxiliary subunit β3 with remarkable changes close to electrophysiological study results. A complete passage could not be expected due to because the inactivated state of the underlying 3D template. A complete sodium ion passage would require an open state of the channel. The computed observations concerning side chain rearrangements for favorable cooperativity under evolutionary neighborhood conditions, favorable and unfavorable amino acid interactions, proline kink, loop, and helix displacements were all found in excellent keeping with the extant literature without any exception nor contradiction. Complex-stabilizing pairs of interacting amino acids with evolutionary neighborhood complementary were identified.

**Methods:**

The following tools were used: sequence search and alignment by FASTA and Clustal Omega; 3D model visualization and homology modeling by Vega ZZ, SPDBV, Chimera and Modeller, respectively; missing sections (loops) by Alphafold; geometry optimization prior to MD runs by GROMACS 2021.4 under the CHARMM 36 force field; local healing of bad contacts by SPDBV based on its Ramachandran plots; protein-protein docking by HDOCK 2.4; membrane insertion assisted by OPM; Berendsen V-rescaling for NVT; Parrinello-Rahman and Nose-Hoover for MPT; MD analyses by VMD and XMGRACE

**Supplementary Information:**

The online version contains supplementary material available at 10.1007/s00894-025-06378-9.

## Introduction

**Fig. 1:**
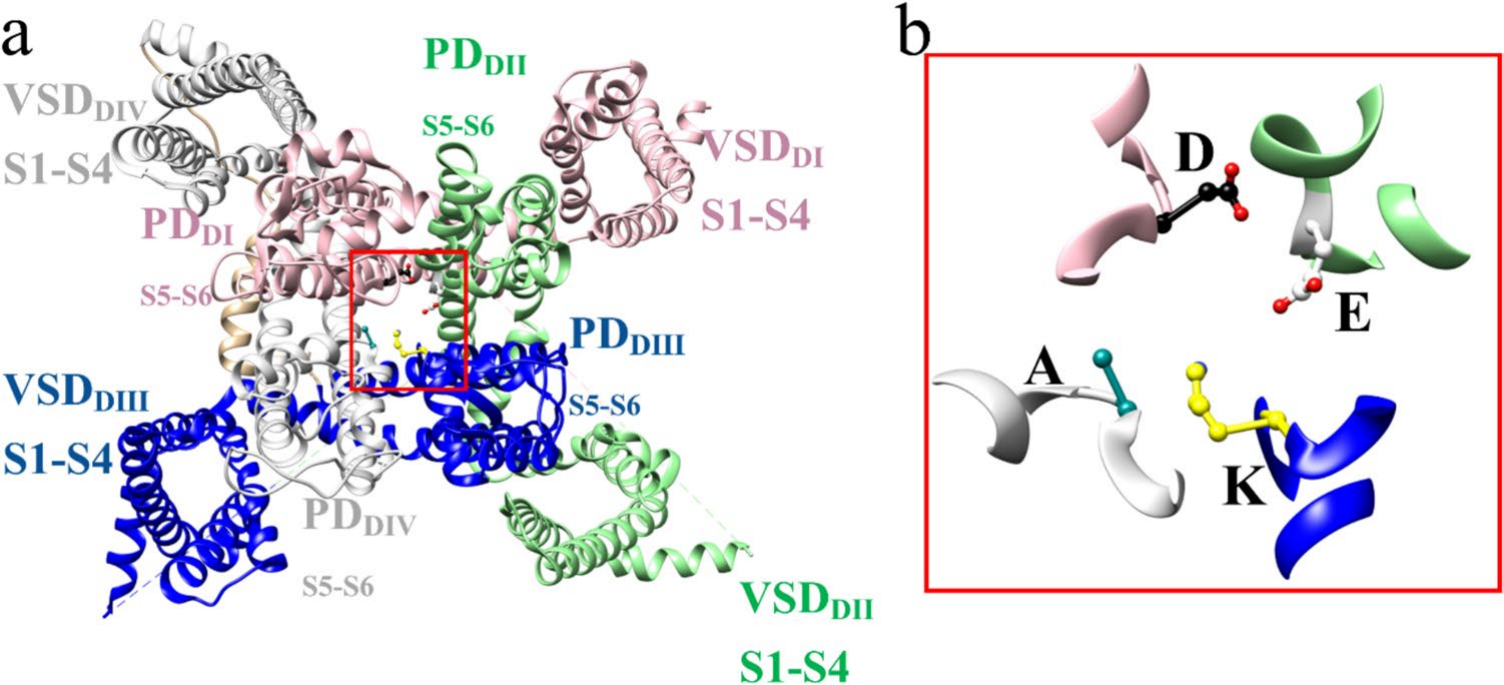
Three-dimensional topology of the sodium channel, **a** the topology shows the positions of the four voltage-sensing domains (VSDs) and the four pore domains. The color coding is pink for DI domain, light green for DII domain, blue for DIII domain, and gray for DIV domain. **b** The selectivity filter is made up of aspartic acid (black), glutamic acid (light gray), lysine (yellow), and alanine (cyan), lysine being the most crucial for selectivity

The human voltage-gated sodium channel (hNa_v_) is subdivided into nine channel subtypes by their alpha (*α*) subunit (hNa_v_ 1.1 to 1.9). In the case of our target channel hNa_v_1.7, small organic compounds have been reported which modify its gating kinetics [1]. The channel constitutes a multi-subunit complex, and its presence in the cell membrane is required for vital electrophysiological Na^+^ influx and depolarization processes [2–4]. While the central membrane-spanning *α* subunit comprises the pore, the beta (*β*) subunit behaves also as a channel activity modulator. The central unit is formed by four domains (DI–DIV) and each domain is made up of six transmembrane segments (S1-S6). The manner of distribution/folding allows the VSD voltage-sensing domains to form and be made up of the S1–S4 segments of each domain, resulting in four VSDs (VSD_DI_, VSD_DII_, VSD_DIII_ y VSD_DIV_), which are positioned around the pore domain, which is made up of the S5 and S6 segments of each domain, allowing the formation of 4 pore domains (PD; PD_DI_, PD_DII_, PD_DIII_, PD_DIV_). Within the pore domains, there are relevant amino acids that confer selectivity to the sodium ion; these four amino acids are known as the DEKA selectivity filter. This acronym is due to the fact that it is made up of aspartic acid, glutamic acid, lysine, and alanine [4]. (Fig. 1), while all *β* subunits (*β*1 to *β*4) belong to V-type Ig-like an extracellular, the transmembrane segment, and the intracellular C-terminal [2–8].

The *β*3 subunit is mainly expressed in the adrenal gland, heart, kidney, and peripheral nervous system. It is thought to be associated with certain health conditions in patients, such as epilepsy or cardiac arrhythmia [6]. Until now, no data has been published on an atomic scale for the contact zone between the *α* and *β*3 subunits of subtype hNa_v_1.7. It was hypothesized that *β*3 could bind between *α* domains DII/DIII and DIII/DIV.

Biophysical studies observed for Na_v_1.5 a possible direct modulation in the DIII-mediated deactivation process [9]. The *β*3 subunit generates changes in the inactivation of the steady state towards more positive potentials [10] and allows a recovery of the channel from the inactivated state [11]. In addition, it was observed that *β*3 changed the voltage values necessary for activation towards hyperpolarizing potentials and increases the number of channel pores which remained available in the membrane for pore opening [12], and makes Na_v_1.7 less susceptible to several blocking agents [11].

The *β* subunits are transmembrane proteins with an N-terminal extracellular domain. The latter belongs to the all-beta protein superfamily, which does not show helical secondary structures but almost entirely beta sheets (all-beta topology). Their overall fold is homologous to the V-set family of immunoglobulins (Ig), which includes cell adhesion molecules (CAM) [6]. In the extracellular domain, it presents very large handles that are called complementarity determining regions (CDR’s); these are responsible for sensing or interacting with the antigen and, in this case, with the sodium channel [13]. They regulate neuronal excitability and constitute druggable biomolecular targets [8, 14].

The PPI site between the subunits *α* and *β*1 has already been elucidated for various voltage-gated sodium channels (Nav). PDB entries for Na_v_1.2: 6 J8E [15], for Na_v_1.3: 7 W77 [16], for Na_v_1.4: 6 AGF [17], for Na_v_1.6: 8 FHD [18] and for hNa_v_1.7: 6 J8I [19]. At the beginning of this study, only PDB entries (https://www.rcsb.org/) to the year 2019 were available (see Table S1 [20]). Two PDB entries were released, one before we started (6 J8I [19]) and one mid-way (7 TJ8 [21]) with cryo-electron microscopy (cryo-EM) structures. PDB entry 6 J8I shows *α*/*β*1 complex of hNa_v_1.7, but not its *α*/*β*3 complex—whereas the other PDB entry 7 TJ8 represents an *α*/*β*3 complex, but not for the voltage-gated type 1.7 of human sodium channels (hNa_v_1.7). Until now, the complex between subunits *α* and *β*3 has not been determined experimentally for hNa_v_1.7 [22–46]. Hence, we generated it computationally to study the *α*/*β*3 PPI at an atomic scale. We compared our 3D model to both cryo-EM images as references. They complement each other: 6 J8I with a correct α but incorrect *β*, and 7 TJ8 with an incorrect α but a correct *β*3 subunit in the complex structure (see Table S1). Recently, the biochemical electrophysiological modulation of tetrodotoxin (TTX) and conotoxin as binders to Na_v_ *α*/*β* complexes was reported [47–51]. Regulation of genes which express Na_v_1.7 channels, along with *β*4, was studied in cancer-related health issues [52, 53]. The *α* subunit of human Na_v_1.7 has also been related to peripheral pain signalling toward the brain and associated with certain genetic variations of the human gene SCN9 A. Some of them could change individual pain perception in patients [54–62]. In more extreme cases, after suppression of the channel expression, a complete loss of sensitivity was observed in animal studies [63, 64]. The findings helped develop a new group of drug candidates as so-called “Na_v_1.7 inhibitors” [65–69]. Other genetic studies with subunit *β*3 found implications for impaired cardiac activities, such as atrial fibrillation [70].

Little information, such as glycosylation patterns, but no structural information has been provided for the Na_v_1.7 *α* and *β*3 complexes until 2013 [12]. Possible interaction zones between *β*3 and *α* were systematically analyzed for all known sodium channel subtypes by our group, and it was proposed that the subunit *β*3 would be in contact with domain DIII [71]. The Ig-like domain interacts with the extracellular portion of the transmembrane segment of S1 and S2 [24]. Earlier, other group members carried out mutational and computational studies on *β*1 subunit, trying to identify amino acids at the hitherto unknown *α*/*β*1 interface [72]. They described a loss of channel function by patch-clamp tests for *β*1 with a double mutant, which turned out to be a glycosylation site when the crystal structure was elucidated in 2017. Two adjacent hairpin residues, TN, were mutated to AA (dubbed as TANA), which probably hampered posttranslational trafficking to the cell surface (frog oocytes for tests), all of which led to the aforementioned loss. Recently, the binding of *β* to *α* central unit was elucidated by cryo-EM (PDB entries: 6 J8I and 7 JT8). Interestingly, the amino acids at the Na_v_1.7 and Nax *α*/*β* interfaces have remained remarkably conserved, with an 85% identity score, while other parts did not. The DIII domain of both channel types—which is the domain of *α*/*β* interaction—has an overall identity percentage of 53%.

For a better understanding of the binding of auxiliary/accessory proteins to voltage-dependent sodium channels, it is necessary to generate experimental models and theoretical structural models that explain the PPI. Identification of those PPI residues is a prerequisite to explain the molecular events of channel modulation. The functions of certain residue segments and binding sites for the *β*1 subunit on several of the sodium channel isoforms have been reported. On the other hand, how *β*2 and *β*4 subunits bind to the sodium channel through an extracellular disulfide bridge in the N-terminal domain has been described, too. Albeit, research concerning *β*3 subunit modulation on sodium channels has been reported to a much lesser extent than for *β*1. Hence, not only the PPI but also the modulation mechanism had both remained unknown [71–80]. Further evidence comes from published findings on ion channel research with other types than voltage-dependent sodium channels [81]. Despite the sheer number of structural information released until the start of this study in 2019, and beyond, the structure of Na_v_1.7 complex between *α* and *β*3 subunits has not been experimentally elucidated (Table S2). Hence, in this study, we aimed at generating the model at atomic scale and characterizing the interactions between both subunits to shed light on the computed interface between Na_v_ *α* and *β*3 subunits. The second purpose was to simulate sodium cation passage through *α* in the presence and absence of *β*3. This could be achieved only for the upper part of the hNa_v_1.7 pore segment because the model reflects the inactivated state of the underlying template.

## Methods

### Retrieving and analyzing primary sequences

FASTA search for primary sequences of the subunits and multiple sequence alignments (MSA) were carried out at EMBL-EBI with the web-based tools (https://www.ebi.ac.uk/jdispatcher/sss/fasta and https://www.ebi.ac.uk/jdispatcher/msa/clustalo) [82–85]. General bioinformatic tasks, like MSA or inspection of PDB entries, were carried out by Vega ZZ version 3.2.3 [86].

### Modelling the α subunit

The sequence of the *α* subunit of the sodium channel hNa_v_1.7 was obtained from UniProt (https://www.uniprot.org/uniprotkb/Q15858/entry). Homology modelling was based on two structures: (i) 7XVF [42] to model the N-terminal domain by homology; (ii) 6 J8I [19] to model the transmembrane portion by homology. We used AlphaFold for our incomplete hNa_v_1.7 model (https://alphafold.ebi.ac.uk/entry/Q15858) to add the missing α helices found in the intracellular segments. Moreover, the spatial coordinates of PDB release 4 JPZ were extracted [21] to model the C-terminal domain. Modeller 9.14 was installed as add-on program [87–90] to the Chimera package version 14 [91] to create the final target structure by homology modeling with the aligned UniProt target sequence [92]. The models coming from the different structures were merged under Pymol [93]. The quality of the complete model was verified using the QMEAN value. To this end, its PDB model was uploaded to the Expasy QMEAN database [https://www.expasy.org/resources/qmean] [94, 95]. High quality can be expected with values from 0.6 to 1.0 range. All QMEAN values lay in this range, too, for the transmembrane segments of N- and C-terminal domains (Fig. S2).

### Modelling the β3 subunit

The amino acid sequence of the human *β*3 subunit was retrieved from UniProt KB under the UniProt code Q9 NY72 (https://www.uniprot.org/uniprotkb/Q9NY72/entry). MSA on *β* isoforms was carried out using the sequences in Fasta format from the EMBL-EBI website (https://www.ebi.ac.uk/jdispatcher/sss/fasta) under Clustal Omega (https://www.ebi.ac.uk/jdispatcher/msa/clustalo) [82–85]. The resulting primary sequence comparison yielded a closer relatedness by homology between target *β*3 to *β*1 than *β*3 to *β*2 or *β*4 (Table 2).

Next, the 3D model of the human *β*3 subunit was created under Chimera version 14 [91]. The extracellular domain of *β*3 could be taken directly from a homo-trimeric crystal structure of *β*3 (PDB entry 4L1D [24]). To model the missing transmembrane part only, we searched known PDB entries with a *β* complex and discarded those with *β*2 or *β*4 as not suitable 3D templates (PDB entry 6 J8I [19]). The transmembrane segment, along with the intracellular C-terminal domain, had to be extracted from another crystal structure of *β*1 which had the highest homology (PDB entry 6 J8I [19]).

The extracellular (at the N-terminal segment) of *β*3 had already the correct amino acid primary sequence. In contrast, the primary sequence of the transmembrane segment had to be changed from *β*1 to *β*3 using again Modeller 9.14 [87–90] under Chimera version 14 [91]. Extracellular and transmembrane domains were fused under Pymol [93]. After merging 4L1D and 6 J8I to generate a complete 3D model of *β*3, we verified that no residues were cut out or duplicated. The homology modeling tool of version 9.14 of Modeler was used, which has been integrated in Chimera as add-on [87–91]. This way, our final 3D model was based on the complete primary protein sequence for human *β*3 from UniProt KB with UniProt code Q8 NY72 (https://www.uniprot.org/uniprotkb/Q9NY72/entry) [92]. The quality of the *β*3 subunit model was assessed as mentioned before for the alpha subunit. The resulting value range was the same (Fig. S2).

### Refining the structure of the 3D models

Prior to creating a crystal structure–like complex of the two subunits, each one had to be converted into a refined 3D model because bad contacts (clashes) existed between many side chains. Hence, each model underwent a separate geometry minimization process during 100 ps in the CHARMM36 force field version 2021 (http://mackerell.umaryland.edu/charmm_ff.shtml#gromacs) under GROMACS version 2021.4 [96–98]. Both *α* and *β*3 subunits were placed into larger molecular systems, AKA solvent boxes. Each box contained the respective subunit plus solvent molecules of TIP3P water type with the solved electrolyte NaCl at a concentration of 0.15 M. The system was exposed to a temperature of 310.15 K. Firstly, the system was subjected to NVT and NPT minimizations for 100 ps each.

After minimization, no bad contacts were observed applying Chimera’s verification tool (Menu > Tools > Structural Analysis > Find clashes/contacts) [91]. In the same way, the Psi and Phi angles were also compared using Ramachandran tables obtained from the SwissPDBViewer tool [99, 100] (select > all; Wind > Ramachandran Plot). Fig. S3 panel (a) illustrates hNa_v_1.7 subunit α before minimization, whereas panel (b) displays hNa_v_1.7 subunit α after structural minimization. In addition, Fig. S4 panel (a) shows subunit *β*3 before structural minimization. Panel (b) depicts subunit *β*3 after structural minimization. After the CHARMM36 refinement of *α* and *β*3, they both were extracted from their respective TIP3P water box. Both refined and water-free models served as input for docking simulations by HDock [101, 102] and HADDOCK 2.4 [103, 104].

### Modelling the α/β3 interface by docking

By means of molecular docking, the unknown PPI between *α* and *β*3 was computationally determined. To this end, we used HDock [101, 102] and HADDOCK software for flexible docking [103, 104]. HDOCK requires the input in PDB format of both proteins. The standard settings were applied. The output complex was retrieved from the Web page [102]. HADDOCK requires both proteins separately as input, with a limited number of up to 150 interface residues. Our model was restrained to 150 contacting residues on each side of our *α*/*β*3 model. The docking result was retrieved from the web server. Back docking was performed on the hitherto known *α*/*β*1 complex of PDB entry 6 J8I. Next, blind docking at the unknown *α*/*β*3 interface was carried out. The docking score was transformed into a confidence score to assess the probabilities of complex formation between both proteins. The docking outcome was visualized under Chimera.

### Modeling the complete system for molecular dynamics

In the next step, we added more components to the 3D model, as outlined in the following:

(i) A pre-established 1-palmitoyl-2-oleoyl-sn-glycero-3-phosphocholine (POPC) molecule was chosen as a building block for the lipid membrane on the WEB-online platform CHARMM-GUI (https://www.charmm-gui.org/) [105–107]. (ii) An extended membrane model was generated with 1298 POPC. (iii) Next, it was minimized and equilibrated during the preparation step on this Web platform. (iv) Thereafter, our *α*/*β*3 complex (without solvent box) was processed under CHARMM36 force field conditions, allowing the N-terminal and C-terminal to meet their positive (+) and negative (−) charges, respectively. Next, the *α*/*β*3 complex was placed in the middle of the membrane system with the orientation of orientations of proteins membranes (OPM) [108] and solvated with TIP3P water. (v) A salt concentration of 0.15 mM NaCl was added to reflect isotonic physiologic concentrations. To avoid the movement of the protein chains during the minimization processes, a force of 100,000 kJ/mol-nm^2^ was added to the heavy atoms. (vi) An energetic minimization of 500,000 steps was performed to allow the system to be at a total force below 1000 kJ/mol nm. The system underwent two successive thermodynamic equilibration simulations: (1) NVT (500,000 steps equal to 1 ns) under a modified Berendsen V-rescale thermostat, with the LINCS constraint algorithm at a temperature of 310.15 K and followed by another non-productive run, namely (2) NPT (1,500,000 steps equivalent to 3 ns) under a Parrinello-Rahman type barostat with a Nose–Hoover type thermostat, cubic interpolation 4 (PME-order), short-range electrostatic cutoff, and short-range van der Waals cutoff to 1.2 nm, Fourier-spacing 0.16, using the same LINCS constraint algorithm at a temperature of 310.15 K. Prior to production runs, this complete system was optimized again to avoid bad contacts and geometric distortions.

Three systems were generated to determine the stability of the system: (i) complete *α*/*β*3 subunit in water; (ii) DIII domain of the *α* subunit/*β*3 subunit in a lipid membrane; and (iii) complete *α* subunit/*β*3 subunit in a lipid membrane (Table 1). Taking a temperature variation of 323 K, generated to ensure that there are no phase changes in the lipid membrane, as Tieleman showed that due to the physiological temperature, in a simulation there can be membrane rupture or a poor recreation of the biological environment [109]. In addition, to show that there is stability in the model since it could become depolymerised in the simulation.

**Table 1:** Systems performed to assess the interaction between both subunits

System	Temperature (K)
Complete *α* subunit in water	310.15
Complete *α* subunit in water	323.00
Complete *α*/*β*3 subunit in water	310.15
Complete *α*/*β*3 subunit in water	323.00
DIII domain of the *α* subunit/*β*3 subunit in a lipid membrane	310.15
DIII domain of the *α* subunit/*β*3 subunit in a lipid membrane	323.00
Complete *α* subunit in a lipid membrane	310.15
Complete *α* subunit in a lipid membrane	323.00
Complete *α* subunit/*β*3 subunit in a lipid membrane	310.15
Complete *α* subunit/*β*3 subunit in a lipid membrane	323.00

### Running the complete system by molecular dynamics

To study the *α*/*β*3 interface, as well as the sodium ion passage, MD production runs had to be applied on a huge system after incorporation of (i) a lipid membrane model, (ii) TIP3P water, as well as (iii) NaCl ions to the target protein complex.

The prepared system was subjected to the leap-frog integrator with 50,000,000 to 1,500,000 000 steps, resulting in MD data for observation time windows between 100 and 300 ns. Finally, the production runs were carried out under GROMACS [97–99], in its 2021.4 version, in a supercomputing center at the “Laboratorio Nacional de Supercomputo del Sureste de Mexico.” The generated trajectories were visualized under the VMD program [110] and the graphs were generated with the XMGrace program [111]. To analyze the trajectories of the MD simulation, it is done using commands that are specific to Gromacs 2021.4. To calculate the root-mean-square deviation (RMSD) is used to measure the stability/changes of alpha carbons in both subunits through time [112]. This is done using the command gmx_rms. To generate the root-mean-square fluctuation (RMSF) is done using the command gmx_rmsf, and to analyze the distance of the amino acid, it is done using the script “distance.tcl” specific to VMD, which measures the distances between amino acids from the centre of mass.

### Determining the affinity of the Ig-like domain for the sodium channel

From the MD results, affinity calculations were performed with the Linux-based tool APBS (adaptive Poisson-Boltzmann solver) at https://server.poissonboltzmann.org/ to determine the interaction energy and its solvent accessible surface (SAS) (Fig. S1, Fig. S2 and Table S1). To calculate APBS, a file containing the following parameters is needed. These help us to describe the compound or residue by means of an isosurface in which it tries to find the possible contacts it makes. The file must contain: (i) the specification of the periodic boundary conditions to be able to calculate the Poisson-Boltzmann equation (pdie = 2.0); (ii) specify the number of grid points per processor (dime = 161 161 161); (iii) specify the length of the coarse grid (cglen = 261 255 293); (iv) specify the fine mesh domain lengths in a multigrid focusing calculation (fglen = 174 170 193); (v) specify that the nonlinear (full) Poisson-Boltzmann equation should be solved (npbe); (vi) specify the dielectric constant of the solute molecule (pdie = 2.0).

## Results and discussion

### Three-dimensional model generation

**Fig. 2:**
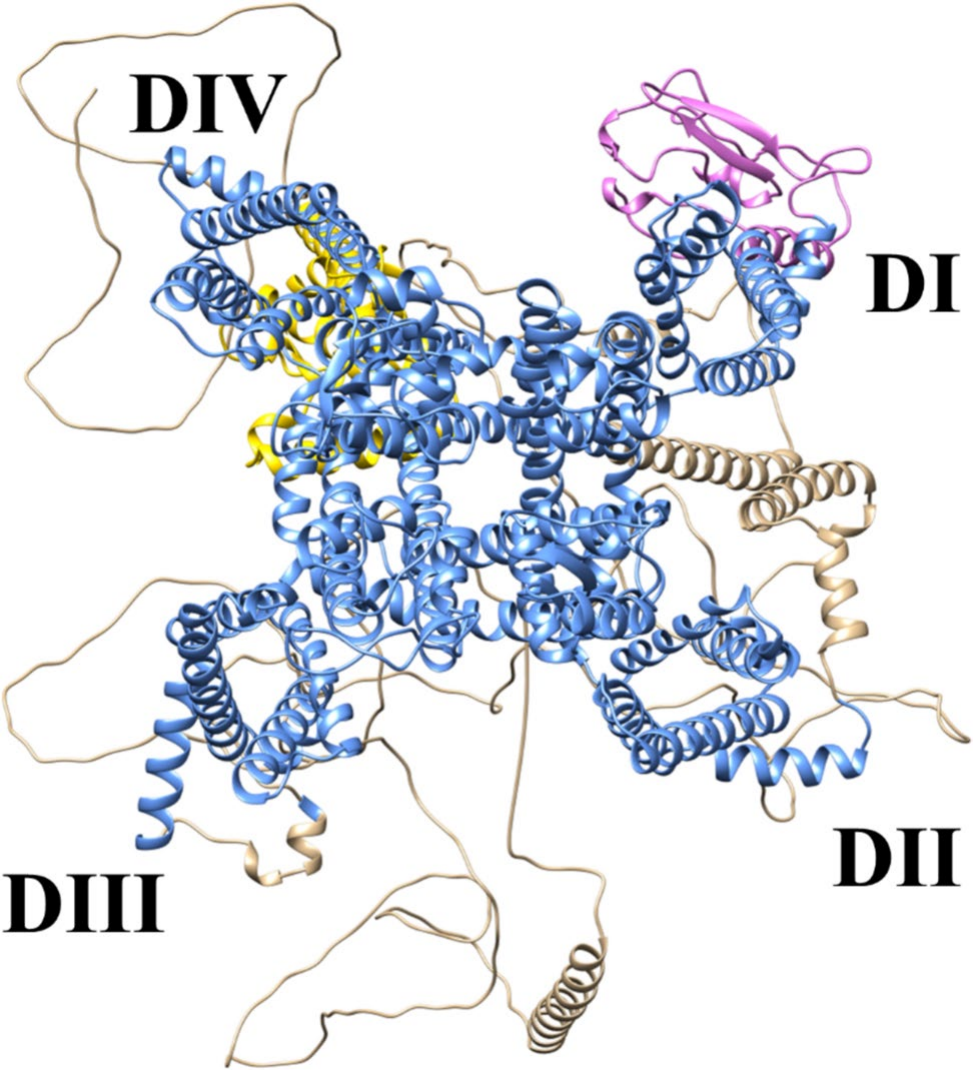
3D model of hNa_v_1.7. Color code: orchid for the N-terminal part from template 7XVF; cornflower blue for transmembrane segments from template 6 J8I; tan from Alphafold for a missing intracellular loop; gold for the C-terminal part from template 4 JPZ

The 3D structure of the extracellular domain of human *β*3 was extracted from PDB entry 4LID (Table S1). To generate the complete 3D model of the *β*3 subunit, we fused the structural information for the transmembrane and intracellular domains from 6 J8I, which constitutes *β*1 of hNa_v_1.7 (Table S2). To generate the complete α subunit model of hNa_v_1.7, three homologous 3D templates were used: (i) 7XVF for the N-terminal part; (ii) 6 J8I for the transmembrane main section with the extracellular loops. Precalculated models of intracellular loops were retrieved from the Web-based server Alphafold (https://alphafold.ebi.ac.uk/) [113]; (iii) 4 JPZ for the missing C-terminal domain in the incomplete 3D template 6 J8I (Fig. 2). Like 7XVF and 6 J8I, 4 JPZ also constitutes a closely related 3D template with 70% residue identity. For more details concerning the 3D model generation, see the Supplementary Materials (SM) with the corresponding Fig. S3 to Fig. S7.

### Subunit arrangement analysis

**Fig. 3:**
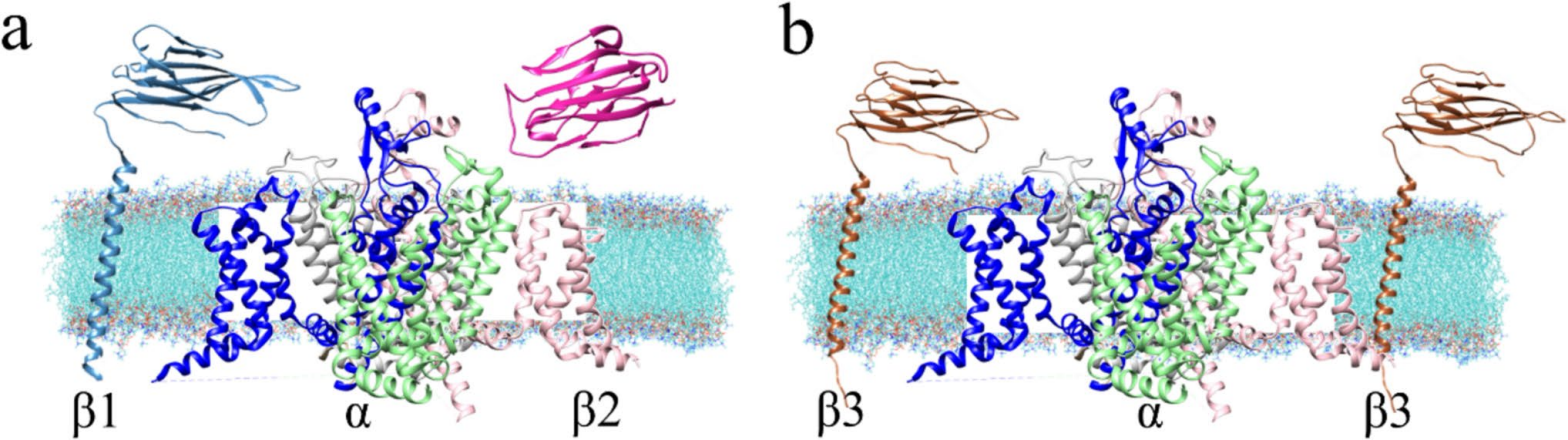
Topology of subunits on hNa_v_1.7. **a** Structurally known *β*1/*α*/*β*2 complex. **b** Two possible arrangements for *β*3 for hitherto unknown *α*/*β*3 complex. The diagonal line symbolizes the PPI. Color code: *β*1 subunit cyan, *β*2 magenta; sodium channel: DI domain pink; DII light green; DIII blue; DIV light gray; and *β*3 subunit sienna, and a lipid membrane in blue with red dots

Since the VSDs of the channel form a heterotetrameric monocadenar subunit (*α*), it is not far-fetched to assume the segments S1 to S4 of each could be those in contact with *β*3. Structural information had been deposited at PDB prior to the start of this work with 5XSY (*α*/*β*1 for eel, 2017) and 6 J8I (*β*1/α/*β*2, for human, 2019). This implies that associations of *β*2, *β*3, or *β*4 were not available during this study. While the location of *β*1 and *β*2 was elucidated by cryo-EM, the location of *β*3 could theoretically replace *β*1 or *β*2 (Fig. 3). Also in good keeping, the hitherto known literature has attested that DII and DIII or DIV were the most probably *β*3 contact partners [10, 12].

### Docking analysis at the protein–protein interface

**Fig. 4:**
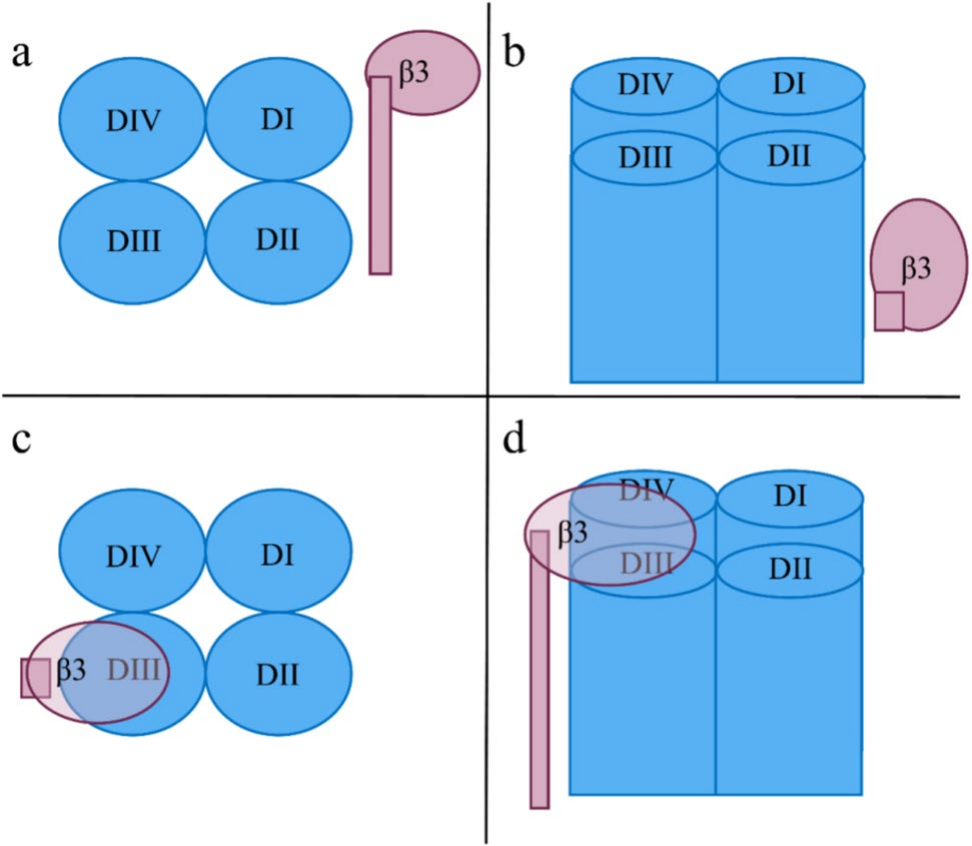
Random start position in panels **a** and **b** and the final docked pose in panels **c** and **d**. Panels **a** and **c** are top-down views, while panels **b** and **d** are lateral views. Color code: pink *β*3 and blue *α* subunits with four domains D1 through DIV

Since the available PDB structures did not represent our specific *α*/*β*3 complex but rather complexes with other types of *β* subunits, we carried out docking simulations with random start positions by HDock [101, 102] and HADDOCK2.4 [103, 104] (Fig. 4). For more details concerning the docking results between the *α* and *β*3 subunits, see SM with the corresponding Fig. S8 to Fig. S14.

The homology of the three types of *β* subunits was also assessed at this stage of work (Table 2).

**Table 2:** Identity score percentage for *β*1, *β*2, and *β*3

	**Eel *β*1**	**Human *β*1**	**Human *β*2**	**Human *β*3**
	**[PDB code 5XSY]**	**[PDB code 6 J8I]**	**[PDB code 6 J8I]**	**[PDB code 7 TJ8]**
Eel *β*1	100	46	20	42
h*β*1	46	100	21	50
h*β*2	20	21	100	23
h*β*3	42	50	23	100

**Fig. 5:**
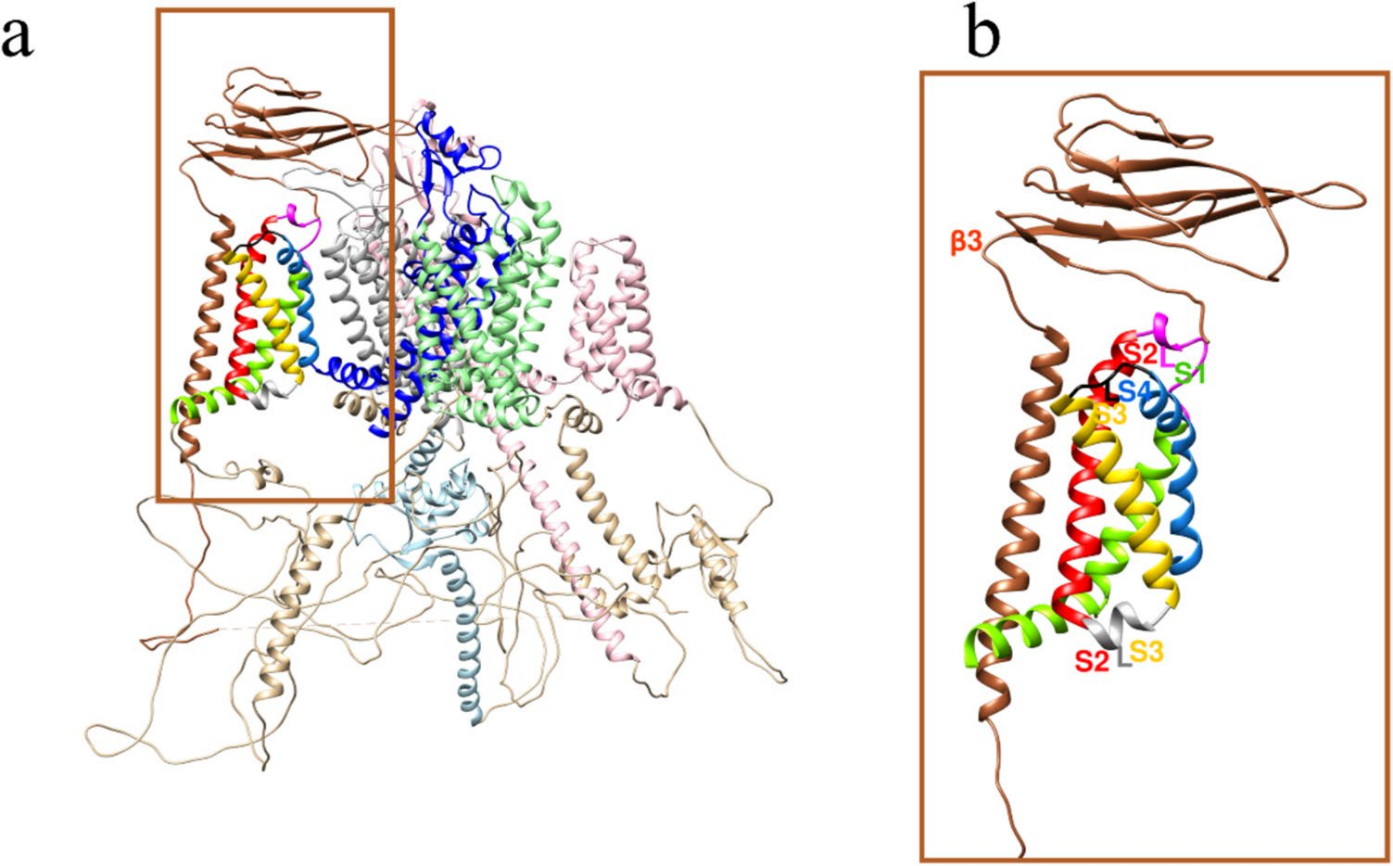
3D model of the docked *α*/*β*3 interface. The *α*/*β*3 interface is located on the DIII domain of the subunit *α*. Only the mechanistically relevant DIII segments are displayed. Two extracellular loops at the *α*/*β*3 interface are labelled with their transmembrane segments (S1-L-S2 and S3-L-S4). The extracellular Ig-like domain of *β*3 contacts both loops. Between both loops lies another loop facing the intracellular side between S2 and S3 (gray ribbon). The C-terminal part of *β*3 also orients toward the inside (C-ter). Color code for protein backbone ribbons: *β*3 model in sienna; S1, S2, S3, and S4 of *α* subunit in chartreuse, red, yellow, and cornflower blue colors; DI, DII, DIII, and DIV in pink, light green, blue, and light gray, respectively. Colors for two loops at the PPI: the first between S1 and S2 in magenta; and the second between S3 and S4 in green

To study the *α*/*β*3 interface, we needed a 3D model of the *α*/*β*3 complex. We chose random start positions for the *β*3 as a sort of “ligand” for the α subunit “receptor” with its huge molecular size. The docking results were inspected, and the docked poses were found only slightly different from the reference structures *α*/*β*1 (PDB code 6 J8I) or *α*/*β*2 (PDB code 6 J8I) [19]. Figure 5 shows the PPI model with two extracellular loops between transmembrane segments S1 and S2, as well as S3 and S4 on domain DIII of the *α* subunit in complex with *β*3. As a proof of concept, the *β*1 ligand in the *α*/*β*1 complex was successfully self-docked to its position in the reference complex (PDB entry 6 J8I [19]). Almost at the end of this study, with the advent of an experimentally determined *α*/*β*3 complex, the computed model with domain DIII as the interaction site could be validated (PDB entry 7 TJ8 [21]). For details about the computed results and determination of the protein–protein complex by MD, see Fig. S15 through Fig. S40 in SM.

### Stability analysis for molecular dynamics model

**Fig. 6:**
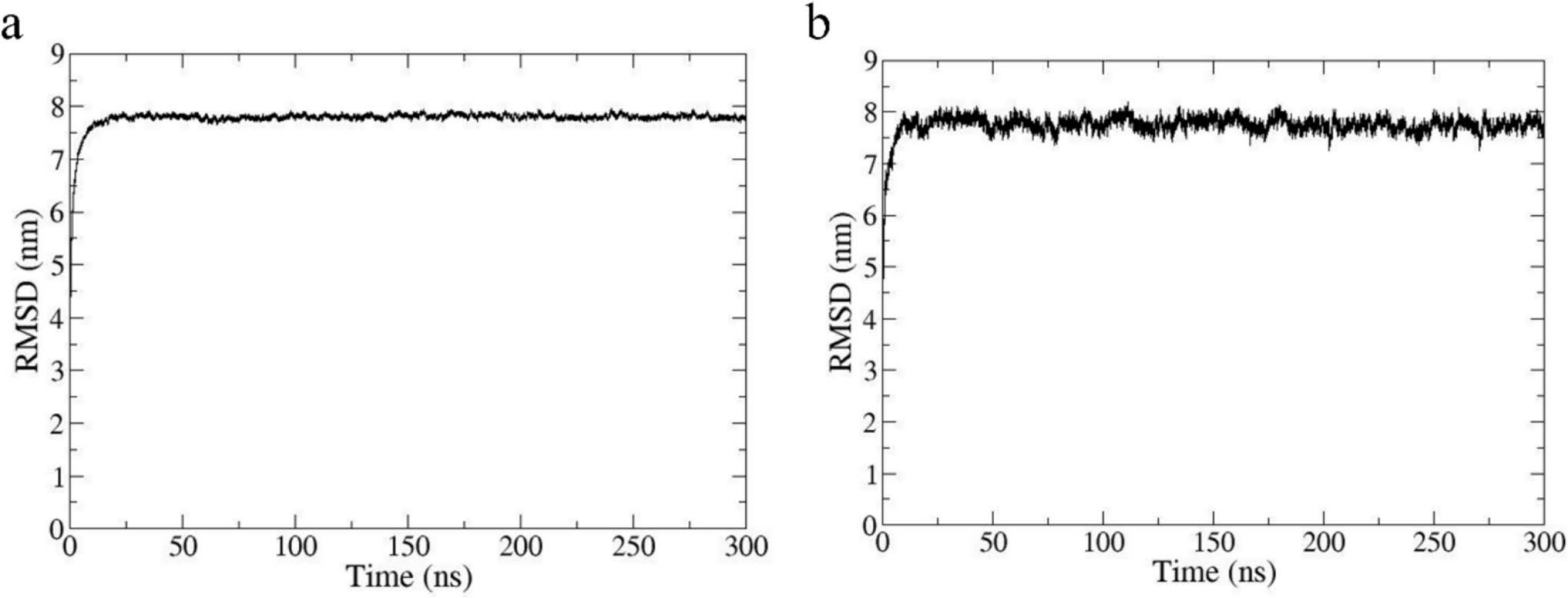
RMSD plots of the *α*-subunit and *β*3-subunit of the hNa_v_1.7 sodium channel over 300 ns. **a** RMSD plot of the *α*-subunit is presented, and **b** RMSD plot of the *β*3-subunit is presented

The hitherto unknown PPI between both subunits (*α*/*β*3) of hNa_v_1.7 were studied at an atomic scale by MD [114]. The 3D model, which the simulation was generated, is made up of (i) *α* subunit; (ii) *β*3 subunit; (iii) lipid membrane of POPC; (iv) NaCl 0.15 M; and (v) water tip3p at 310.15 K. This complex model stability under MD conditions between both subunits is remarkable since there was no 3D template available at the time of the study for 3D model generation. Root mean square deviation analysis of atom distances (RMSD) showed that the model system remained stable during the production run of 300 ns and beyond (Fig. 6). RMSD averages the distance measurements of initial atom positions to their respective positions at a given moment in time during MD production runs. Values ranging from 3 or 4 to 6 or 7 could reflect larger displacements in space, e.g., domain shifts, structural deformations, or overall model instabilities [115]. Global RMSD studies include geometrical changes in irrelevant molecule parts, but RMSD for selected atoms focuses on spatial movements of interest [116].

This finding also reflected that our system was successfully prepared by minimization with NVT followed by NPT prior to MD production runs. Of note, the rising RMSD values in the first 10 ns did not reflect structural instability, but rather indicated rearrangement in the extra- and intracellular loops (a in Fig. 6). On the other hand, the *β*3 subunit behaves similarly due to its intracellular loop which underwent rearrangements during the first 10 ns. Thereupon, minor fluctuations in the RMSD ceiling line can be detected, which stem from the highly mobile complementarity determining regions CDR1, CDR2, and CDR3 of the extracellular domain on *β*3 (b in Fig. 6). Typically, they possess antigenic functions in Ig proteins [117]. The fact that the amino acids on both proteins have really “seen” (contacted/interacted with) each other during eons of evolutionary time led to a successful combination of docking and MD simulations with the computed identification of the *α*/*β*3 interface. Otherwise, missing neighborhood effects would have led to molecular models with more repulsion than attraction forces.

**Fig. 7:**
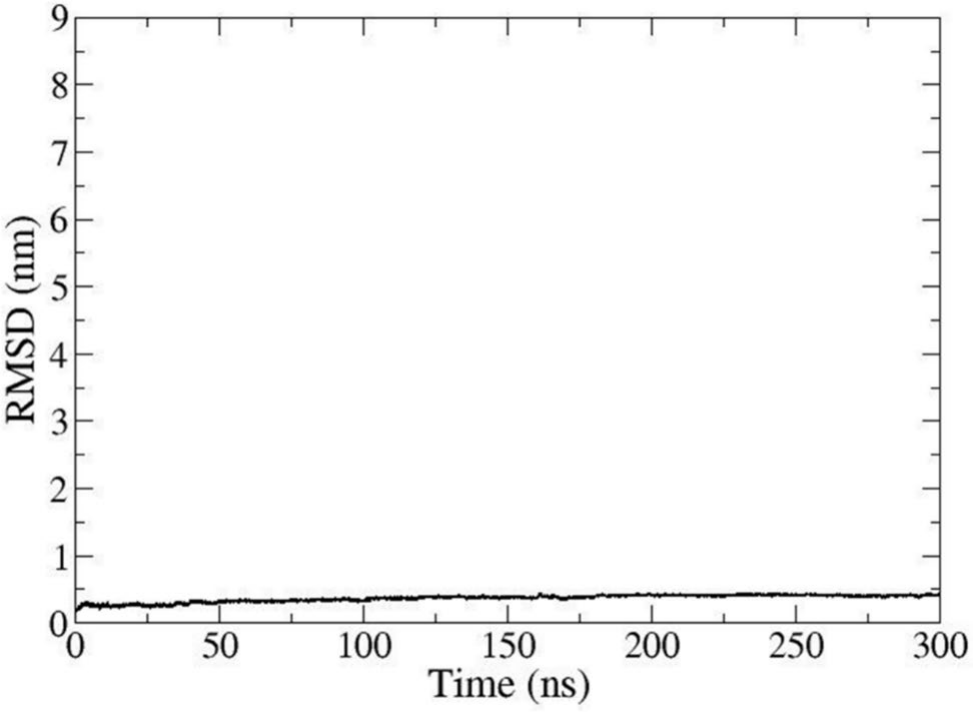
RMSD plot of VSD_DIII_. The MD run lasted 300 ns for a larger observation time window. The RMSD values on the graph were registered for atoms of S4 in movement

To explore the overall stability of the 3D model with hNa_v_1.7, the MD observation time was extended to 300 ns. In Fig. 7, the data was filtered to focus on the domain DIII. The RMSD values remained constant in a range of approximately 0.1 nm, which represents a typical value for overall protein stability. RMSD values were based on the alpha-carbon atoms of the DIII amino acids. The protein stability was deduced—despite the steady increment of the absolute RMSD values—because the values oscillated around 0.1 to 0.2 nm (RMSD fluctuation).

Even the longest MD test run with 300 ns proved that the interface was stable without indications of unfolding the tertiary or secondary structures. During eons of evolution, the interaction between both has been adapted and optimized. Such neighboring effects concern the residues in the voltage sensing domain (VSD) of DIII (VSD_DIII_) [116, 117]. Switching from structural to functional aspects of MD, this phenomenon of side chain proximities between corresponding counter amino acids on both subunits reflects a channel modulation process engaging with DIII of the *α* subunit of hNa_v_1.7. The complex remains stable since the RMSD for DIII oscillates very little during 300 ns of the large observation time window (Fig. 6 and Fig. 7).

**Fig. 8:**
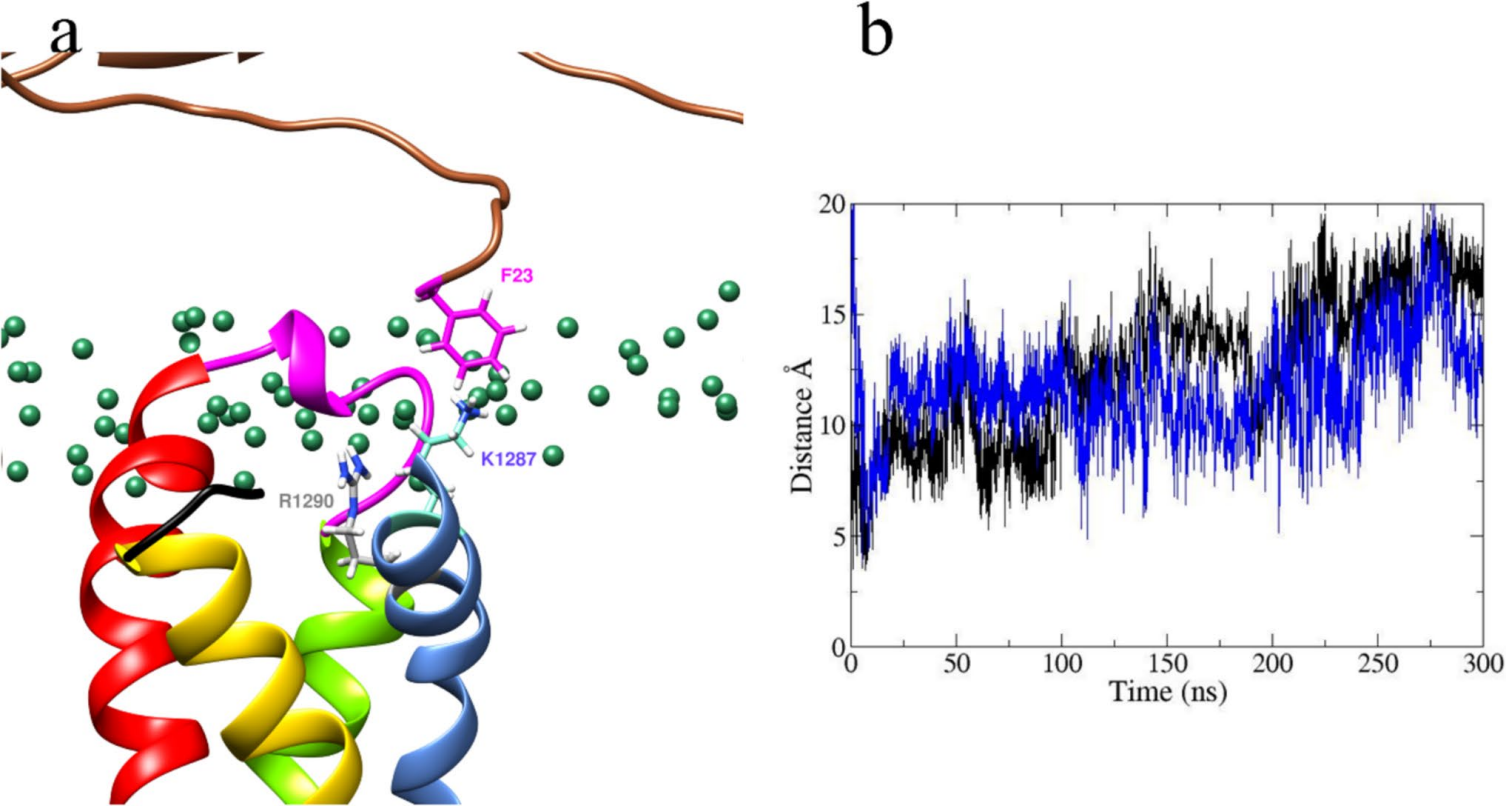
Representation of the interaction between F23–K1287 and F23–R1290. Panel **a** Interaction between F23 (fuchsia) to K1287 (aquamarine blue) and F23 (fuchsia) to R1290 (gray) which is reflected by the steeply descending slope and the upwards movement at 10 ns. Figure was made with Chimera. Panel. Color code for protein backbone ribbons: *β*3 model in sienna; S1, S2, S3, and S4 of *α* subunit in chartreuse, red, yellow, and cornflower blue colors; the green sphere is the phosphate group of lipid membrane. **b** Plot of the distance in the formation of a cation-π interaction between F23–K1287 (black) and F23–R1290 (blue) during the 300 ns

We observed S4 mobility in the VSD_DIII_ of the DIII domain. Figure 8 shows details for the participation of two residue pairs K1287 (on *α*) with F23 (on *β*3) as well as R1290 (on α) with F23 (on *β*3). At the beginning of the simulation, if the interaction between F23 and K1287 is carried out by cation-π, and with arginine, they are only long-range electrostatics, since, as Infield (2021) and Gallivan (1999) mention, the interaction must be well oriented and with a distance of 6 Å [118, 119]. Both cationic residues belong to the VSD. Table 3 lists the positive charges from all the side chains of Arg or Lys for DI through DIV.

**Table 3:** Listing of the positive charges of arginine and lysine side chains at the VSD [93]

Domain	Total positive charge	Top-down order of Arg and Lys residues with id numbers
DI	4	R214, R217, R220, K223
DII	5	R835, R838, R841, K844, K847
DIII	6	K1287, R1290, R1293, R1296, R1299, R1303
DIV	5	R1610, R1613, R1616, R1619, R1622

**Fig. 9:**
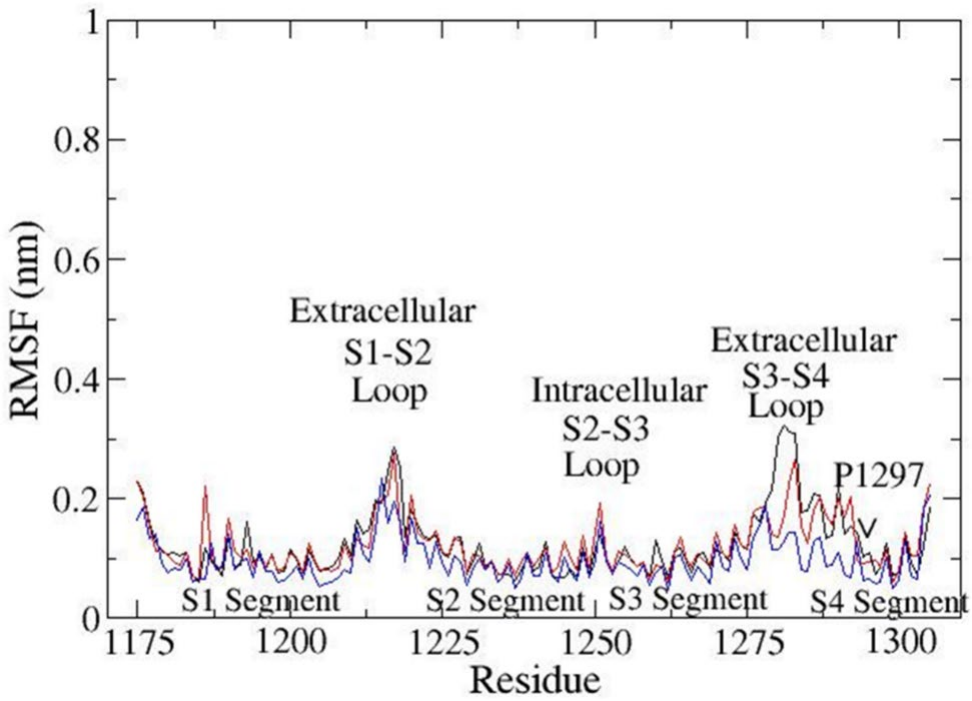
RMSF plot for VSD_DIII_ of the α subunit over the 300 ns of registered productive run time. The high mobility of the amino acid residues shows the elevation on the RMS: the first peak is formed with amino acids from S1 to S2 extracellular loop. The latter embraced amino acids 1212 to 1224. The second peak was composed of amino acids from the S2 to S3 intracellular loop with residues 1244 to 1257. The residues 1277 to 1284 belong to the extracellular loop between S3 and S4, while the first amino acids of the S4 segment were identified as residues 1287 to 1297. The lines show the movement of the structures through the 300 ns; black line = 0–100 ns, red line = 100–200 ns, blue line = 200–300 ns

**Fig. 10:**
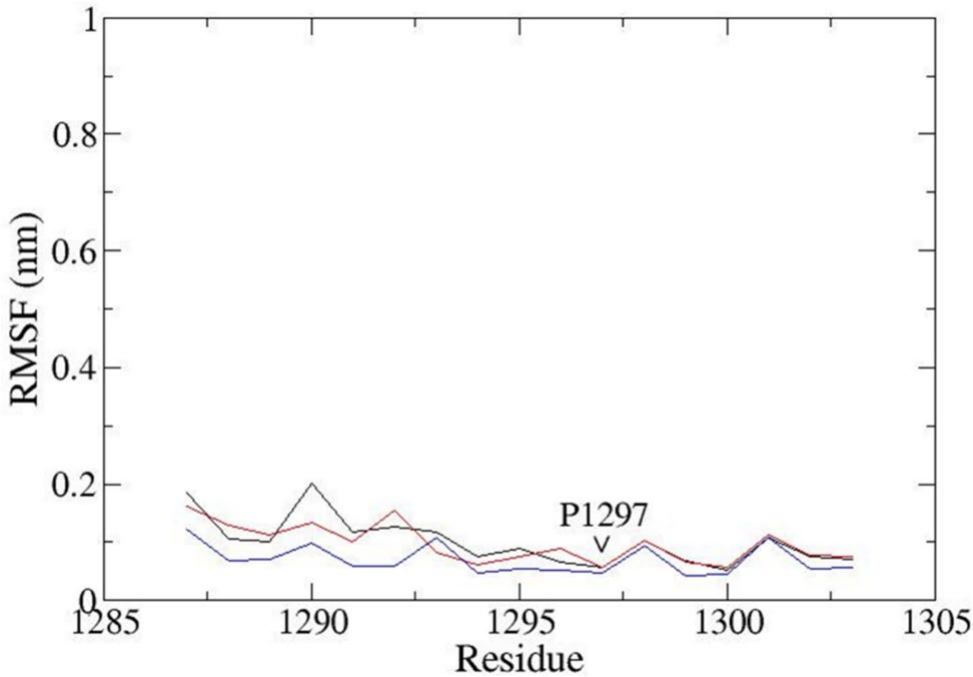
Representation of the RMSF of the S4 segment of the α subunit. The proline amino acid residue 1297 (P1297) generates a restriction process, making the proline 1297 have greater freedom of movement than before. The lines show the movement of the structures through the 300 ns; black line = 0–100 ns, red line = 100–200 ns. Blue line = 200–300 ns

As a proof of concept for not only the neighboring effects but also to which degree our MD reflects natural behavior can be extracted from root mean square fluctuation (RMSF) charts (Fig. 9). Proline P1297 is situated in a helical segment and produces a so-called proline kink (see P1297 between sequence position 1250 and 1300 on the *X*-axis in Fig. 9 and Fig. 10 [120–127]). The total 300 ns observation time was divided by 3. The resulting three 100 ns observation windows in superposition (Fig. 9) revealed an almost periodical repetition of particle mobility, all of which also demonstrates the structural stability—a prerequisite to preserve biological function here of the *α* subunit. The movement of the other VSD of the channel has a high movement because they do not have a protein that helps them slow down their movement, compared to VSD_DIII_.

Details about proline P1297 movement can be drawn from the MD playback (Fig. 10). On the RMSF chart (Fig. 10) a more mobile helical region can be identified to the left of P1297, while the residues to the right of P1297 are located in a stiffer helical wheel. Of utmost essence to the mechanistic interpretation of MD, here lies in the fact that this finding nicely explains for VSD_DIII_ how the adjacent arginine residues (R1290 and R1293) move upwards into the extracellular space. And with it, an essential interaction will take place with subunit *β*3 and in direct consequence, the channel activation [126, 127].

The S4 segment undergoes a classic “cork-pulling” movement [119]. Proline P1297 of DIII generates a so-called proline kink in the *α*-helix [119–127] For this reason, The S4 segment consists of two parts: (i) flexible extracellular and (ii) more rigid. It lends larger backbone mobility for most of the extracellular part of S4, whereas, for the inward helical part in the direction of the C terminus, this residue creates a stiffer structure (see also Fig. 9).

**Fig. 11:**
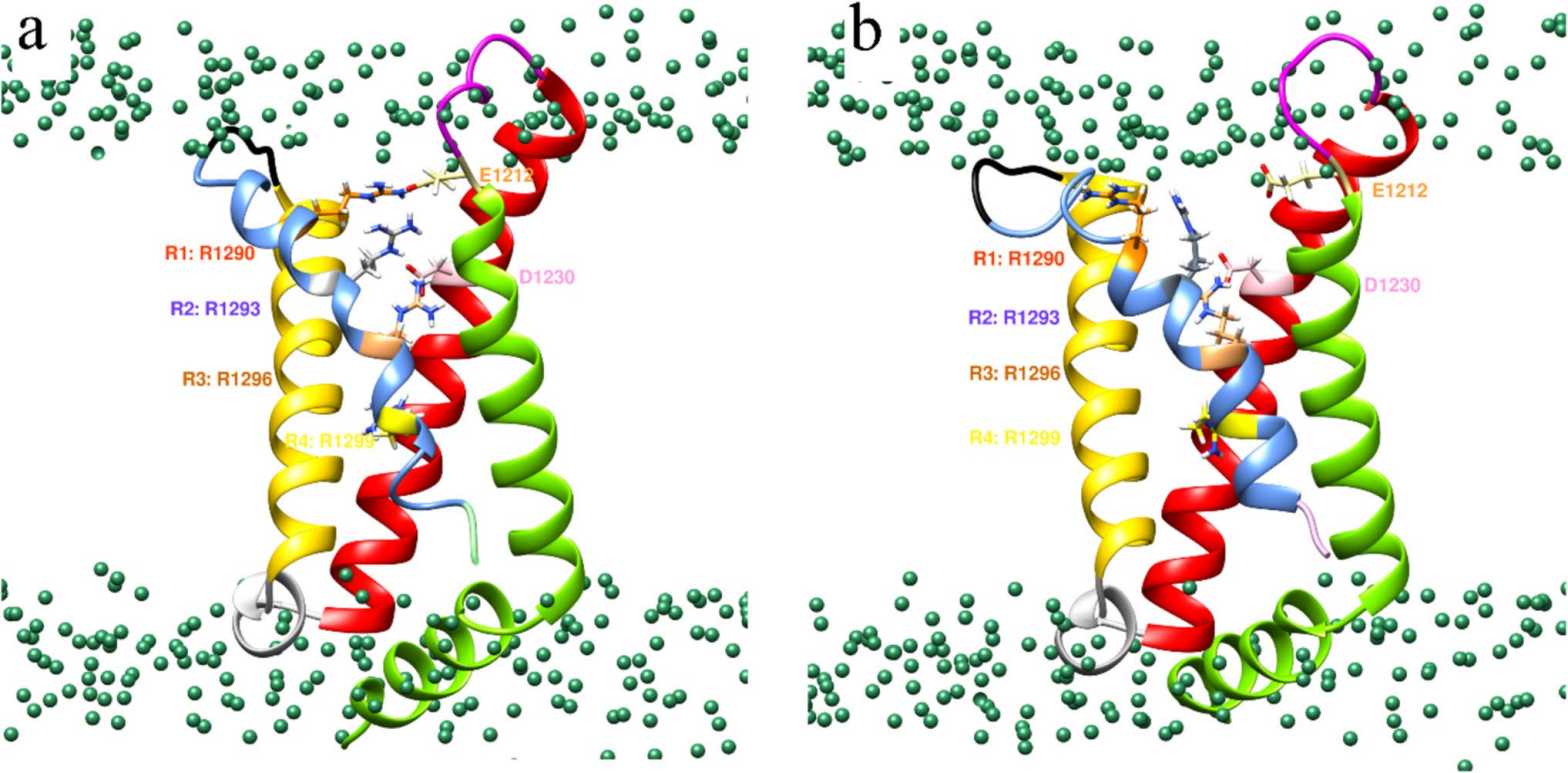
Structural representations of the conformation change of the S4 of the sodium channel, viewed from inside the sodium channel. Panels **a** and **b** are two models taken from the initial and final time windows (10 ns and 80 ns, respectively). S4 is shown in silver. Panel **a** Inactivated state: R1290 (orange) is oriented towards E1212 (tan) and forms electrostatic interactions; R1293 (gray) is placed below R1290 and interacts with E1212 (tan). They interact with D1230 and R1299 (gold) in the direction of segment S2. Panel **b** Activated state: R1290 (orange) has moved in 70 ns towards the extracellular part. E1212 (tan) interacts with R1293 (gray) as well as R1296 (sandy brown) in the same side chain conformation as R1293. Color code for protein backbone ribbons: *β*3 model in sienna; S1, S2, S3, and S4 of *α* subunit in chartreuse, red, yellow, and cornflower blue colors; the green sphere is the phosphate group of the lipid membrane

The side chain movements of mechanistically pivotal residues were analyzed over time to verify if our MD study correctly reproduces the extant literature (Fig. 11). The book chapter about “The Voltage Sensor Module in Sodium Channels” by James R. Groome and the publication by Sula and coworkers describe the changes that the segment S4 undergoes during channel passage of sodium cations, as well as the different channel geometries. Certain structures show an inactivated channel, while others represent channel geometries that lead to pore opening prior to activation [126–128]. Initially, the side chain of one of them (R1290, orange) was oriented towards E1212 (tan, according to panel (a) of Fig. 11) and R1290 is interacting face to F23 of *β*3 subunit; this allows the distance to increase across time. E1212 and R1290, at times it spontaneously formed and opened salt bridges as they quickly increased the separation distance, causing us to go from a strong electrostatic interaction to a weak one over 50 ns. In a timely coordinated or “synchronized” movement, the other arginine (R1293, with blue color in Fig. 11) reoriented, too. It is located deeper inside the channel, right below R1290. Intriguingly, E1212 was “sandwiched” between both (R1290 and R1293). This close contact does not last. Apparently, it is not a stable constellation in space since it does not obey the electroneutrality with anionic residues (*E*) squeezed in between two cationic residues (*R*). The positive total charge excess (+ 1) assists the outward bending on the extracellular end of the helical segment with E1212. This positive charge excess is a well-recognized electrophysiological feature of voltage-gated sodium channels at all four S4 segments—albeit each of them carries a distinct number of arginine and lysine residues. In the context of channel function, this fact also hints at the common characteristics for vertebrate animals, all of which have a heterotetrameric topology based on one primary sequence chain. Whereas the upper part exposed to the channel surface shows the aforementioned mechanistic feature, the architecture of the inner part remains unchanged over time for a twofold reason: (i) it is deeply buried into the membrane bilayer, which holds a firm grip on the transmembrane channel segment; (ii) an adjacent third arginine (R1296) to the two others (R1290, R1293) stays constantly in close contact with D1230. During the entire observation time, this salt bridge was never disrupted. It plays a key role in stabilizing the secondary and tertiary structural elements of S4 on DIII only. The other three domains (DI, DII, and DIV) bear 2, 3, or 5 arginine or lysine residues forming a variable number of non-permanent salt bridges to expose positively charged side chains on the channel surface in a similar mechanistic way as described here for DIII [129]. The pattern is assumed to be recurrent, although the observation time of 100 ns was extended to 300 ns, which was still too short a time frame to observe any periodicity (Fig. S27 through Fig. S40 in SM).

**Fig. 12:**
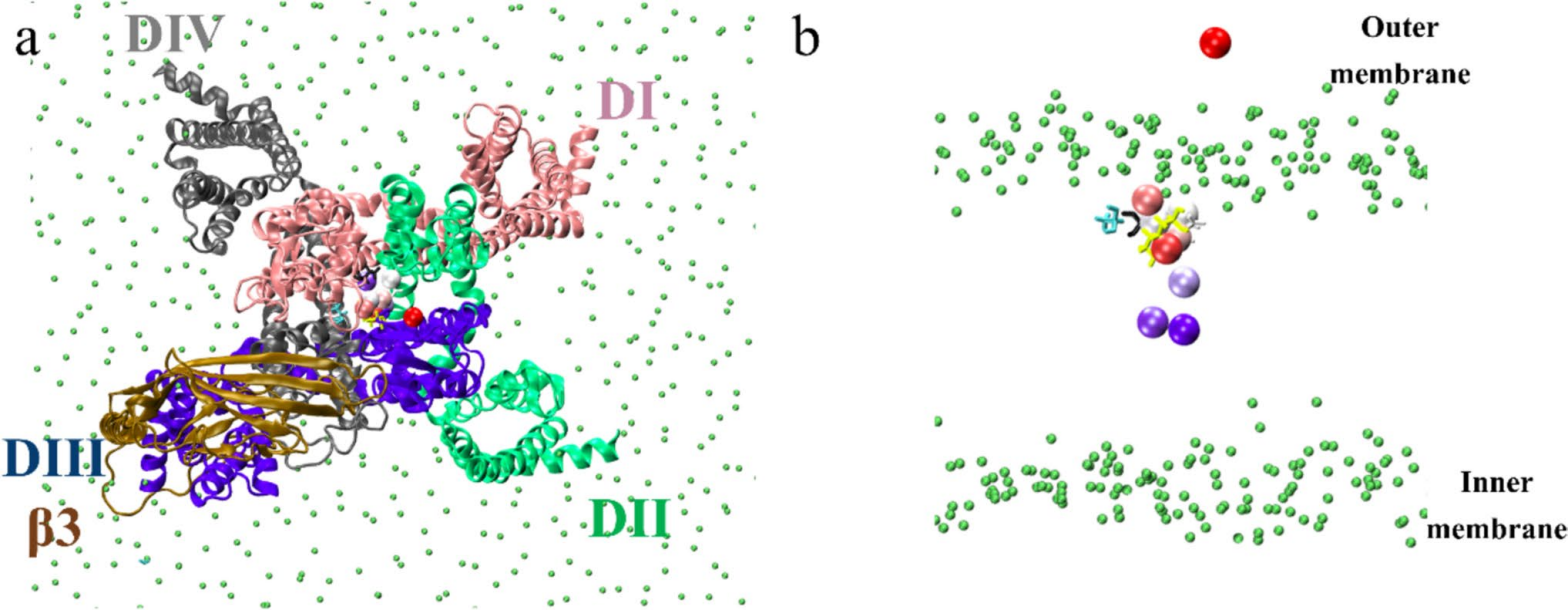
Display of the Na^+^ trajectory for hNa_v_1.7. **a** Seven sodium locations in the pore are displayed in the *α*/*β*3 complex; the movement of de Na shows the movement and approach to the selectivity filter. **b** Shows only the movement of Na + to the selectivity filter and the position when the sodium passes through across the DEKA ring; the colors of the sodium position through the dynamics range from color red at 0 ns to the passing the selectivity filter, which are light blue to dark blue. The uppermost right location is the starting point at 30 Å distance to the DEKA ring or “bottle neck” (stick model colors: D black, E gaey, K yellow, and A cyan, the green spheres are the phosphate group of POPC membrane). Of note, a deeper permeation of the sodium particle was not possible due to limited supercomputing resources at hand

**Fig. 13:**
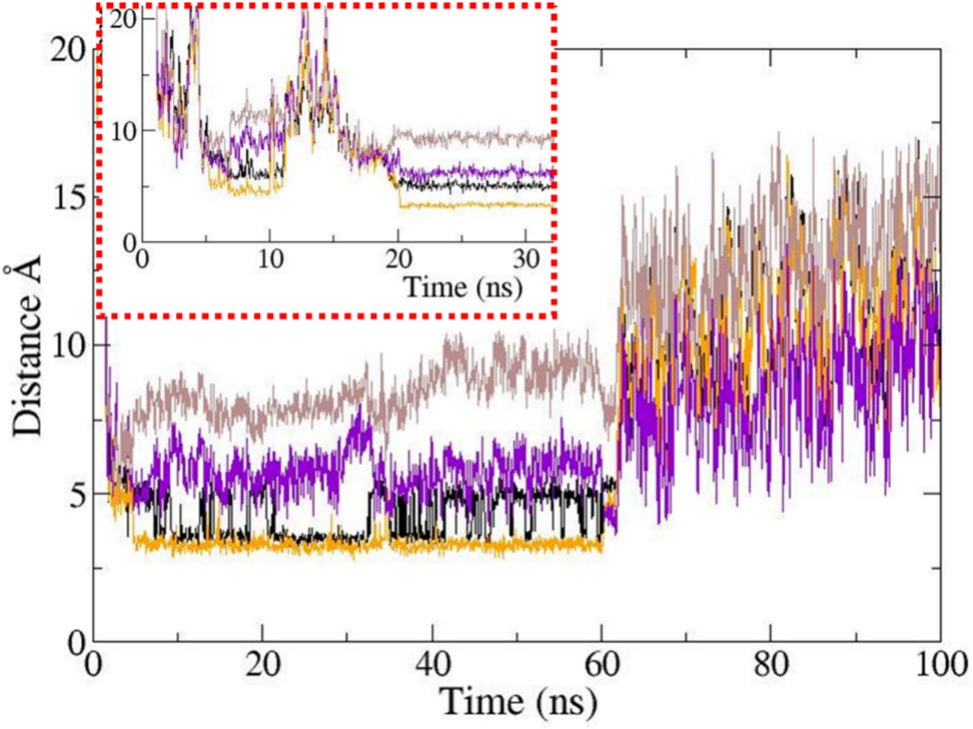
Distance plot between a passing sodium cation and the four residues of the DEKA-selectivity filter of the *α*-subunit from hNa_v_1.7. The figure (inlay) shows the Na^+^ behavior, red lines in the absence (respectively absence) of *β*3, and the bigger chart is in the presence of *β*3 subunit. The observation time on the *x*-axis for the incoming sodium ion was 100 ns; the starting distance on the *y*-axis above the outer pore vestibule was 30 Å, and finally, the Na ion passes through because the amino acid lysine of the DIII domain accommodates, showing an increase in distance at 30 ns. A Line colors: black Na + to D; orange Na^+^ to E; purple Na^+^ to K; brown Na^+^ to A

Upon comparison of the MD simulation with *β*3 against the other one without *β*3 under the same conditions, the electrophysiological role of β3 became evident (Fig. 12 and Fig. 13). In 2013, Leadermann et al. showed that the *β*3 presence allows an increase in current density [12] that translates into an increase in the rate at which sodium ions can approach the DEKA selectivity filter. In the MD simulation, it can be observed how the Na ion approaches the selectivity filter more quickly because the *β*3 subunit interacts with extracellular loops, preventing the path towards the selectivity filter from being disturbed. In this way, it is known that the sodium ion takes about 30 ns to interact with the lysine of DEKA and to finally be able to pass inside the channel. On the contrary, when the channel is simulated without the *β*3 subunit, the Na ion takes about 20 ns to approach the selectivity filter, but the sodium ion can still pass after 50 ns. Once the sodium cation reached DEKA and enters the inner vestibule, both models show highly similar behavior measured as RMSD of Na + against the four residues of DEKA. In Fig. 11, this finding is graphically documented: on the *x*-axis is the timeline in nanoseconds. During the first 5 ns, the Na + particle enters the channel pore from the extracellular space. The distance values for the four DEKA residues swiftly decreased while the sodium cation approached them. The chart integrates its movements with respect to each of them as reference locations by super-positioning four lines displayed in four different colors. Two totally different electrophysiological behaviors can occur: one in the presence and the other in the absence of *β*3. To visualize these differences, an inlay was created for Na + ions. The seemingly erratic movement was reflected by the fluctuating RMSD values on the *y*-axis (cf. peaks and valleys in the inlay which is contoured by red intermittent lines). In stark contrast, when *β*3 was present in the channel complex model, the incoming sodium cation moved on to the DEKA “bottle neck” without deviation in a rather straight line. The shorter path also saved time, i.e., the sodium passage was faster. This observation is in good keeping with what has been found in electrophysiological studies (patch or voltage clamp experiments) [12]. After passing the DEKA filter step, the cation reached the inner vestibule (Fig. 12). The respective RMSD values on both models were very similar. It is safe to assert that at that stage of the sodium passage there is no significant influence anymore concerning the presence or absence of *β*3. This channel segment is too deeply buried in the membrane. Conversely, our observations by MD simulations document that *β*3 has an impact upon sodium income only at the channel entrance. For details about the computed results and determination of protein–protein complex by MD studies, Fig. S15 through Fig. S40 in SM.

### Neighborhood-related interactions

MD and docking results were analyzed to identify pairs of *α*/*β*3 interacting amino acids on the extracellular PPI such as K1670/E53, E1672/R51, R1218/E28, H1721/P24, and K1287/F23 (Fig. S12 through Fig. S40 in SM). To be precise, the following three *β*3 residues help modulate the function of the *α* subunit: E53 (*β*3) with K1670 (*α*), E52 (*β*3) with K1670 (*α*) as well as R51 (*β*3) with E1672 (*α*). They stay interactive over the entire observation time, which allows modulation with respect to the EDDE filter (aka EEDD). Their distances range between 4 and 10 Å. In addition, the *α*/*β*3 pair K1220/E32 allows for the positioning of the Ig-like segment on the VSDs of the hNa_v_1.7 sodium channel.

Other *α*/*β*3 pairs do not interact permanently but are in close range and help stabilize the contact zone close to the membrane: K1176/E188; H1191/E176; Y1228/E159; F1194/W172; F1197/W172 (Fig. S11 through Fig. S37 in SM). For instance, a hydrogen bond spans temporarily between the pair Y1228/E159, but at any moment it is lost due to the movement of the membrane chains because the side chain of Y1228 is displaced and reoriented towards the lipid membrane. Hydrophobic attraction could also be observed with F1194/W172 or F1197/W172. With water moieties at a distance, their side chains form a water exclusion (hydrophobic) pocket of variable size according to the overall side chain and backbone movements. Intriguingly, F1197 and W172 rotate to enter stabilizing contact with the membrane lipids. Both cases are only a “pars pro toto” view of the vast concert of hydrogen bond networking for the entire complex, just to name a hydrogen bond residue pair H1191/E176 or a salt bridge between K1176 and E188 in the intracellular segment. For more details, see Fig. S12 through Fig. S40 in SM.

### Assumptions and model limitations

In more general terms, our entire simulation study is in excellent keeping with gathered experimental knowledge concerning the complex topology between *α* subunit *β*3, the exact location of the *α*/*β*3 interface at atomic scale, and the spatial rearrangements of interacting loops and pore segments on the subunits, along with sodium particle mobility from the outside to the inner vestibule (Fig. 12). Yet, the model was not amenable to reproducing the complete Na^+^ passage from the outside to the inside because the model is in the inactivated state and so there cannot be a sodium ion passage, which would require the open state of the channel. The use of the channel in the inactivated state allows us to observe structural changes on the *α* subunit by *β*3 subunit during the inactivated state. To determine the protein/protein stability, we carried out MD studies without membrane but with boxed water for 100 ns. In the same way, another MD run with a 5-degree tilt in the *β*3 subunit was performed. Here, the *α*/*β*3 contact was entirely lost, which represents a significant change in cooperativity between them. In other words, the Ig-like extracellular domain of *β*3 moves, and its new position lies closer to the membrane after 35 ns. A search was carried out for several docking programs, so when using it, and not having the expertise to work with it, a result came up that was very similar to that of HDOCK and HADDOCK, but when performing the dynamics, the extracellular domain binds to the membrane and stays there.

## Discussion

With the advent of PDB entry 7 TJ8 in 2022, it was possible to validate our predicted *α*/*β*3 interface because, for the first time, an *α*/*β*3 complex was elucidated by cryo-EM techniques [20]. Its chain labeled A constitutes a subunit *α*; yet, it does not constitute our target hNa_v_1.7 but a hitherto unknown class of voltage-independent sodium channel, called NaX or Na_x_. Its chain B, however, presents the entire subunit *β*3 of our target voltage-gated sodium channel with its extracellular and transmembrane domains.

In 2019, the PDB entry 6 J8I was retrieved as a 3D template at an atomic scale, although it was still not a perfect match because it is a complex with *β*1 and not all identical with *β*3, under study here, namely the human voltage-gated sodium channel hNa_v_1.7 with its PPI between subunit *α* and *β*3. So, we continued to finish our PPI model until 2024.

**Fig. 14:**
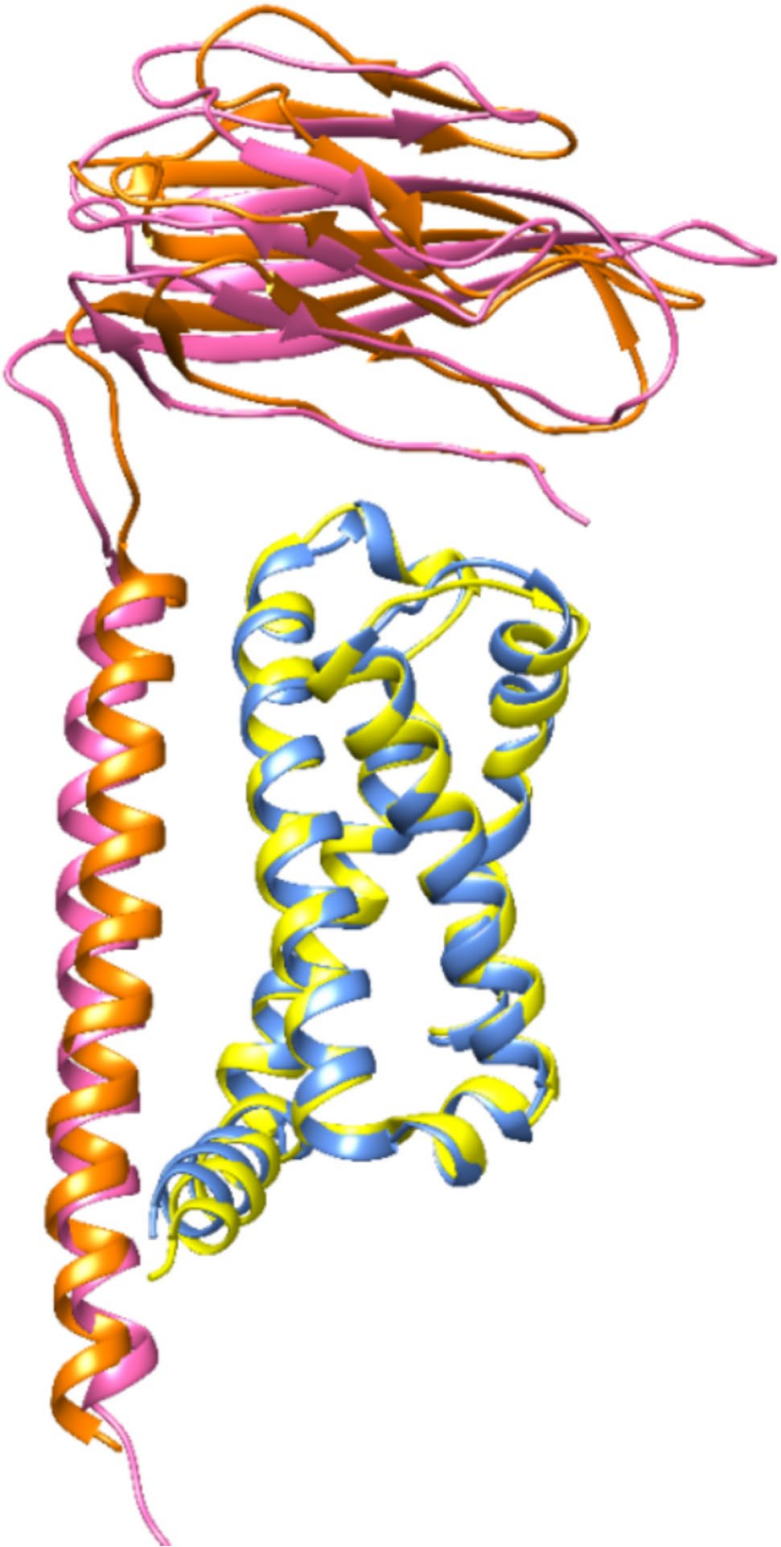
Experimentally observed structure (*α*/*β*3 of hNa_x_) and predicted *α*/*β*3 model in superposition. Color code for protein backbone ribbons: blue *α* model; pink *β*3 model; *α* of 7 TJ8 red orange and *β*3 of 7 TJ8 gold. The *α*/*β*3 interface is located at the DIII domain

**Fig. 15:**
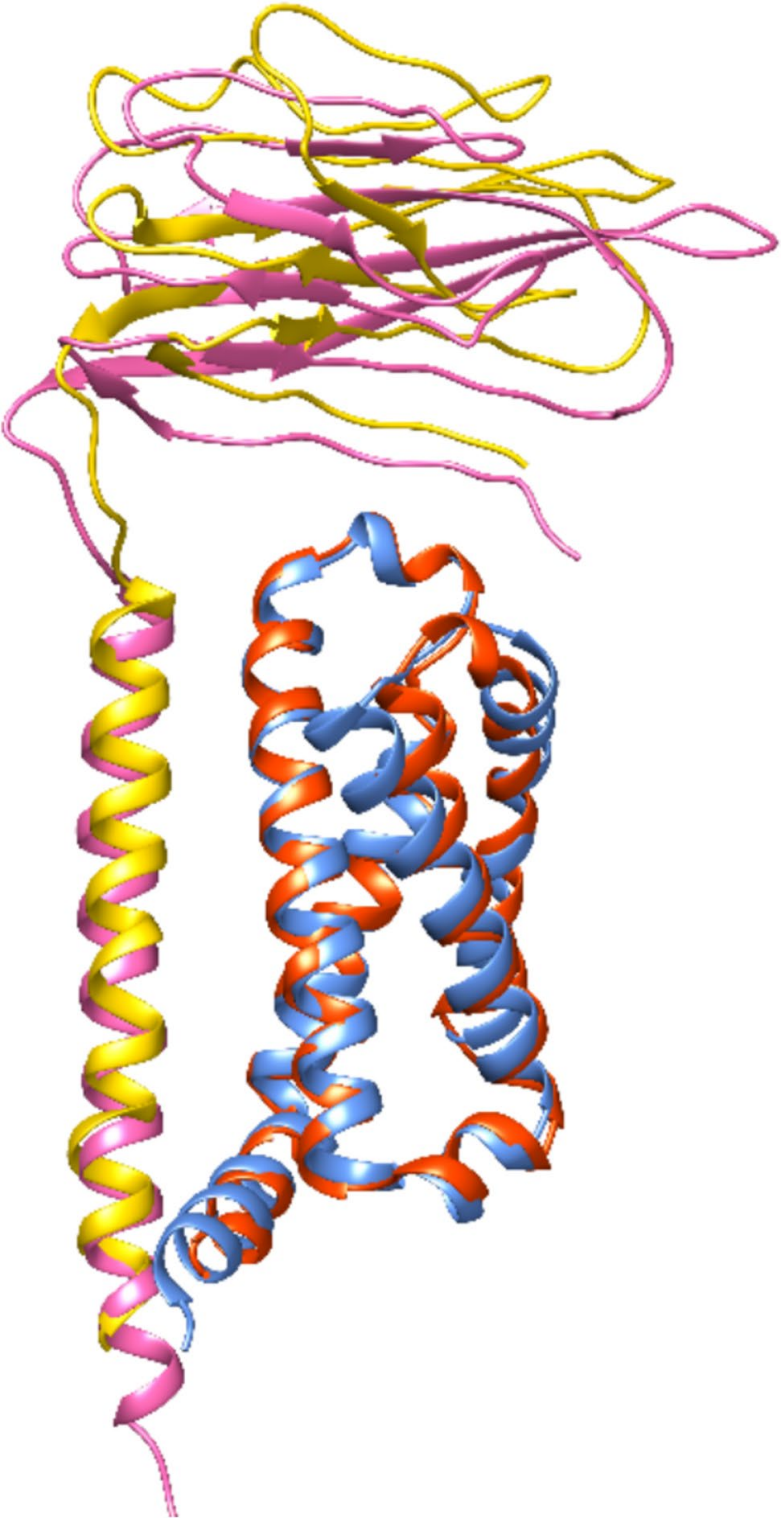
Experimentally observed structure and predicted model in superposition. The 3D alignment reveals that model *β*3 and *β*1 from PDB show large geometrical differences due to their different primary sequences. The *α* subunit of the predicted 3D model, however, fits much better than the *β*3 to the cryo-EM *α*/*β*1 complex of hNa_v_1.7 (PDB entry 6 J8I) because both *α* subunits are the same. Actually, the cryo-EM *α* subunit was extracted to create the model *α* subunit. Color code: light blue *α* model; pink *β*3 model; *α* or *β*1 of PDB entry 6 J8I = yellow or orange colors, respectively. The *α*/*β*3 interface is located at the DIII domain

As a direct result, the reference confirmed our computed *α*/*β*3 complex with the same orientation and PPI between *β*3 and DIII of subunit *α* (Fig. 14 and Fig. 15). In a final step, the structural data was compared between the reference and the target (Table 4).

**Table 4:** The RMSD of the cryo-EM structure and our three-dimensional binding site model between subunit *α* and *β*3

Reference structure	Computed model	Comparison basis	RMSD [Å]
Crystal structure 7 TJ8 with subunits *α* and *β*3	hNa_v_1.7 with the entire *α*/*β*3 complex	All 1078 pairs of atoms	3
Homology modeling	hNa_v_1.7 with a part of the *α*/*β*3 complex	Subset of 692 pairs of atoms focusing on the PPI	1
Homology modeling	hNa_v_1.7 with only DIII of the α/*β*3 complex	All 122 pairs of atoms	2
Homology modeling	hNa_v_1.7 with only DIII of the *α*/*β*3 complex	Subset of 107 pairs of atoms focusing on the PPI	1

The *β*3 subunit interacts with the S4 segment of the VSD_DIII_. The non-covalent binding in the transmembrane zone was mostly hydrophobic in nature, while those forces that stabilized the binding site of the *β*3 subunit with respect to the *α* subunit were a concert of electrostatic attractions and hydrogen bond networks.

Intriguingly, Glass and collaborators ascribed a PPI stabilizing effect to residue E176 in 2020 (Fig. S22 and S23) [25]. This residue is toward the inner side of the membrane. At the binding site, the interaction is mainly due to hydrogen bonds in addition to electrostatic attractions, as described by Vascon et al. also in 2020 [128]; this interaction prevents the movement of the inner transmembrane helix.

Also, we observed S4 mobility in the VSD_DIII_ domain with the participation of two residue pairs K1287 (on *α*) with F23 (on *β*3) as well as R1290 (on *α*) with F23 (on *β*3). These interactions show that the S4 segment has two parts: (i) a flexible one after P1297 and ii) a rigid one before P1297. This S4 mobility reflects the observation by Sokolov et al. in 2018 that the *β*3 subunit allows faster recovery from the inactivated state [11]. The interaction between K1287 α/F23, R1290 *α*/F23, and with the CDRs K1670 at *α*/E52 at β3; K1670 at *α*/E53 at *β*3; E1672 at α/R51 at *β*3; and W1354 at *α*/E129 at *β*3 describes at the molecular level the finding of the modulation process concerning sodium channel activation, as reported by Laedermann et al. in 2013 [12]. They observed an increase in current density in addition to a shift of the activation rate toward more hyperpolarized potentials by approximately 3.7 mV [12] and a shift of the average inactivation velocity (V_1/2_ inactivation) toward more depolarized potentials by approximately 1.5 mV [12].

In the context of protein evolution, neighboring amino acids at the PPI recognize each other and lend binding specificity [117]. Thanks to the CDR segments of β3 the presence of the *β*3 subunit has a favourable effect on the function of the α subunit with the central pore of the sodium channel. Hence, it is not far-fetched to assume that the stronger the affinity of the CDR segments to the α subunit at the PPI, the greater the electrophysiological effect for the channel.

Our study lends insight about membrane protein/membrane protein interactions, and our findings contribute to the interatomic research, of which we cite two seminal works spanning more than two decades of advances in PPI science [130, 131]. Drug development efforts have been undertaken in the field of human voltage-gated sodium channels, for instance, with channel blockers for skeletal muscle isoform Na_v_1.4 [132]. Details of recent channel drug research can be found in a seminal review [133].

## Conclusion

Since the human Na_v_1.7 complex between *α* and β3 subunits has not been described experimentally at an atomic scale, its 3D model was generated by homology based on three PDB templates. The resulting crystal structure-like *α*/*β*3 interface was studied in detail by MD.

As a direct finding, we observed that *β*3 subunit interacted with protein segments that modulate the voltage-dependent sodium channel. The three most relevant interactions were documented: (i) overall domain cooperativity at the *α*/*β*3 interface: in the transmembrane area, the interactions that are presented provide stability to the interaction between both proteins. (ii) Charge interaction with VSD in domain DIII: K1287 on *α*/F23 of *β*3 and R1290 on *α*/F23 of *β*3 constitute two pairs of favorable interactions between a variable segment of the Ig-like *β*3 subunit and residues belonging to the S4 segment on *α* subunit. This mechanistic insight explains how this segment moves to allow channel modulation. As a direct consequence, the channel function is modified concerning the kinetics (timing) of the channel opening and closing. (iii) Interactions with complementarity determining regions (CDRs): the Ig-like segment interacts through CDR1 salt bridges with EEDD filter amino acids, K1670 on *α*/E52 of *β*3; K1670 in a E53 and E52 of *β*3; E1672 in *α*/R51 of *β*3 while the interaction through CDR3 is through the formation of a hydrogen bond between E129 and W1354, which also belongs to the EEDD filter at the entrance of the pore. As a last finding, due to this interaction between both subunits, the sodium cation passage through the channel pore from the extracellular entrance to the inner vestibule was simulated in the presence or absence of auxiliary protein *β*3. The computed differences were in perfect keeping with published electrophysiological data.

In addition, the sodium cation passage through the channel pore from the extracellular entrance to the inner vestibule was simulated in the presence or absence of auxiliary protein *β*3. The computed differences were in perfect keeping with published electrophysiological data.

Our study contributes to the emerging field of molecular computational science to study PPI by MD. To complement our study in the inactivated state, future studies could simulate sodium ion passage in the open state of the channel.

## Supplementary Information

Below is the link to the electronic supplementary material.

Supplementary file1 (DOCX 15926 KB)

## Data Availability

No datasets were generated or analysed during the current study.
