# Supplementary material for: The α/β3 complex of human voltage-gated sodium channel hNav1.7 to study mechanistic differences in presence and absence of auxiliary subunit β3
Source: J Mol Model. 2025 May 21;31(6):168. doi: 10.1007/s00894-025-06378-9 (PMC12095431; doi:10.1007/s00894-025-06378-9)
Supplement: Supplementary file 1 — Supplementary file1 (DOCX 15926 KB) [file 894_2025_6378_MOESM1_ESM.docx]

**Supplementary material**

**The α/β3 complex of human voltage-gated sodium channel hNav1.7 to study mechanistic differences in presence and absence of auxiliary subunit β3**

**Jordan Edilberto Ruiz-Castelan,^1^ Fernando Villa-Díaz,^2^ María Eugenia Castro,^3^ Francisco J. Melendez,^*4^ and Thomas Scior,*^1^**

^1^ Laboratory of Computational Molecular Simulations, Faculty of Chemical Sciences, BUAP, C.P. 72570 Puebla, Mexico

^2^ Laboratory of Basical Science, Tecnologico Nacional de Mexico, Campus Guaymas, C.P. 85480 Sonora, Mexico

^3^ Center of Chemistry, Sciences Institute, BUAP, C.P. 72570, Puebla, Mexico

^4^ Laboratory of Theoretical Chemistry, Faculty of Chemical Sciences, BUAP, C.P. 72570 Puebla, Mexico

***** Correspondence: thomas.scior@correo.buap.mx; francisco.melendez@correo.buap.mx

Received: date; Accepted: date; Published: date

**Contents**

| Part 1: Chemometric data at the interface | 2 |
| --- | --- |
| Part 2: Molecular modelling of 3D models | 7 |
| Part 3: Molecular Docking between α /β3 subunits | 16 |
| Part 4. Molecular Dynamics between α /β3 subunits | 20 |
| Part 5. Molecular Dynamics between domain DIII analysis | 26 |

**Part 1. Chemometric data at the interface**

The zones in which the Ig-like domain of the β3 subunit is placed with the α subunit of hNa_v_1.7 changes with respect to time and in order to know its stability, the APBS calculation was performed to know the affinity in kJ/mol (Fig. S1 and S2) and through the use of PDBePISA (www.ebi.ac.uk/msd-srv/prot_int/cgi-bin/piserver) allows the observation of the sites that present interaction.


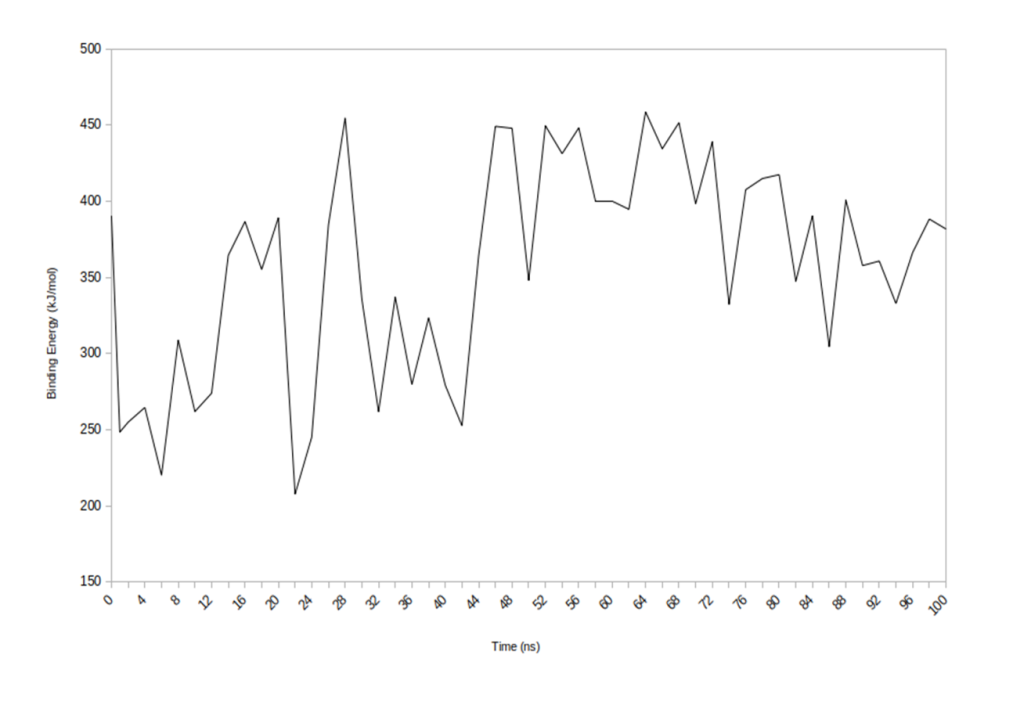


**Fig. S1** The binding energy of the Ig-like domain as it interacts with the extracellular region of the α-subunit of hNa_v_1.7. This fluctuates over nanoseconds and is most stable between 20–44 ns, reflecting the time at which it generates changes in the α-subunit.


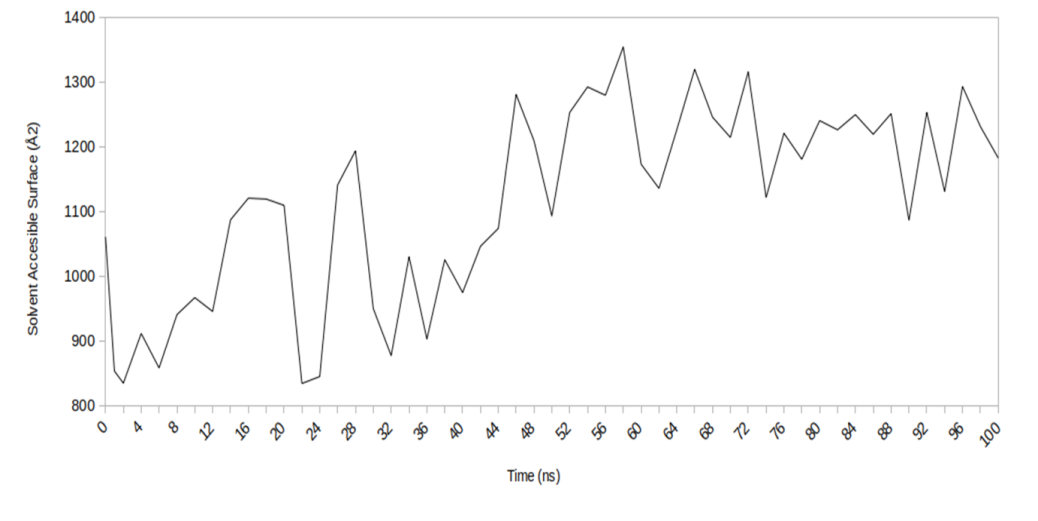


**Fig. S2** The solvent accessible surface (SAS) allows us to know how the Ig-like domain interacts with the water in the system. At the beginning and when generating the effects on the α-subunit, the solvent-accessible area is lower between 0–40 ns.

**Table S1** The amino acid residues that allow Ig-like domain interaction are listed below and the SAS change of each amino acid can be observed.

| **0 ns** | | | | | | **2 ns** | | | | | |
| --- | --- | --- | --- | --- | --- | --- | --- | --- | --- | --- | --- |
| **α Subunit** | | | **β3 Subunit** | | | **α Subunit** | | | **β3 Subunit** | | |
| **Residue** | **Number** | **SAS Area** | **Residue** | **Number** | **SAS Area** | **Residue** | **Number** | **SAS Area** | **Residue** | **Number** | **SAS Area** |
| GLU | 307 | 9.0366 | ARG | 139 | 7.0404 | GLN | 1378 | 21.218 | GLU | 28 | 29.418 |
| ASP | 1677 | 5.3821 | CYS | 26 | 17.556 | LYS | 1220 | 66.71 | ARG | 51 | 3.2166 |
| TYR | 1668 | 93.116 | GLU | 32 | 25.926 | ARG | 1218 | 52.161 | VAL | 29 | 38.751 |
| VAL | 1669 | 2.5703 | ARG | 51 | 68.436 | GLU | 1217 | 59.525 | GLU | 52 | 131.58 |
| LYS | 1671 | 70.201 | VAL | 54 | 5.7595 | LYS | 1287 | 5.2733 | PHE | 23 | 77.345 |
| GLU | 1672 | 21.605 | GLU | 53 | 81.635 | SER | 1664 | 16.723 | VAL | 25 | 69.829 |
| LYS | 1670 | 67.206 | LYS | 50 | 63.575 | GLU | 1672 | 2.9295 | VAL | 27 | 34.515 |
| GLY | 1728 | 14.473 | VAL | 27 | 46.672 | TYR | 1668 | 102.76 | MET | 49 | 40.11 |
| ASN | 1732 | 9.9586 | VAL | 29 | 30.7 | LYS | 1671 | 2.455 | LYS | 50 | 12.37 |
| LYS | 1719 | 74.459 | GLU | 52 | 90.406 | ASN | 1665 | 20.295 | ILE | 46 | 3.1457 |
| PRO | 1722 | 96.032 | LYS | 42 | 3.0232 | LYS | 1670 | 37.692 | PRO | 24 | 72.132 |
| VAL | 1726 | 36.573 | GLU | 28 | 51.527 | HIS | 1721 | 48.097 | GLU | 55 | 19.723 |
| PRO | 1713 | 8.5498 | PRO | 30 | 52.907 | VAL | 1720 | 46.918 | SER | 31 | 0.82554 |
| ASP | 1714 | 0.93524 | PRO | 24 | 87.68 | ASP | 1714 | 1.5109 | GLU | 32 | 32.428 |
| VAL | 1720 | 2.0945 | PHE | 23 | 96.126 | ASP | 1716 | 32.719 | CYS | 26 | 37.499 |
| ASP | 1729 | 61.278 | SER | 31 | 5.1735 | SER | 1724 | 7.7382 | PRO | 30 | 69.746 |
| CYS | 1730 | 6.147 | MET | 49 | 2.6469 | GLY | 1723 | 6.8507 | GLU | 53 | 40.592 |
| ASP | 1716 | 1.945 | THR | 33 | 4.694 | CYS | 1715 | 3.452 | PHE | 128 | 20.808 |
| GLY | 1731 | 5.6495 | VAL | 25 | 58.778 | GLY | 1728 | 0.86604 | ARG | 139 | 0.89402 |
| HIS | 1721 | 51.705 | GLU | 129 | 128.14 | LYS | 1718 | 28.676 | ASP | 102 | 10.189 |
| GLU | 1727 | 15.891 | SER | 97 | 20.746 | PRO | 1713 | 43.228 | LYS | 98 | 36.561 |
| CYS | 1715 | 17.322 | LEU | 100 | 0.81147 | LYS | 1719 | 52.19 | ASP | 99 | 17.724 |
| GLY | 1723 | 5.9767 | ASP | 99 | 12.327 | VAL | 1726 | 20.576 | GLN | 101 | 15.016 |
| SER | 1724 | 0.74269 | ASN | 95 | 1.2536 | ASP | 1729 | 28.858 | SER | 97 | 28.928 |
| LYS | 1223 | 2.1016 | GLY | 96 | 0.56655 |  |  |  |  |  |  |
| ARG | 1218 | 71.49 | GLN | 101 | 43.003 |  |  |  |  |  |  |
| LYS | 1220 | 74.043 | ASP | 102 | 26.73 |  |  |  |  |  |  |
| ILE | 1214 | 2.1143 | ALA | 130 | 19.084 |  |  |  |  |  |  |
| GLU | 1217 | 85.058 | HIS | 131 | 9.1579 |  |  |  |  |  |  |
| **10 ns** | | | | | | **20 ns** | | | | | |
| **α Subunit** | | | **β3 Subunit** | | | **α Subunit** | | | **β3 Subunit** | | |
| **Residue** | **Number** | **SAS Area** | **Residue** | **Number** | **SAS Area** | **Residue** | **Number** | **SAS Area** | **Residue** | **Number** | **SAS Area** |
| ILE | 1214 | 22.731 | PHE | 23 | 108.56 | GLU | 326 | 64.902 | PRO | 30 | 17.172 |
| GLU | 1217 | 11.567 | VAL | 27 | 23.418 | ILE | 1216 | 1.054 | PRO | 24 | 69.607 |
| ARG | 1218 | 80.436 | VAL | 25 | 87.702 | ARG | 1218 | 78.419 | GLU | 28 | 12.137 |
| LYS | 1220 | 25.564 | CYS | 26 | 37.115 | ILE | 1214 | 56.802 | GLU | 32 | 14.876 |
| LYS | 1287 | 25.021 | PRO | 24 | 113.55 | LYS | 1220 | 15.133 | CYS | 26 | 41.232 |
| ARG | 1290 | 1.0301 | GLU | 28 | 26.667 | ASP | 1213 | 12.059 | ILE | 46 | 1.1717 |
| ASP | 1282 | 39.402 | VAL | 29 | 13.157 | GLU | 1217 | 51.002 | VAL | 25 | 83.894 |
| SER | 1281 | 1.9192 | PRO | 30 | 25.636 | LYS | 1287 | 0.24928 | MET | 49 | 6.8362 |
| VAL | 1376 | 36.104 | GLU | 32 | 15.04 | ARG | 1381 | 39.697 | LYS | 50 | 92.017 |
| GLN | 1378 | 94.406 | ILE | 46 | 1.135 | GLN | 1378 | 52.378 | ARG | 51 | 50.033 |
| SER | 1377 | 0.9391 | ARG | 51 | 31.739 | VAL | 1376 | 16.909 | GLU | 52 | 151.31 |
| ASN | 1375 | 20.733 | SER | 97 | 26.418 | SER | 1377 | 12.56 | GLU | 53 | 78.353 |
| ARG | 1381 | 12.241 | ASP | 99 | 22.827 | ASN | 1379 | 41.771 | VAL | 27 | 42.94 |
| PRO | 1713 | 43.212 | LYS | 50 | 28.345 | ALA | 1667 | 15.104 | VAL | 29 | 30.935 |
| LYS | 1670 | 54.335 | GLN | 101 | 27.809 | TYR | 1668 | 75.075 | PHE | 23 | 147.4 |
| GLU | 1672 | 47.009 | GLU | 52 | 145.46 | LYS | 1671 | 20.324 | ASP | 99 | 26.286 |
| ASP | 1714 | 4.2577 | GLU | 53 | 48.212 | GLU | 1672 | 65.891 | GLN | 101 | 19.766 |
| TYR | 1668 | 82.037 | MET | 49 | 54.353 | LYS | 1670 | 58.148 | ASP | 102 | 19.224 |
| GLY | 1728 | 0.50842 | ASP | 102 | 14.138 | GLU | 1682 | 2.6102 | LYS | 98 | 19.735 |
| VAL | 1720 | 30.046 | PHE | 128 | 34.155 | LYS | 1718 | 0.98077 | SER | 97 | 25.91 |
| VAL | 1726 | 23.936 | ALA | 130 | 8.9658 | ASP | 1729 | 5.2973 | GLY | 96 | 2.0194 |
| GLY | 1723 | 11.003 | HIS | 131 | 56.135 | GLY | 1723 | 22.319 | ALA | 130 | 47.717 |
| HIS | 1721 | 44.118 | PRO | 133 | 11.273 | VAL | 1720 | 41.833 | PHE | 128 | 44.716 |
| ASP | 1716 | 34.254 | GLU | 129 | 16.551 | PRO | 1722 | 106.79 | THR | 138 | 1.5367 |
|  |  |  |  |  |  | SER | 1724 | 23.778 | PRO | 133 | 1.1232 |
|  |  |  |  |  |  | ASP | 1716 | 46.65 | LYS | 136 | 2.4657 |
|  |  |  |  |  |  | PRO | 1712 | 1.233 | HIS | 131 | 37.44 |
|  |  |  |  |  |  | ASP | 1714 | 14.194 | THR | 137 | 15.797 |
|  |  |  |  |  |  | LYS | 1719 | 36.427 | ARG | 139 | 25.069 |
| **30 ns** | | | | | | **40 ns** | | | | | |
| **α Subunit** | | | **β3 Subunit** | | | **α Subunit** | | | **β3 Subunit** | | |
| **Residue** | **Number** | **SAS Area** | **Residue** | **Number** | **SAS Area** | **Residue** | **Number** | **SAS Area** | **Residue** | **Number** | **SAS Area** |
| LYS | 1287 | 10.687 | GLU | 28 | 6.41 | GLU | 307 | 10.49 | THR | 137 | 14.699 |
| GLU | 1217 | 53.212 | ILE | 46 | 0.81391 | GLU | 326 | 36.541 | LYS | 136 | 0.019012 |
| ARG | 1218 | 90.859 | GLU | 53 | 48.726 | LYS | 1671 | 1.4686 | ARG | 139 | 16.465 |
| LYS | 1220 | 16.197 | CYS | 26 | 41.069 | TYR | 1668 | 82.14 | LYS | 98 | 36.889 |
| ILE | 1214 | 57.455 | PRO | 30 | 18.887 | GLU | 1672 | 43.288 | GLN | 101 | 32.218 |
| ASP | 1213 | 3.6012 | MET | 49 | 5.5305 | LYS | 1670 | 13.297 | ASP | 99 | 20.01 |
| GLN | 1378 | 64.268 | VAL | 54 | 0.24388 | ALA | 1667 | 13.199 | PHE | 128 | 29.153 |
| ARG | 1381 | 32.782 | LYS | 50 | 18.363 | GLU | 1682 | 1.5089 | SER | 97 | 22.83 |
| GLU | 326 | 38.748 | ARG | 51 | 58.238 | HIS | 1721 | 57.237 | ASP | 102 | 13.41 |
| ALA | 1667 | 11.784 | GLU | 52 | 147.75 | CYS | 1715 | 6.618 | VAL | 25 | 74.932 |
| VAL | 1720 | 31.255 | PRO | 24 | 94.076 | ASP | 1714 | 21.365 | CYS | 26 | 31.788 |
| HIS | 1721 | 53.67 | VAL | 27 | 35.225 | GLY | 1723 | 31.768 | GLU | 32 | 42.286 |
| CYS | 1715 | 9.8986 | VAL | 29 | 28.97 | PRO | 1713 | 9.9038 | GLU | 28 | 12.883 |
| ASP | 1716 | 30.755 | VAL | 25 | 83.187 | ASP | 1716 | 31.822 | VAL | 29 | 32.166 |
| GLY | 1723 | 33.006 | PHE | 23 | 154.44 | VAL | 1720 | 56.46 | PRO | 30 | 19.53 |
| SER | 1724 | 23.423 | GLU | 32 | 23.322 | LYS | 1718 | 38.413 | VAL | 27 | 35.446 |
| ASP | 1714 | 14.207 | ARG | 44 | 7.1425 | PRO | 1722 | 83.656 | PHE | 23 | 143.29 |
| TYR | 1668 | 72.485 | GLU | 55 | 23.647 | SER | 1724 | 21.999 | PRO | 24 | 95.328 |
| GLU | 1672 | 35.946 | ASP | 99 | 17.279 | ASP | 1729 | 4.0441 | VAL | 54 | 8.7031 |
| LYS | 1670 | 67.034 | GLN | 101 | 24.572 | LYS | 1719 | 80.765 | GLU | 52 | 94.333 |
| PRO | 1713 | 13.075 | LYS | 98 | 2.3188 | ASP | 1213 | 5.2921 | LYS | 50 | 1.7836 |
| LYS | 1719 | 65.715 | SER | 97 | 24.851 | GLU | 1217 | 51.505 | ARG | 51 | 77.27 |
| GLY | 1728 | 0.7567 | ASP | 102 | 11.765 | LYS | 1220 | 42.873 | GLU | 55 | 22.839 |
| ASP | 1729 | 13.289 | THR | 137 | 11.429 | ARG | 1218 | 94.737 | MET | 49 | 6.6199 |
| PRO | 1722 | 88.345 | HIS | 131 | 9.2664 | ILE | 1214 | 59.201 | GLU | 53 | 76.199 |
| **50 ns** | | | | | | **60 ns** | | | | | |
| **α Subunit** | | | **β3 Subunit** | | | **α Subunit** | | | **β3 Subunit** | | |
| **Residue** | **Number** | **SAS Area** | **Residue** | **Number** | **SAS Area** | **Residue** | **Number** | **SAS Area** | **Residue** | **Number** | **SAS Area** |
| ALA | 1667 | 14.424 | VAL | 54 | 24.793 | ARG | 1218 | 79.972 | PHE | 126 | 0.47374 |
| TYR | 1668 | 80.503 | VAL | 29 | 33.984 | ILE | 1214 | 23.682 | PHE | 128 | 82.04 |
| LYS | 1670 | 20.32 | PHE | 23 | 154.61 | THR | 1354 | 5.6784 | ARG | 139 | 33.137 |
| LYS | 1671 | 16.163 | PRO | 24 | 91.442 | SER | 1377 | 24.514 | GLU | 129 | 19.884 |
| GLU | 1672 | 34.506 | GLU | 28 | 7.9174 | GLN | 1378 | 136.55 | ALA | 130 | 34.937 |
| SER | 1724 | 26.427 | PRO | 30 | 9.9971 | ASN | 1375 | 0.70722 | THR | 137 | 10.396 |
| ASP | 1716 | 32.309 | LYS | 50 | 26.475 | ASN | 1379 | 59.548 | GLU | 127 | 0.34353 |
| ASP | 1714 | 12.647 | GLU | 52 | 104.94 | VAL | 1380 | 0.10894 | GLU | 28 | 3.0835 |
| ASP | 1729 | 7.9979 | CYS | 26 | 37.576 | ARG | 1381 | 29.11 | GLU | 52 | 107.5 |
| CYS | 1715 | 12.355 | VAL | 27 | 28.907 | LYS | 1383 | 13.617 | MET | 49 | 0.34459 |
| LYS | 1719 | 74.182 | GLU | 55 | 20.4 | VAL | 1376 | 60.4 | PRO | 30 | 11.28 |
| CYS | 1730 | 0.03277 | VAL | 25 | 84.196 | THR | 1353 | 38.196 | LYS | 50 | 63.918 |
| PRO | 1712 | 4.1217 | ILE | 46 | 1.6322 | TYR | 1668 | 90.987 | GLU | 55 | 28.044 |
| GLU | 1682 | 0.14425 | MET | 49 | 1.6193 | LYS | 1670 | 18.091 | CYS | 26 | 34.867 |
| VAL | 1720 | 56.558 | ARG | 51 | 102.78 | LYS | 1671 | 4.3491 | VAL | 25 | 71.748 |
| HIS | 1721 | 54.449 | GLU | 53 | 127.71 | VAL | 1669 | 0.18463 | VAL | 27 | 21.558 |
| PRO | 1713 | 24.86 | PHE | 128 | 41.349 | GLU | 1672 | 47.041 | GLU | 53 | 133.37 |
| GLY | 1723 | 32.305 | GLU | 129 | 37.125 | GLU | 1682 | 2.5069 | PHE | 23 | 109.28 |
| LYS | 1718 | 17.728 | GLN | 101 | 26.685 | ALA | 1667 | 1.7263 | ILE | 46 | 1.6947 |
| PRO | 1722 | 101.37 | SER | 97 | 27.025 | ASP | 1716 | 32.226 | ARG | 51 | 121.58 |
| ARG | 1218 | 74.621 | ASP | 102 | 19.138 | HIS | 1721 | 66.282 | VAL | 29 | 32.453 |
| ILE | 1214 | 47.946 | ALA | 130 | 41.285 | ASN | 1709 | 3.5529 | VAL | 54 | 29.651 |
| GLU | 1217 | 52.662 | LYS | 98 | 28.395 | ASP | 1714 | 13.105 | PRO | 24 | 77.833 |
| ASP | 1213 | 4.0705 | ASP | 99 | 14.587 | PRO | 1722 | 116.85 | SER | 97 | 20.039 |
|  |  |  |  |  |  | GLY | 1723 | 36.356 | LYS | 98 | 45.797 |
|  |  |  |  |  |  | VAL | 1720 | 46.622 | ASP | 99 | 20.988 |
|  |  |  |  |  |  | PRO | 1712 | 1.232 | GLN | 101 | 25.954 |
|  |  |  |  |  |  | SER | 1724 | 22.036 | ASP | 102 | 18.476 |
| **70 ns** | | | | | | **80 ns** | | | | | |
| **α Subunit** | | | **β3 Subunit** | | | **α Subunit** | | | **β3 Subunit** | | |
| **Residue** | **Number** | **SAS Area** | **Residue** | **Number** | **SAS Area** | **Residue** | **Number** | **SAS Area** | **Residue** | **Number** | **SAS Area** |
| GLY | 1723 | 3.2467 | VAL | 29 | 39.67 | GLU | 326 | 17.831 | GLU | 53 | 140.06 |
| GLY | 1728 | 3.9872 | CYS | 26 | 35.366 | TYR | 304 | 0.02267 | GLU | 55 | 34.19 |
| ASN | 1709 | 1.4776 | VAL | 25 | 74.518 | GLU | 307 | 24.606 | GLU | 52 | 123.27 |
| PRO | 1722 | 113 | PHE | 23 | 134.07 | TYR | 1668 | 94.332 | VAL | 25 | 78.186 |
| ASP | 1714 | 7.3297 | PRO | 24 | 78.821 | GLU | 1682 | 0.00466 | PHE | 23 | 139.27 |
| CYS | 1715 | 5.7583 | VAL | 27 | 28.181 | LYS | 1671 | 6.9644 | PRO | 24 | 90.887 |
| LYS | 1718 | 9.8034 | PRO | 30 | 16.451 | LYS | 1670 | 55.686 | GLU | 32 | 8.0467 |
| PRO | 1713 | 24.876 | GLU | 32 | 15.719 | ALA | 1667 | 3.8389 | GLU | 28 | 8.0137 |
| ASP | 1716 | 32.341 | GLU | 53 | 130.86 | GLU | 1672 | 52.848 | ARG | 44 | 0.029244 |
| LYS | 1719 | 60.478 | ARG | 51 | 96.917 | THR | 1354 | 20.697 | PRO | 30 | 40.628 |
| HIS | 1721 | 54.683 | VAL | 54 | 31.324 | VAL | 1376 | 68.284 | LYS | 50 | 85.219 |
| SER | 1724 | 19.037 | ILE | 46 | 0.38001 | SER | 1377 | 22.97 | ILE | 46 | 1.7154 |
| VAL | 1720 | 34.381 | GLU | 52 | 124.65 | ASN | 1379 | 50.229 | CYS | 26 | 38.28 |
| ASP | 1729 | 15.605 | LYS | 50 | 103.42 | ARG | 1381 | 28.553 | MET | 49 | 4.8935 |
| VAL | 1726 | 29.388 | GLU | 55 | 23.883 | ASN | 1375 | 21.701 | VAL | 27 | 27.34 |
| TYR | 1668 | 85.196 | PHE | 128 | 66.03 | GLN | 1378 | 119.51 | VAL | 29 | 18.935 |
| ALA | 1667 | 6.6768 | ASP | 99 | 12.702 | THR | 1353 | 34.392 | ARG | 51 | 110.69 |
| LYS | 1671 | 37.839 | GLN | 101 | 20.412 | ILE | 1216 | 0.30018 | VAL | 54 | 27.095 |
| LYS | 1670 | 60.966 | GLY | 96 | 0.03133 | ASP | 1213 | 1.8085 | ASP | 99 | 20.835 |
| ASP | 1677 | 1.8642 | GLU | 127 | 11.275 | GLU | 1217 | 51.266 | GLU | 127 | 5.5181 |
| GLU | 1672 | 55.223 | GLU | 129 | 40.544 | ILE | 1214 | 53.146 | PRO | 133 | 1.0588 |
| ILE | 1216 | 1.1984 | ASP | 102 | 16.702 | ARG | 1218 | 67.519 | ASP | 102 | 14.623 |
| GLU | 1217 | 46.239 | SER | 97 | 21.874 | TYR | 1215 | 17.615 | PHE | 128 | 83.041 |
| ARG | 1218 | 83.92 | LYS | 98 | 22.695 | LYS | 1220 | 6.3026 | ALA | 130 | 27.219 |
| TYR | 1215 | 0.31316 | THR | 137 | 2.6063 | CYS | 1715 | 6.7561 | GLU | 129 | 34.176 |
| ILE | 1214 | 49.502 | LEU | 140 | 0.7878 | VAL | 1720 | 24.446 | THR | 138 | 8.7614 |
| ASP | 1213 | 1.0505 | ARG | 139 | 36.573 | SER | 1724 | 26.572 | LEU | 140 | 8.5301 |
| THR | 1354 | 2.798 | THR | 138 | 5.117 | ASP | 1714 | 10.876 | GLN | 101 | 25.422 |
|  |  |  |  |  |  | PRO | 1713 | 19.15 | HIS | 131 | 5.7492 |
|  |  |  |  |  |  | LYS | 1718 | 14.221 | LYS | 98 | 6.6113 |
|  |  |  |  |  |  | HIS | 1721 | 48.966 | SER | 97 | 26.186 |
| **90 ns** | | | | | | **100 ns** | | | | | |
| **α Subunit** | | | **β3 Subunit** | | | **α Subunit** | | | **β3 Subunit** | | |
| **Residue** | **Number** | **SAS Area** | **Residue** | **Number** | **SAS Area** | **Residue** | **Number** | **SAS Area** | **Residue** | **Number** | **SAS Area** |
| ILE | 1214 | 30.183 | PHE | 126 | 0.00274 | ARG | 1218 | 72.84 | GLU | 129 | 12.227 |
| ILE | 1216 | 4.4394 | PHE | 128 | 76.358 | TYR | 1215 | 0.13687 | PRO | 133 | 0.73235 |
| ARG | 1218 | 66.155 | GLU | 127 | 9.0624 | ILE | 1214 | 59.268 | ASP | 102 | 16.608 |
| GLU | 1217 | 30.723 | GLU | 129 | 36.341 | GLU | 1217 | 15.617 | GLN | 101 | 34.045 |
| ARG | 1381 | 29.664 | ALA | 130 | 34.46 | THR | 1353 | 35.101 | ASP | 99 | 11.544 |
| THR | 1354 | 2.965 | GLN | 101 | 21.353 | THR | 1354 | 19.49 | GLU | 127 | 7.6698 |
| LYS | 1383 | 13.391 | ASP | 102 | 14.471 | LYS | 1383 | 5.4915 | PHE | 128 | 81.793 |
| ASN | 1379 | 18.566 | SER | 97 | 29.844 | ARG | 1381 | 40.341 | PHE | 126 | 4.7689 |
| ASN | 1375 | 11.497 | GLU | 53 | 128.58 | ASN | 1379 | 28.693 | SER | 97 | 8.5846 |
| VAL | 1376 | 69.611 | ASP | 99 | 11.23 | GLN | 1378 | 146.17 | GLU | 28 | 1.2752 |
| GLN | 1378 | 122.04 | VAL | 54 | 20.796 | SER | 1377 | 18.361 | LYS | 50 | 77.588 |
| SER | 1377 | 20.046 | GLU | 55 | 35.389 | VAL | 1376 | 23.803 | ARG | 51 | 107.73 |
| THR | 1353 | 37.413 | ILE | 46 | 0.08741 | GLU | 326 | 85.94 | VAL | 25 | 77.512 |
| ASP | 1729 | 13.106 | MET | 49 | 3.2378 | GLU | 307 | 24.816 | VAL | 27 | 35.555 |
| GLY | 1728 | 5.2792 | LYS | 50 | 69.641 | ALA | 1667 | 6.5054 | ILE | 46 | 9.8877 |
| ALA | 1667 | 4.7551 | ARG | 51 | 72.315 | TYR | 1668 | 99.339 | GLU | 52 | 126.73 |
| TYR | 1668 | 90.201 | CYS | 26 | 40.55 | LYS | 1670 | 52.546 | PRO | 30 | 23.893 |
| LYS | 1670 | 51.266 | GLU | 52 | 122.63 | LYS | 1671 | 8.9168 | GLU | 53 | 128.42 |
| GLU | 1672 | 53.221 | VAL | 25 | 83.651 | GLU | 1672 | 50.988 | VAL | 54 | 30.676 |
| LYS | 1671 | 23.246 | PRO | 24 | 79.63 | GLU | 1682 | 3.5159 | GLU | 55 | 20.249 |
| HIS | 1721 | 55.943 | VAL | 27 | 18.641 | PRO | 1713 | 23.045 | PRO | 24 | 88.953 |
| ASP | 1714 | 11.04 | VAL | 29 | 29.027 | ASP | 1714 | 6.4745 | VAL | 29 | 40.857 |
| VAL | 1726 | 27.903 | GLU | 28 | 4.8677 | CYS | 1715 | 10.142 | PHE | 23 | 113.65 |
| ASP | 1716 | 30.52 | PRO | 30 | 21.507 | LYS | 1718 | 7.2682 | MET | 49 | 5.7241 |
| PRO | 1713 | 11.344 | PHE | 23 | 121.4 | VAL | 1720 | 18.401 | CYS | 26 | 35.007 |
|  |  |  |  |  |  | ASP | 1716 | 34.787 | THR | 138 | 29.211 |
|  |  |  |  |  |  | LYS | 1719 | 51.388 | ARG | 139 | 8.6733 |
|  |  |  |  |  |  | HIS | 1721 | 58.761 | THR | 137 | 16.363 |
|  |  |  |  |  |  | PRO | 1722 | 119.74 | LYS | 136 | 0.57202 |

**Part 2: Molecular modeling of 3D models**


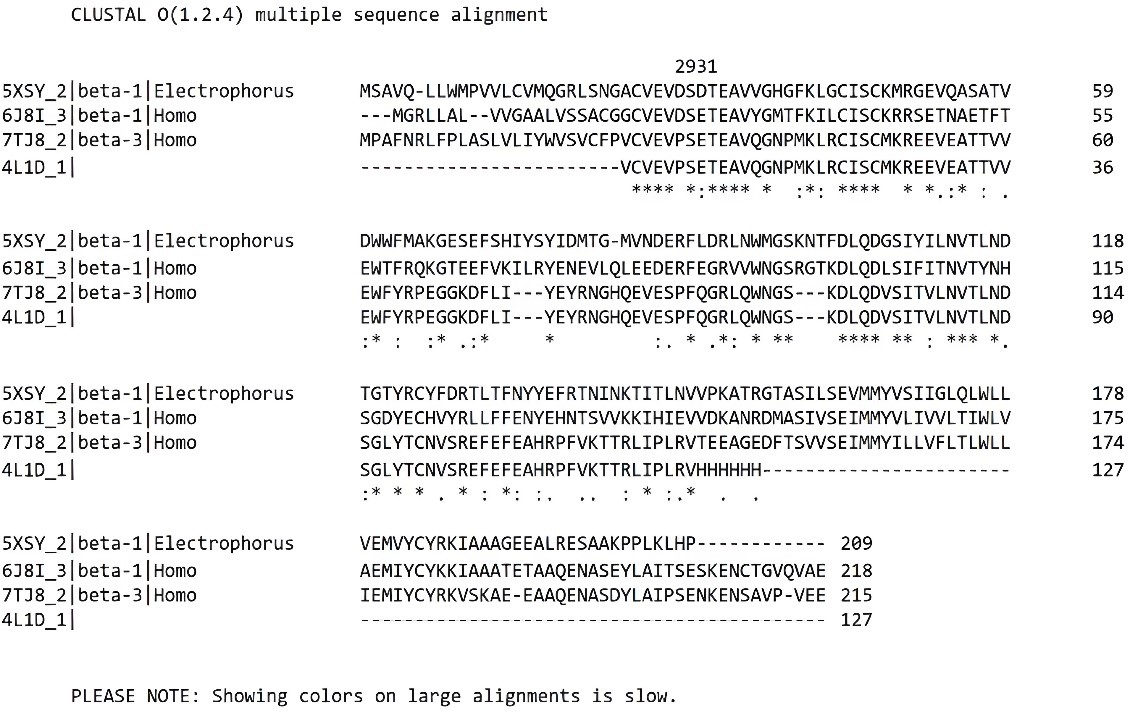


**Fig. S3** Multiple sequence alignment of β1 and β3 subunit ectodomains. The comparison reveals that aspartate D31 only exist in eel β1 subunit and this amino acid is equivalent to glutamate E27 on hβ1. Moreover, on hβ1 residue D25 has mutated to proline P30 on hβ3.

**Table S2** Chronologic listing of all hitherto available PDB entries (<https://www.rcsb.org/>). Last visit 02.12.2024. The grey bar between row 6NT4 and 7TJ8 separates the PDB entries known at the beginning of this study in 2021 from those published during the ongoing study until December 2023. Abbreviation: EM = Electron Microscopy. All sequences were collected for *Homo sapiens* with two exceptions: 5XSY (2017) from *Electrophorus electricus* and 6NT4 (2019) from *Periplaneta americana.*

| **PDB entry (year)** | **Subunit α ligand if any** | | | **Subunit (s) β** | | **Method Resolution**  **in [Å]** | | | **References** |
| --- | --- | --- | --- | --- | --- | --- | --- | --- | --- |
|  |  | | |  | |  | | |  |
| **4JPZ (2014)** | Na_v_1.2 C-terminal | | | No β | | X-ray diffraction | | | URL. <https://www.rcsb.org/structure/4JPZ> |
|  |  |  |  |  |  |  |  |  | P ub1: [1] |
|  | Calmodulin/Ca^2+^ | | |  |  | 3 | | | Pub2: [2] |
| **4L1D (2014)** | No α | | | β3 Incomplete Extracelular (Ig-like) (all beta sheet) | | X-ray diffraction 2.5 | | | URL <https://www.rcsb.org/structure/4L1D> |
|  |  |  |  |  |  |  |  |  | Pub1: [3] |
|  |  |  |  |  |  |  |  |  | Pub2: [4] |
| 5XSY (2017) | Na_v_1.4 | | | β1 | | EM  4 | | | <https://www.rcsb.org/structure/5XSY> |
|  |  |  |  |  |  |  |  |  | Pub1: [5] |
|  |  |  |  |  |  |  |  |  | Pub2: [6] |
| 6AGF (2018) | Na_v_1.4 | | | β1 Complete (Extracellular + transmembranal) | | EM  3.2 | | | URL <https://www.rcsb.org/structure/6AGF> |
|  |  |  |  |  |  |  |  |  | Pub1: [7] |
|  |  |  |  |  |  |  |  |  | Pub2: [8] |
| 6J8J (2019) | Na_v_1.7 | | | β1 Complete (Extracellular + transmembranal) | | EM  3.2 | | | URL: <https://www.rcsb.org/structure/6J8J> |
|  | Lig. ProTx-II | | |  |  |  |  |  | Pub1: [9] |
|  | Lig. Tetrodotoxin | | | β2 Incomplete Extracellular (Ig-like) (all beta sheet) | |  |  |  | Pub2: [8] |
| 6J8H (2019) | Na_v_1.7 | | | β1 Complete (Extracellular + transmembranal) | | EM  3.2 | | | URL <https://www.rcsb.org/structure/6J8H> |
|  | Lig. Huwentoxin-IV | | | β2 Incomplete Extracellular (Ig-like) (all beta sheet) | |  |  |  | Pub1: [9] |
|  | Lig. Saxitoxin | | |  |  |  |  |  | Pub2: [10] |
|  |  |  |  |  |  |  |  |  | Pub3: [11] |
| **6J8I (2019)** | Na_v_1.7 | | | β1 Complete (Extracellular + transmembranal) | | EM  3.2 | | | URL <https://www.rcsb.org/structure/6J8I> |
|  | Lig ProTx-II | | |  |  |  |  |  | Pub1: [9] |
|  | Lig. Tetrodotoxin | | | β2 Incomplete Extracellular (Ig-like) (all beta sheet) | |  |  |  | Pub2: [12] |
|  |  |  |  |  |  |  |  |  | Pub3: [13] |
| 6J8G (2019) | | | Na_v_1.7 | | β1 Complete (Extracellular + transmembranal) | EM  3.2 | | URL <https://www.rcsb.org/structure/6J8G> | |
|  |  |  | Lig Huwetoxin IV | |  |  |  | Pub1: [9] | |
|  |  |  | Lig. Saxitoxin | | β2 Incomplete Extracellular (Ig-like) (all beta sheet) |  |  | Pub2: [14] | |
| 6J8E (2019) | | | Na_v_1.2 | |  | EM  3 | | URL <https://www.rcsb.org/structure/6J8E> | |
|  |  |  |  |  |  |  |  | Pub1: [15] | |
|  |  |  | Lig. μ-conotoxin KIIA | |  |  |  | Pub2: [16] | |
| 6NT4 (2019) | | | Na_v_ Human-cockroach | | No β | EM  3.5 | | URL. <https://www.rcsb.org/structure/6NT4> | |
|  |  |  | Lig. α scorpion toxin | |  |  |  | Pub1. [17] | |
|  | | |  | |  |  | |  | |
| 7TJ8 (2022) | | | Na_x_ | | β3 Complete (Extracellular + transmembranal | EM  3.2 | | URL <https://www.rcsb.org/structure/7TJ8> | |
|  |  |  |  |  |  |  |  | Pub1: [18] | |
|  |  |  |  |  |  |  |  | Pub2: [19] | |
| 7TJ9 (2022) | | | Na_x_ | | β3 Complete (Extracellular + transmembranal | EM  2.9 | | URL <https://www.rcsb.org/structure/7TJ9> | |
|  |  |  |  |  |  |  |  | Pub1: [18] | |
| 7XM9 (2022) | | | Na_v_1.7 | | β1 Complete (Extracellular + transmembranal) | EM  3.2 | | URL <https://www.rcsb.org/structure/7XM9> | |
|  |  |  | Lig. XEN907 | | β2 Incomplete Extracellular (Ig-like) (all beta sheet) |  |  | Pub1: [20] | |
| 7W7F (2022) | | | Na_v_1.3 | | β1 Complete (Extracellular + transmembranal) | EM  3.6 | | URL <https://www.rcsb.org/structure/7W7F> | |
|  |  |  |  |  |  |  |  | Pub1: [21] | |
|  |  |  | Lig. ICA-121431 | | β2 Incomplete Extracellular (Ig-like) (all beta sheet) |  |  | Pub2: [22] | |
| 7W9T (2022) | | | Na_v_1.7 | | β1 Complete (Extracellular + transmembranal) | EM  3 | | URL <https://www.rcsb.org/structure/7W9T> | |
|  |  |  | Lig. Huwentoxin IV | |  |  |  | Pub1: [23] | |
|  |  |  | Lig. Saxitoxin | | β2 Incomplete Extracellular (Ig-like) (all beta sheet) |  |  | Pub2: [14] | |
| 7W77 (2022) | | | Na_v_1.3 | | β1 Complete (Extracellular + transmembranal | EM  3.3 | | URL <https://www.rcsb.org/structure/7W77> | |
|  |  |  |  |  |  |  |  | Pub1: [15] | |
|  |  |  | Lig. Bulleyaconitine A | | β2 Incomplete Extracellular (Ig-like) (all beta sheet) |  |  | Pub2: [13] | |
| 7W9L (2022) | | | Na_v_1.7 | | β1 Complete (Extracellular + transmembranal) | EM  3.5 | | URL <https://www.rcsb.org/structure/7W9L> | |
|  |  |  |  |  | β2 Incomplete Extracellular (Ig-like) (all beta sheet) |  |  | Pub1: [23] | |
| 7WEL (2022) | | | Na_v_1.8 | | No β | EM  3.2 | | URL <https://www.rcsb.org/structure/7WEL> | |
|  |  |  |  |  |  |  |  | Pub1: [24] | |
|  |  |  | Lig A-803467 | |  |  |  | Pub2: [25] | |
| 7XVF (2022) | | | Na_v_1.7 | | β1 Complete (Extracellular + transmembranal) | EM  2.8 | | URL <https://www.rcsb.org/structure/7XVF> | |
|  |  |  |  |  |  |  |  | Pub1: [26] | |
|  |  |  |  |  | β2 Incomplete Extracellular (Ig-like) (all beta sheet) |  |  | Pub2: [27] | |
| 7W9M (2022) | | | Na_v_1.7 | | β1 Complete (Extracellular + transmembranal) | EM  3 | | URL. <https://www.rcsb.org/structure/7W9M> | |
|  |  |  | Lig. ProTx-II | |  |  |  | Pub1: [23] | |
|  |  |  | Lig. Tetrodotoxin | | β2 Incomplete Extracellular (Ig-like) (all beta sheet) |  |  |  |  |
| 7XMG (2022) | | | Na_v_1.7 | | β1 Complete (Extracellular + transmembranal) | EM  3.1 | | URL Chain: <https://www.rcsb.org/structure/7XMG> | |
|  |  |  |  |  |  |  |  | Pub1: [20] | |
|  |  |  | Lig. TCN-1752 | | β2 Incomplete Extracellular (Ig-like) (all beta sheet) |  |  | Pub2: [14] | |
| 7WFR (2022) | | | Na_v_1.8 | | No β | EM  3 | | URL <http://www.rcsb.org/structure/7WFR> | |
|  |  |  | Lig. A-803467 | |  |  |  | Pub1: [24] | |
| 7WE4 (2022) | | | Na_v_1.8 | | No β | EM  2.5 | | URL <https://www.rcsb.org/structure/7WE4> | |
|  |  |  | Lig. A-803467 | |  |  |  | Pub1: [24] | |
| 7W9K (2022) | | Na_v_1.7 | | | β1 Complete (Extracellular + transmembranal) | EM  2.2 | | URL: <https://www.rcsb.org/structure/7W9K> | |
|  |  |  |  |  | β2 Incomplete Extracellular (Ig-like) (all beta sheet) |  |  | Pub1: [23] | |
| 8THG (2023) | | Na_v_1.7 | | | β1 Complete (Extracellular + transmembranal) | EM  2.9 | | URL <https://www.rcsb.org/structure/8THG> | |
|  |  | Lig. RLZ | | | β2 Incomplete Extracellular (Ig-like) (all beta sheet) |  |  | Pub1: [28] | |
| 8GZ1 (2023) | | Na_v_1.6 | | | β1 Complete (Extracellular + transmembranal) | EM  3.4 | | URL: <https://www.rcsb.org/structure/8GZ1> | |
|  |  |  |  |  | β2 Incomplete Extracellular (Ig-like) (all beta sheet) |  |  | Pub1: [29] | |
| 8THH (2023) | | Na_v_1.7 | | | β1 Complete (Extracellular + transmembranal) | EM  2.7 | | URL <https://www.rcsb.org/structure/8THH> | |
|  |  | Lig. LTG | | | β2 Incomplete Extracellular (Ig-like) (all beta sheet) |  |  | Pub1: [27] | |
| 8GZ2 (2023) | | Na_v_1.6 | | | β1 Complete (Extracellular + transmembranal) | EM  3.4 | | URL: <https://www.rcsb.org/structure/8GZ1> | |
|  |  | Lig. Anhidro-tetrodotoxin | | | β2 Incomplete Extracellular (Ig-like) (all beta sheet) |  |  | Pub1: [29] | |
| 8S9B (2023) | | Na_v_1.7 | | | β1 Complete (Extracellular + transmembranal) | EM  2.9 | | URL <https://www.rcsb.org/structure/8S9B> | |
|  |  | Lig. LCM | | | β2 Extracellular (Ig-like) (all beta sheet) |  |  | Pub1: [42] | |
| 8FHD (2023) | | Na_v_1.6 | | | β1 Complete (Extracellular + transmembranal) | EM  3.1 | | URL: <https://www.rcsb.org/structure/8FHD> | |
|  |  |  |  |  |  |  |  | Pub1: [7] | |
| 8I5Y (2023) | | Na_v_1.7 | | | β1 Complete (Extracellular + transmembranal) | | EM  2.6 | URL <https://www.rcsb.org/structure/8I5Y> | |
|  |  | Lig. Vixotrigine | | | β2 Extracellular (Ig-like) (all beta sheet) | |  | Pub1: [30] | |
| 8S9C (2023) | | Na_v_1.7 | | | β1 Complete (Extracellular + transmembranal) | | EM  3.2 | URL <https://www.rcsb.org/structure/8S9C> | |
|  |  | Lig. Carbamazepine | | | β2 Extracellular (Ig-like) (all beta sheet) | |  | Pub1: [30] | |
| 8I5G (2023) | | Na_v_1.7 | | | β1 Complete (Extracellular + transmembranal) | | EM  2.7 | URL <https://www.rcsb.org/structure/8I5G> | |
|  |  | Lig. PF-05089771 | | | β2 Extracellular (Ig-like) (all beta sheet) | |  | Pub1: [30] | |
| 8I5B (2023) | | Na_v_1.7 | | | β1 Complete (Extracellular + transmembranal) | | EM  2.7 | URL <https://www.rcsb.org/structure/8I5B> | |
|  |  | Lig. Bupivacaine | | | β2 Extracellular (Ig-like) (all beta sheet) | |  | Pub1: [30] | |
| 8I5X (2023) | | Na_v_1.7 | | | β1 Complete (Extracellular + transmembranal) | | EM  2.9 | URL <https://www.rcsb.org/structure/8I5X> | |
|  |  | Lig. Vinpocentine | | | β2 Extracellular (Ig-like) (all beta sheet) | |  | Pub1: [30] | |

**Validation of the generated homology models**

For the validation of the models we submitted a QMEAN study in the Expasy database to verify the positions of the amino acid residues [31,32]. This site allows us to observe if the homology modeling of hNav1.7 is of high quality. This quality is observed with respect to a value between 0 – 1 (0 being the lowest and 1 being the highest). The validation of the homology modeling of the α subunit of hNav1.7 remains within the values between 0.6 - 1 (see Fig. S4).


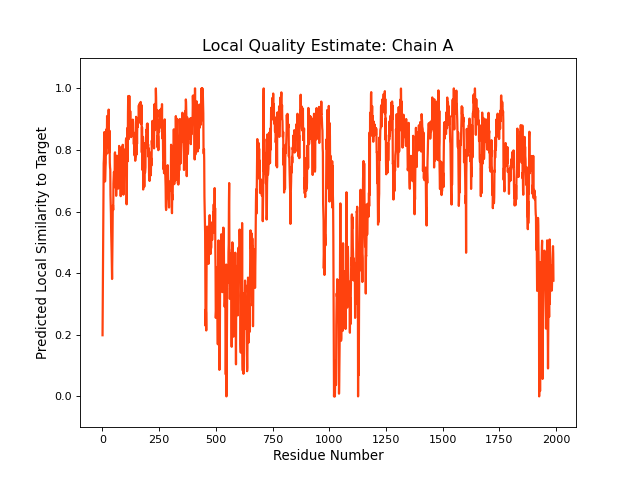


**Fig. S4** Validation of the hNav1.7 α subunit model using the QMEAN value. This allows us to verify the position of the amino acid residues found in the model. When validating it, values between 0.6–1 are generated in the transmembrane areas and less than 0.5 in the intracellular loops.

Similarly, the validation of the β3 subunit is generated by obtaining the QMEAN value (see Fig. S5).


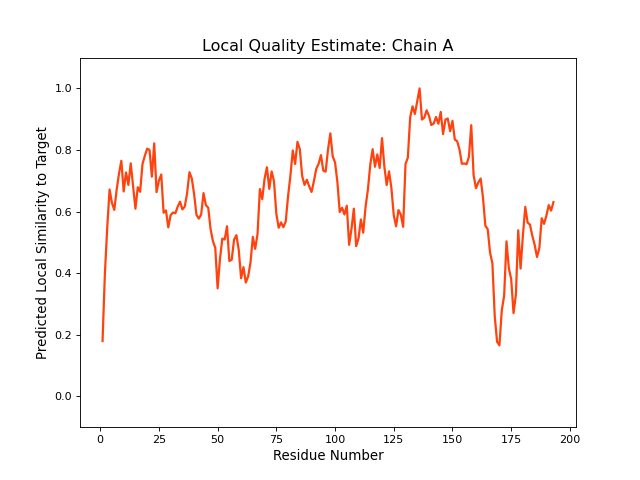


**Fig. S5** Validation of the β3 subunit model using the QMEAN value. Here it can be observed that the values found in the Ig-like domain and in the transmembrane segment present values above 0.6–1. The low values are found in loop areas such as the CDR segments and the C-terminal domain.

Similarly, Ramachandran tables [33,34] of the hNav1.7 α subunit and the β3 subunit were generated in order to observe the structural changes that occur before structural minimization (see Fig. S6 panel (a) and Fig. S7 panel (a)) and after structural minimization (see Fig. S6 panel (b) and Fig. S7 Panel (b)).


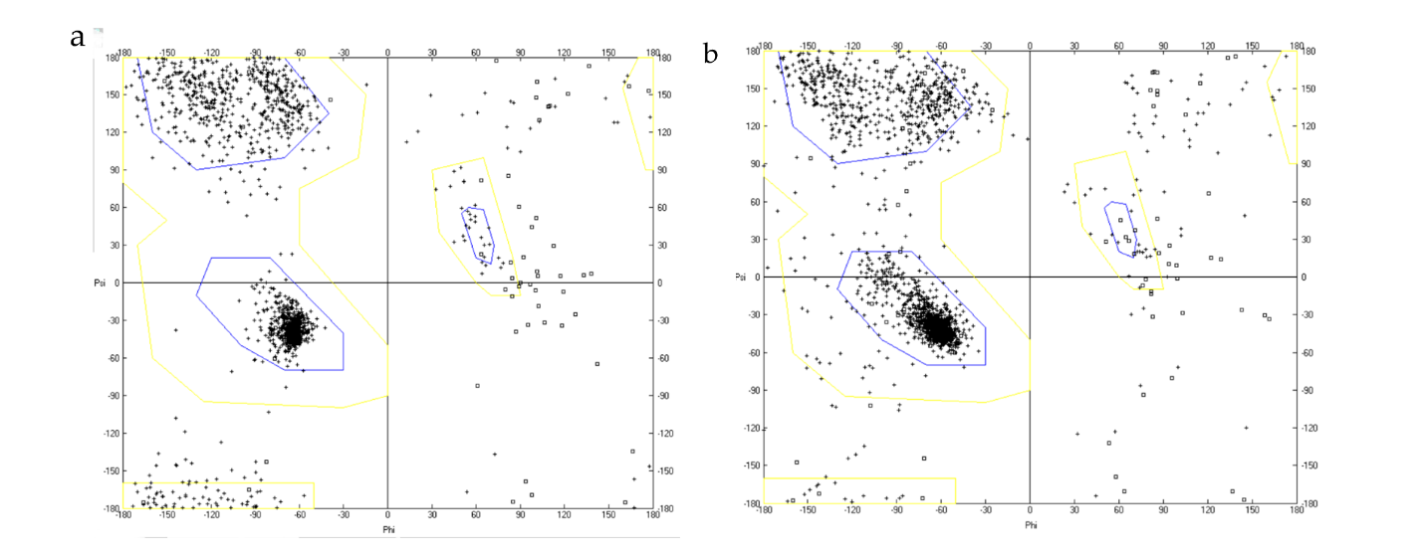


**Fig. S6** Ramachandran plot of the α subunit model. Panel (a) shows the Ramachandran plot before structural minimization showing points that show a series of points that are located in a single site, this refers to the fact that the structure is subject to a process of restriction of movement. Panel (a) shows the Ramachandran plot after structural minimization showing scattered points due to the process of structural relaxation. Of note: the tool for RELAXATION minimization showed scattered points on white background after the process of structural relaxation.


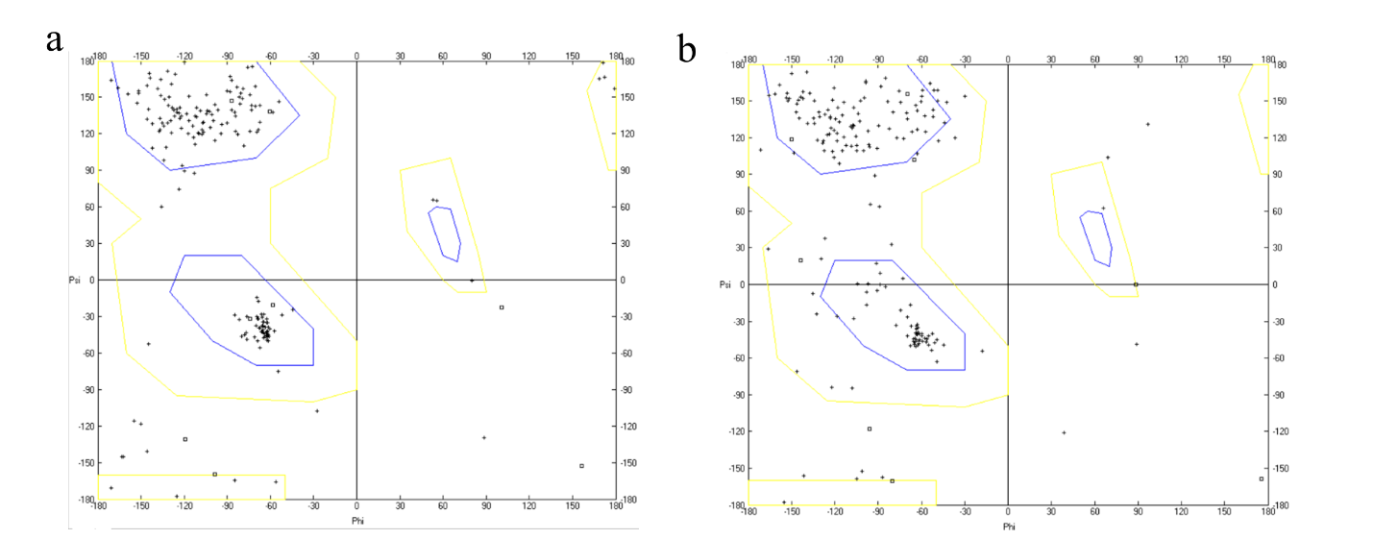


**Fig. S7** Ramachandran plot of the β3 subunit model. Panel (a) shows the Ramachandran plot before structural minimization showing points that show a series of points that are located in a single site, this refers to the fact that the structure is subject to a process of restriction of movement. Panel (b) shows the Ramachandran plot after structural relaxation. Of note, the tool for RELAXATION minimization showed scattered points on white background which can hardly be seen after the process of structural relaxation.

**Part 3: Molecular Docking between α /β3 subunits**

Prior to performing docking simulations, we were searching in the literature the possible interaction zone between both proteins. Zhu and collaborators in 2017 [35] and Barro-Soria and collaborators in 2017 [36] showed effects of the β3 subunit in which it could be positioned in the DIII or DIV. But the strongest interaction energies are found in the DIII domain. All values were assessed by the HDOCK program [37,38]. The strongest free binding energies were taken into account. The large number of results in the area where the resulting position was placed reflected the affinities of the transmembrane as well as the Ig-like portion of the β3 subunit for the α-subunit of hNav1.7 (Fig. S8).


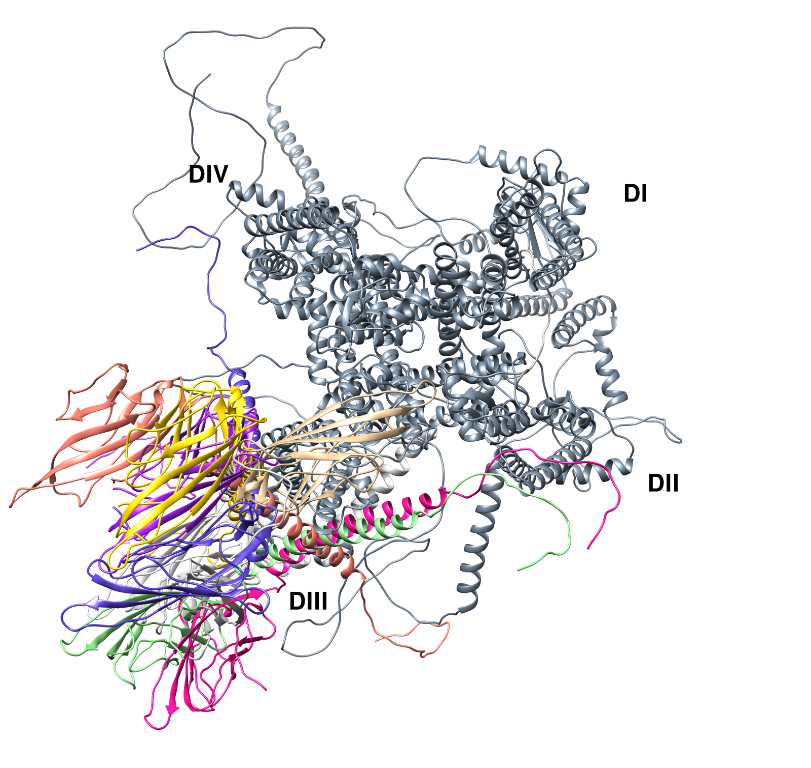


**Fig. S8** Results of the docking simulations between the α subunit and the β3 subunit. The position in brown (tan) is the one that presents the strongest affinity, i.e., highest values of interaction energies. The other docked solutions show bad ligand positions. They are tilted away from the correct position: (i) an 180° rotation with reference to the transmembrane segment as the axis of rotation; (ii) or changing the overall position of the Ig-like into places where it moved away from the α subunit and lose direct contact; (iii) other docking solutions show final poses in which the β3 subunit is placed below, i.e., with the Ig-like segment in the area that would lie in the intracellular area.

Molecular docking by HDOCK [37,38] gives us docking score of -231.26; with the docking value you get a confidence score of 0.83 in the DIII domain of hNav1.7 (Fig. S9). to verify our docking, we perform back docking using the β1 subunit and the DIII domain of hNav1.7 giving us a docking score of -240.53 and a confidence score of 0.86 (Fig. S13) with a RMSD 0.6 Å compared with Cryo-EM (PDB code 6J8I). The amino acid residues that allow the interaction between β3 subunit for the transmembrane part are as follows: T154, S158, L165, L166, L169, W172, E176, M177, E180, Y181, V184 and E186; for DIII domain are: I1177, N1180, I1181, K1183, T1184, K1187, I1188, I1191, W1193, F1194, F1197, E1217, R1218, K1220, T1221, I1224, I1225, Y1228, I1232, I1236.


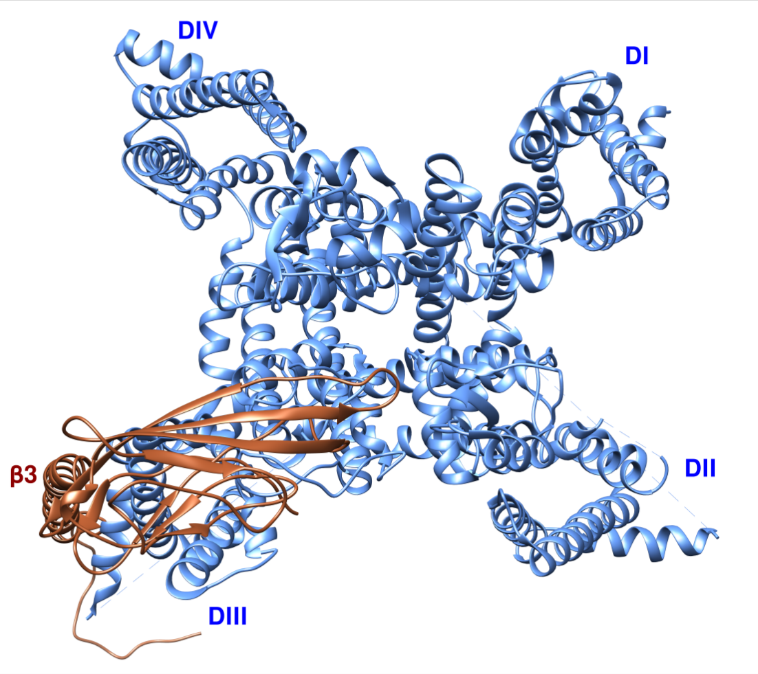


**Fig. S9** Final docked pose of ligand (β3, brown ribbon) against receptor (α, blue ribbon). The computed interaction site between β3 and α was confirmed with experimentally determined complexes (PDB codes: 7TJ8 of Nax, and 6J8i of hNav1.7). The subunit ligand was a strong binder at the (correct) α/β3 interface and yielded the best confidence score 0.86 among all computed (wrong) poses.

**The majority of interactions were identified and summarized in three groups (i of iii):**

(i) E32–K1220 forming a salt bridge which is located at 5 Å (see Fig. S7).


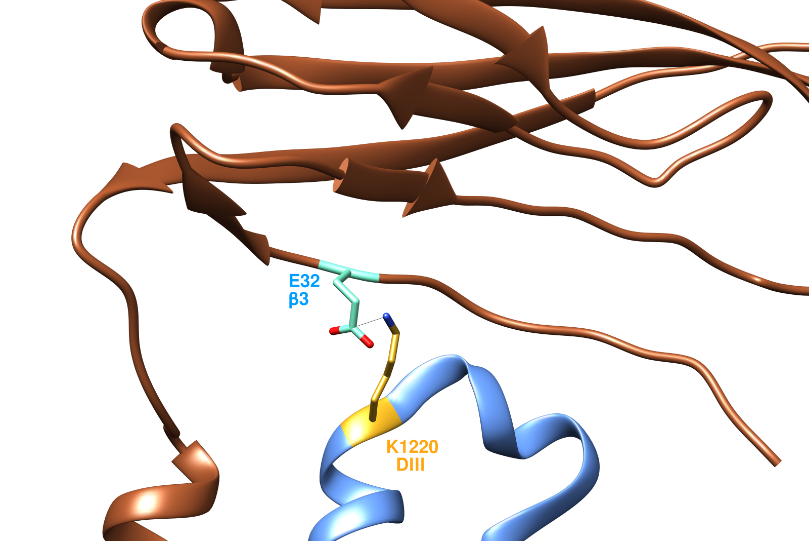


**Fig. S10** Interaction between E32 of the β3 subunit with the K1220 residue of the DIII domain. This interaction presents a salt bridge and is approx. 5 Å long.

The majority of interactions were identified and summarized in three groups (ii):

(ii) The interaction between E159 (grey)–Y1228 (magenta) by the formation of a hydrogen bond that was located at a distance less than 3 Å (see Fig. S11**)**.


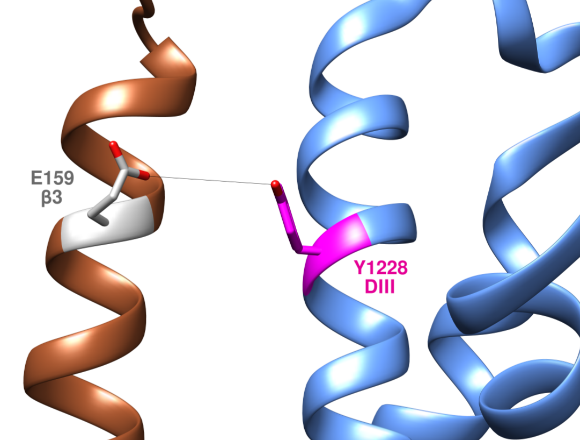


**Fig. S11** Interaction of the amino acidic residue E159 (-COO group) of the β3 subunit with residue Y1228 (-OH group) of the DIII domain. That interaction would allow the formation of a hydrogen bond.

The majority of interactions were identified and summarized in three groups (iii):

(iii) A hydrophobic pocket formed between W172 + F1194 + F1197, causing W172 to interact with phenylalanine residues (see Fig. S12). Interaction zones between the transmembrane segment and the DIII domain of hNav1.7 (β3 subunit W172 (grey) and VSD F1197 (salmon) and F1194 (pink)). Hydrophobic pocket formed between W172 + F1194 + F1197 that presents a π-π electron attraction.


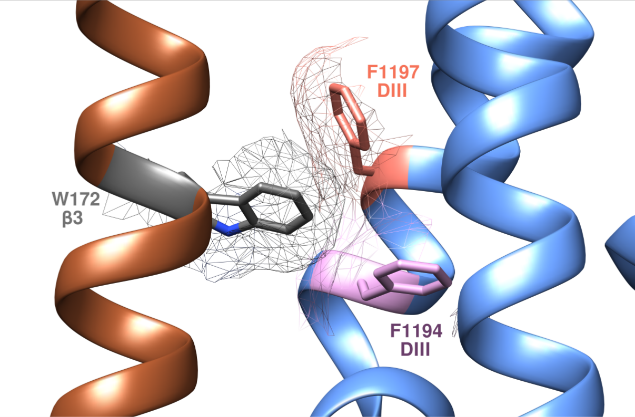


**Fig. S12** Interaction between residue W172 of the β3 subunit with aromatic residues F1194 and F1197 of the DIII domain. The interaction here would give the formation of a hydrophobic pocket with π electron-rich region between three aromatic side chains (W172, F1194 and F1197).


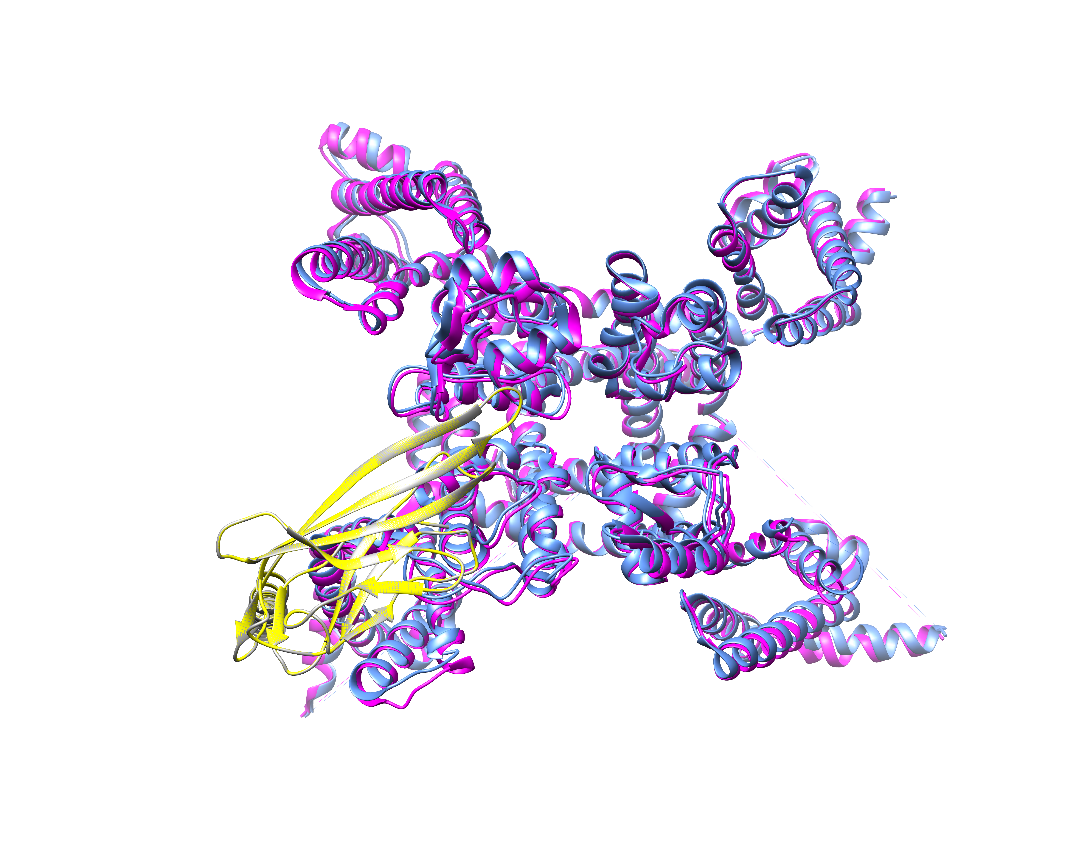


**Fig. S13** Final position of the back docking in HDOCK with the ligand (β1 subunit, grey representing a ribbon) and the receptor (α subunit, blue representing a ribbon). The interaction when compared with its crystal (magenta receptor and yellow ligand) shows an RMSD of 0.6 Å.

In HADDOCK 2.4 [39,40] the binding site is obtained in the DIII domain of hNav1.7 with a docking value of 39.5. The β1 subunit positions the transmembrane segment in the same position as the crystal but the Ig-like domain is oriented towards another position. The orientation that the Ig-like domain takes is towards the S5 and S6 segments of the DIV domain (Fig. S14). When compared with the crystal, we get an RMSD of 5 Å.


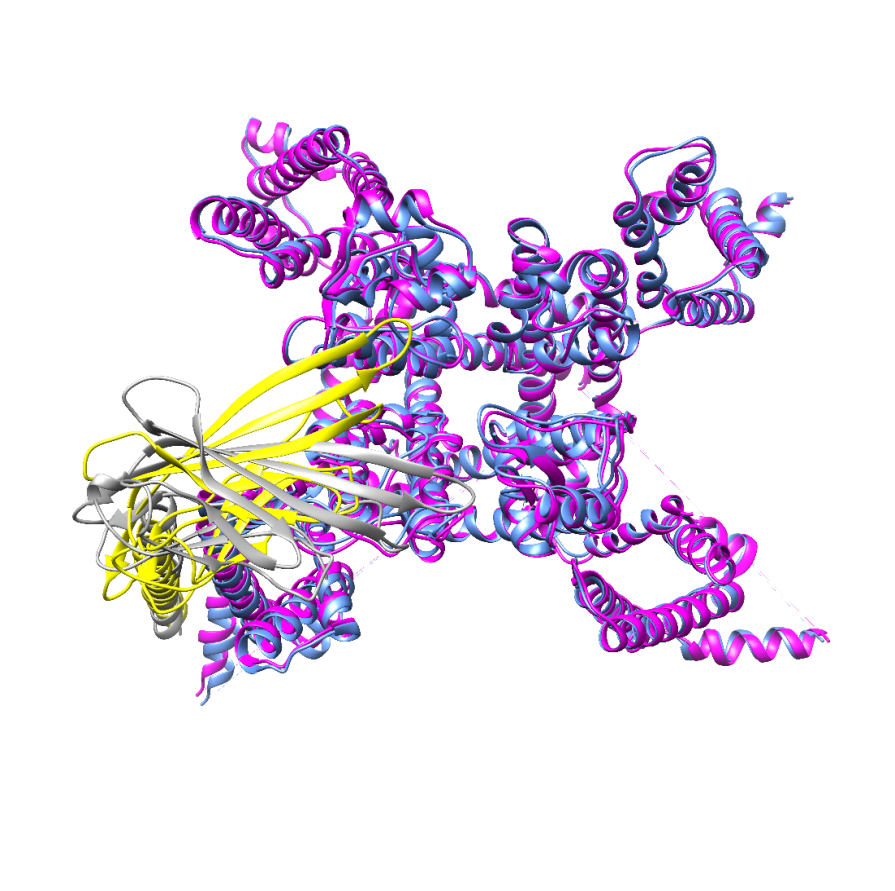


**Fig. S14** Final position of the back docking in HADDOCK 2.4 with the ligand (β1 subunit, grey ribbon) and the receptor (α subunit, blue ribbon). The interaction when compared with its crystal (magenta receptor and yellow ligand) shows an RMSD of 5 Å.

**Part 4: Molecular Dynamics of 3D Models**

The system to be used to generate molecular dynamics consists of the complete sodium channel α subunit, the β3 subunit (which is coupled to the VSD_DIII_ domain), a lipid membrane consisting of the POPC lipid model, water in the TIP3P model and NaCl at a concentration of 0.15M (Fig. S15).


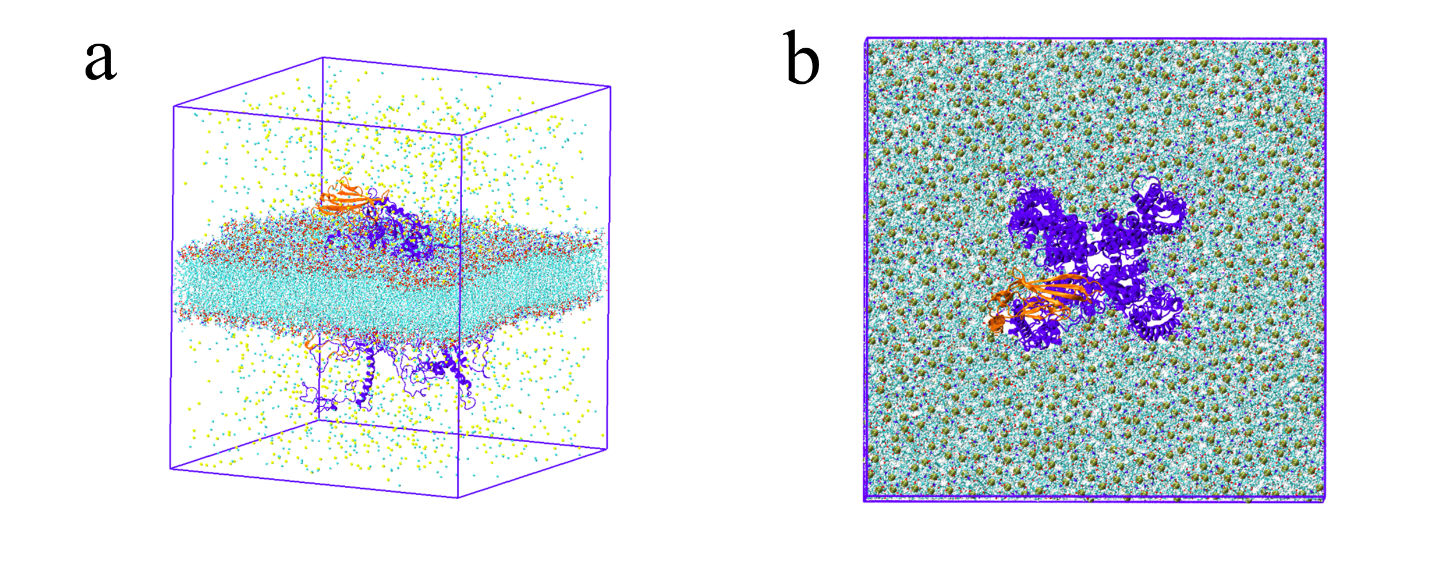


**Fig. S15** Representation of the system that was simulated and for which the results are being displayed. a) The system consists of α subunit with a blue ribbon representation, β3subunit with a sienna colour ribbon representation. The lipid membrane is represented by the sky blue chains and the red dots representing the POPC oxygens. Likewise, the Na+ ion is coloured with yellow dots and the chloride ion with blue dots. Water is omitted for a better visualisation of the system. b) Top view of the system showing in the centre the simulated complex consisting of the α subunit and the β3 subunit in blue and sienna. The spheres shown are the phosphorus atom forming part of the phosphate group of the POPC lipid.

The distance measurements could be brought on the same footing as they revealed similar positional pattern – if not the same molecular behaviour of residues at the PPI. At the beginning of MD production runs the side chains on one protein (c) did not have the opportunity to interact with the side chains on the other protein (β3). Hence, all diagrams showed longer distances in the first few nanoseconds. Upon mutual side chain rearrangements at the PPI the distances became shorter ranging between 10 and 5 Ångstroms, all of which reflected an overall favourable attraction at the interface between α and β3. The side chains reoriented from a greater distance to recognize their respective counter residues in search of favourable interactions in a few nanoseconds (neighbourhood effects). Thereafter, the side chain geometries remained either unchanged or fluctuated around a stable conformation during 50 ns or more. After that time conformational leaps took place given the kinetic energy in the surrounding thermal bath. Again, the side chains would reposition and optimize mutually their (interaction) positions. In terms of MD, it constitutes a repeated optimization process to lower (mechanical) strain energy by reducing bad contacts. On the other hand, the fact that distances did not became larger is discussed here as a strong proof of concept. If the computed 3D model had not had a correct PPI, the unfavourable neighbourhood effects by missing side chain complementarity would have led to complex instabilities. And over the observation window of reported 300 nanoseconds both proteins would have separated in space.


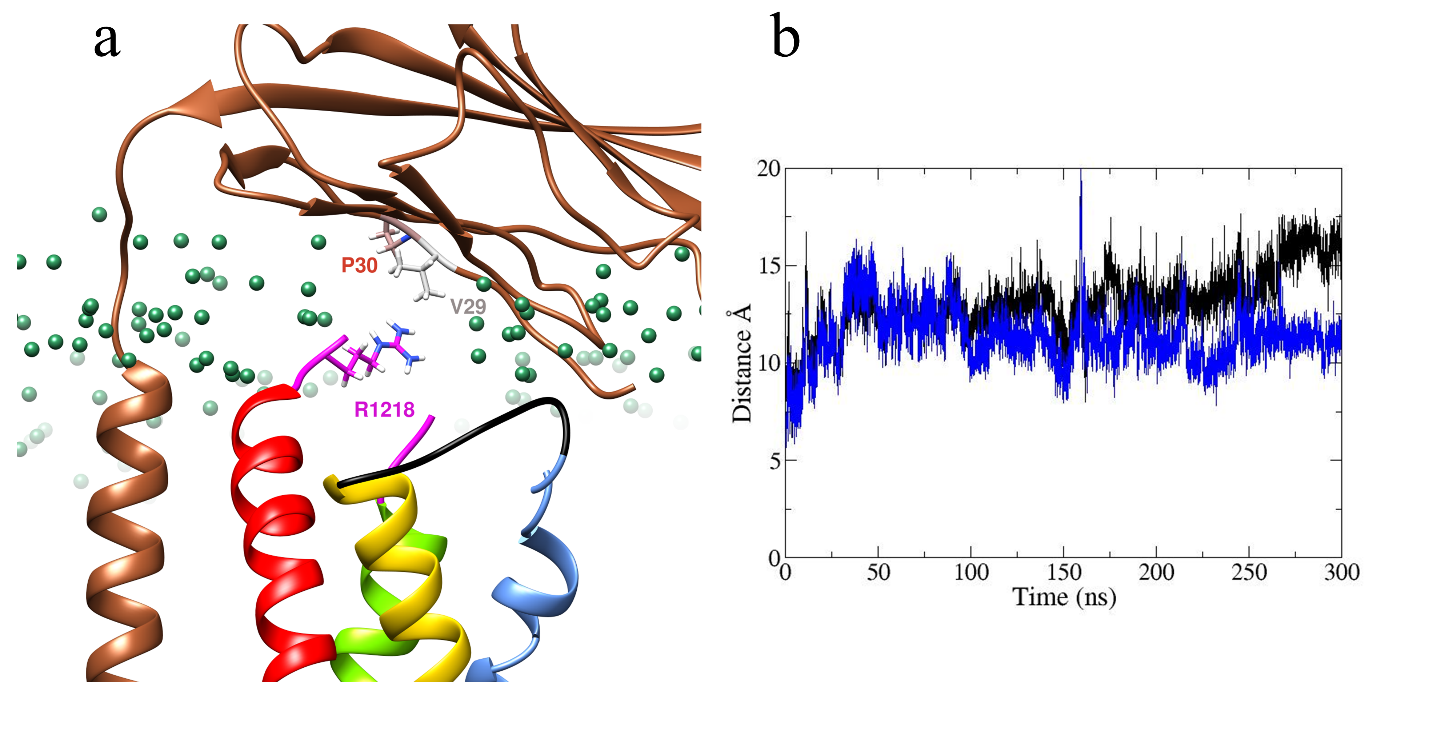


**Fig. S16** Graphic representation of the interaction of V29, P30 with R1218. (a) The van der Waals interaction is carried out by the side chain of R1218 with the side chains of V29, P30. Colour coding: β3 subunit in sienna, segment S1, S2, S3, S4 in chartreuse, red, yellow and cornflower blue, respectively. The phosphate group of the POPC lipid is represented by the spheres in green. (b) Due to the loop movement, the distance between these residues becomes an interaction distance from 5Å to 10Å; The black line shows the distance between V29-R1218 and the purple line shows the distance between P30-R1218. The lower distance on y-axis is under 6 Å at on x-axis 5–10 ns.


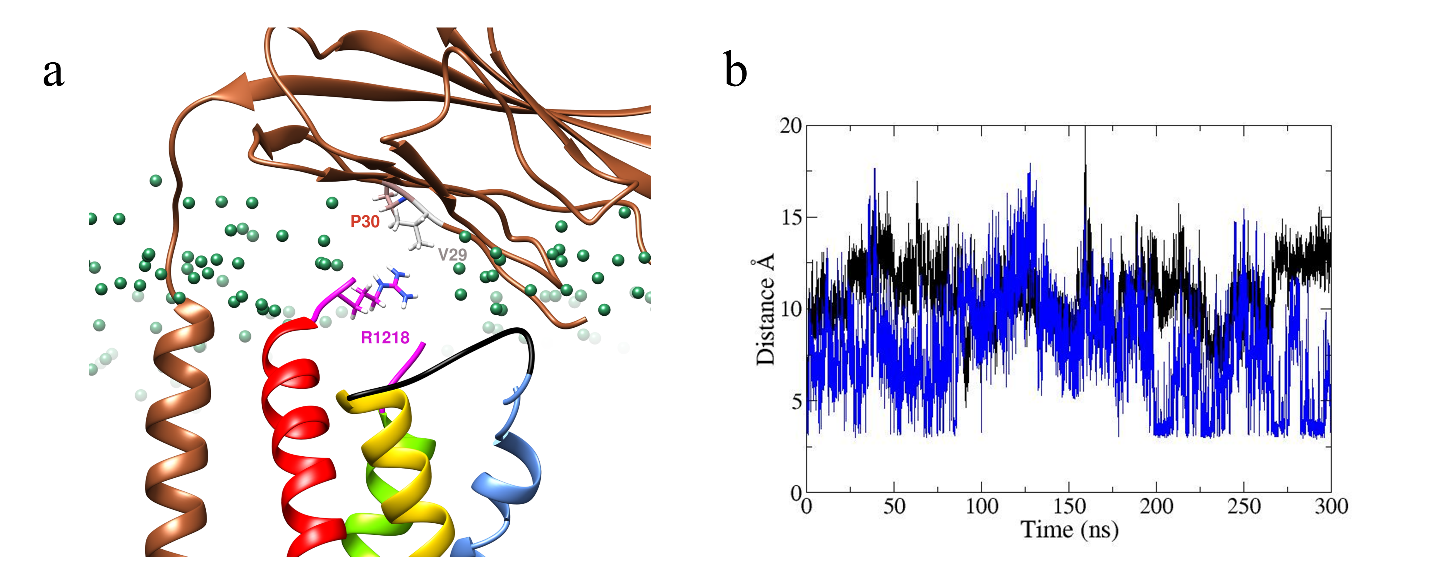


**Fig. S17** Graphic representation of the interaction between P30–K1220, E32 with K1220. (a) The interaction between P30 and K1220 only occurs at the beginning of molecular dynamics. However, the loop movement quickly brakes up the noncovalent bonding. The interaction between E32–K1220 occurs for some moments forming a salt bridge and subsequent to the formation of electrostatic interaction. Colour coding: β3 subunit in sienna, segment S1, S2, S3, S4 in chartreuse, red, yellow and cornflower blue, respectively. The phosphate group of the POPC lipid is represented by the spheres in green. (b) The interaction between P30–K1220 (black) and E32–K1220 (violet) breaks the interaction because the distance increases and between E32–K1220 that present the salt bridge breaks and only electrostatic interactions occur during most of the time. The lower distance on y-axis is under 4 Å at on x-axis 10 ns.


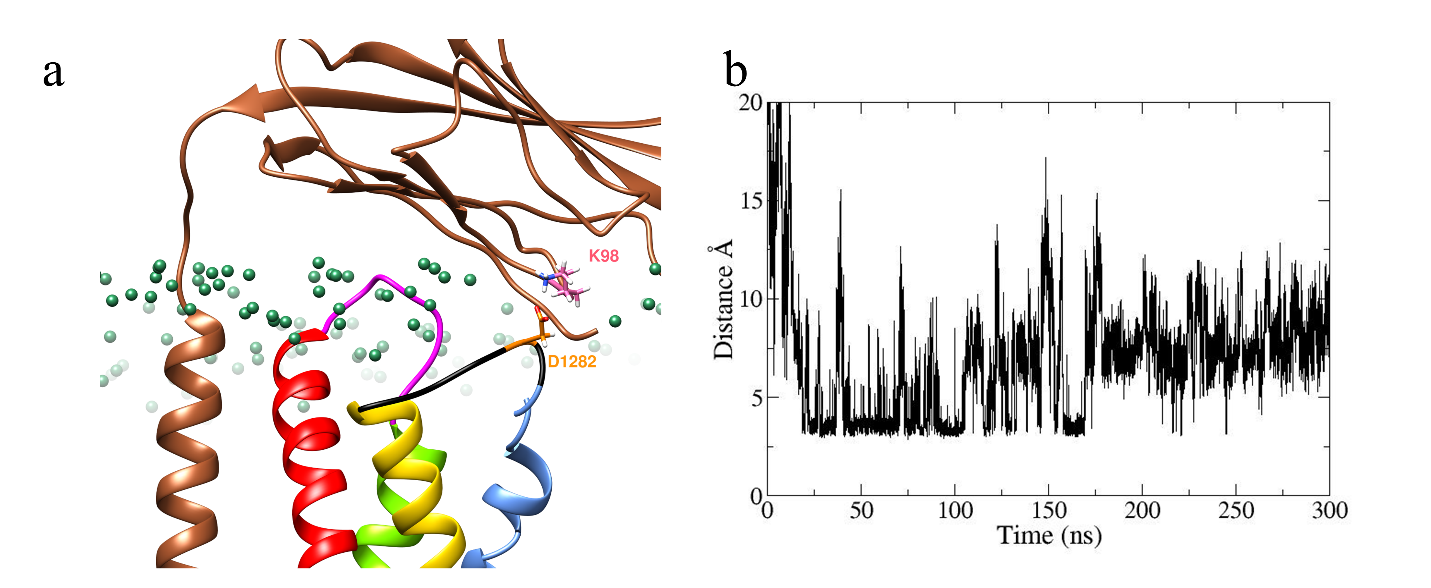


**Fig. S18** Graphic representation of the interaction between K98 and D1282. (a) A salt bridge has been established thanks to favourable electrostatic attractions. Colour coding: β3 subunit in sienna, segment S1, S2, S3, S4 in chartreuse, red, yellow and cornflower blue, respectively. The phosphate group of the POPC lipid is represented by the spheres in green. (b) Initially the interatomic distance fluctuation drops and the position of K98 on CDR2 favours a salt bridge formation between 20 ns to 160 ns. When the salt bridge is lost mere electrostatic interactions occur at short distance ranges. The lower distance on y-axis is under 3 Å at on x-axis 20 ns.


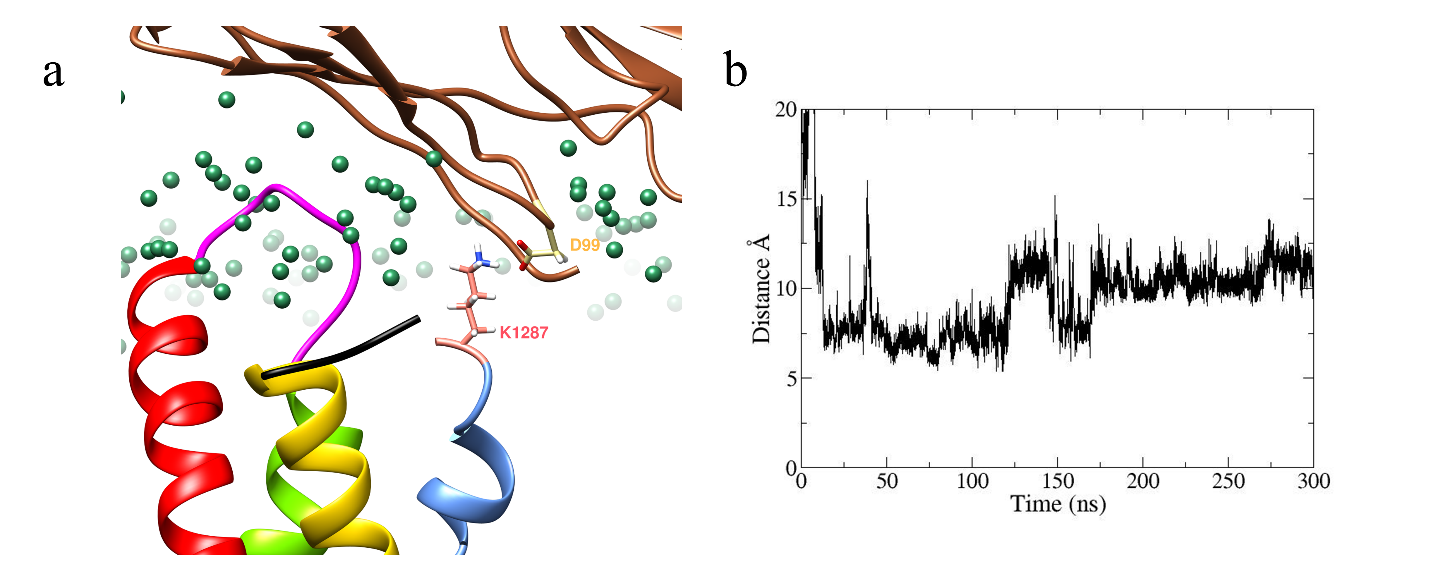


**Fig. S19** Graphic representation of the interaction between D99 of the β3 subunit with K1287. (a) During the first nanoseconds a salt bridge can be established but it is lost at a later time (150 ns). So, the closer vicinity between D99 and K1287 is lost (cf reference value 4 on the y axis). After 150 ns electrostatic forces still keep the residues in close range (cf. values between 8 and 10 on the y axis). Colour coding: β3 subunit in sienna, segment S1, S2, S3, S4 in chartreuse, red, yellow and cornflower blue, respectively. The phosphate group of the POPC lipid is represented by the spheres in green. (b) These distance changes from 3.5–8Å interaction are due to D99 being located in CDR2. The lower distance on y-axis is under 3 Å at on x-axis 15 ns.


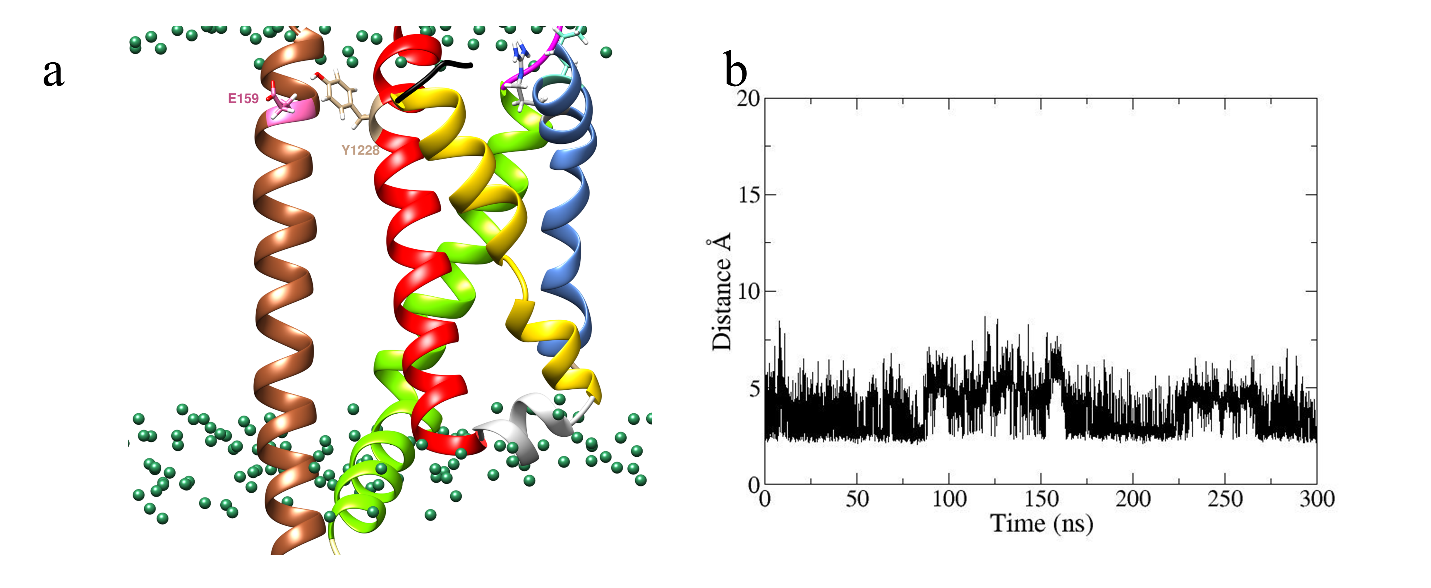


**Fig. S20** Graphic representation of the interaction between E159 and Y1228. (a) Here a hydrogen bond is formed. Electrostatic interactions provide stability at the surface of the transmembrane segment. Colour coding: β3 subunit in sienna, segment S1, S2, S3, S4 in chartreuse, red, yellow and cornflower blue, respectively. The phosphate group of the POPC lipid is represented by the spheres in green. (b) The hydrogen bond exists in a time window from 0 to 80 ns and after 160 to 230 ns. The lower distance on y-axis is under 2.3 Å at on x-axis 1 ns.


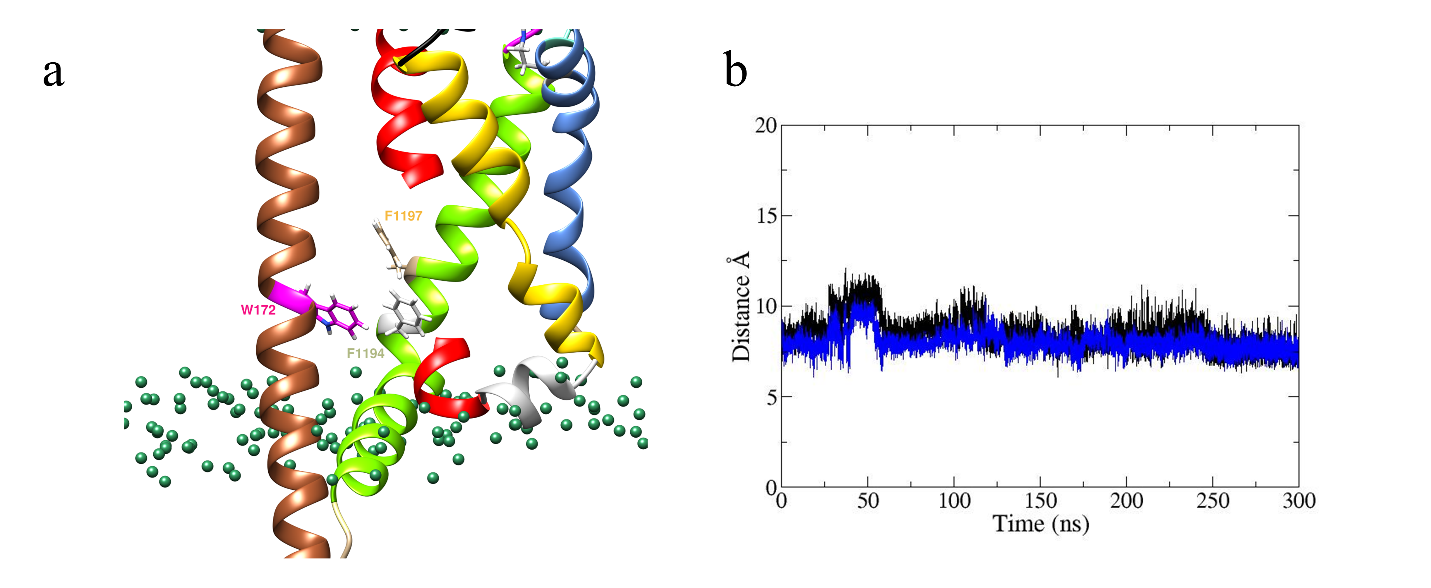


**Fig. S21** Graphic representation of the interaction between W172–F1194–F1197. (a) This interaction occurs in a hydrophobic pocket that presents a π-π parallel display type interaction and a π-π stacking type interaction. Colour coding: β3 subunit in sienna, segment S1, S2, S3, S4 in chartreuse, red, yellow and cornflower blue, respectively. The phosphate group of the POPC lipid is represented by the spheres in green. (b) They contact each other during most of the simulation time. The fluctuations of the interaction are generated when the side chain of the amino acids rotates to be in contact with the membrane lipids. The lower distance on y-axis is under 6 Å at on x-axis 20 ns.


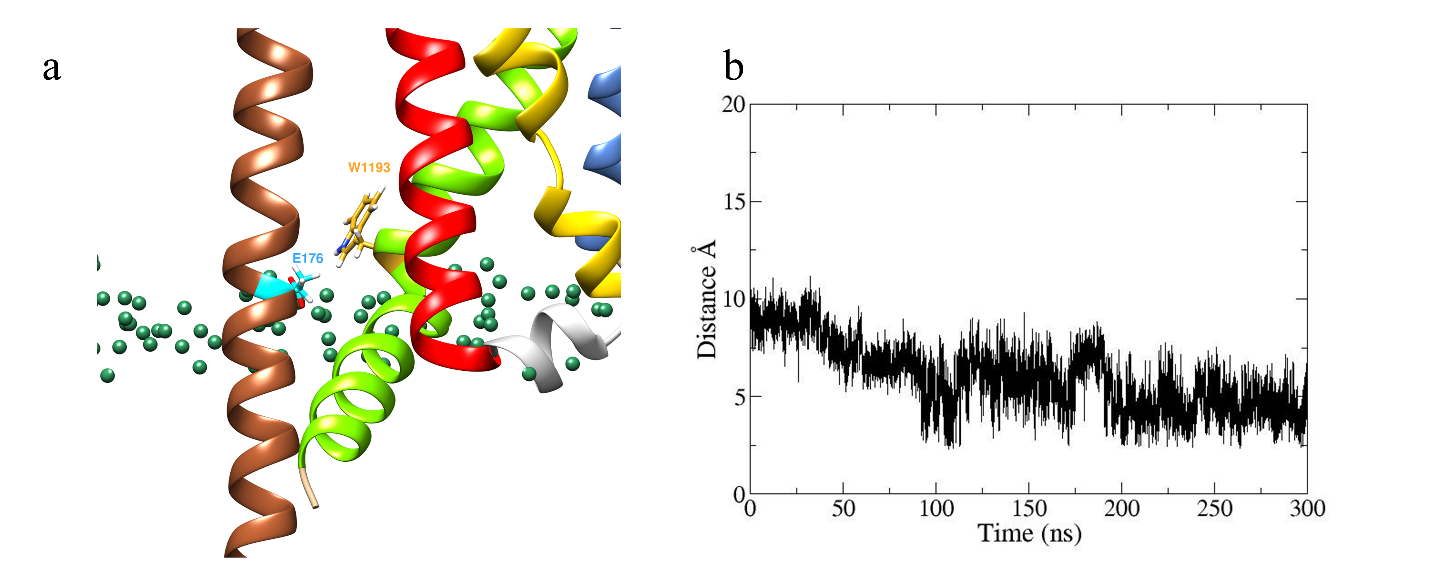


**Fig. S22** Graphic representation of the interaction between E176–W1193. (a) This interaction is due to the position that the side chains take and allows the carbonyl group of E176 and the side chain of W1193 to orient and form at some moments the hydrogen bond. Colour coding: β3 subunit in sienna, segment S1, S2, S3, S4 in chartreuse, red, yellow and cornflower blue, respectively. The phosphate group of the POPC lipid is represented by the spheres in green. (b) This interaction occurs after 80 ns, which there presents the formation of the hydrogen bond and due to the position of W1193, its side chain rotates and increases the distance, causing them to interact through electrostatic forces. The lower distance on y-axis is under 2.5 Å at on x-axis 95 ns.


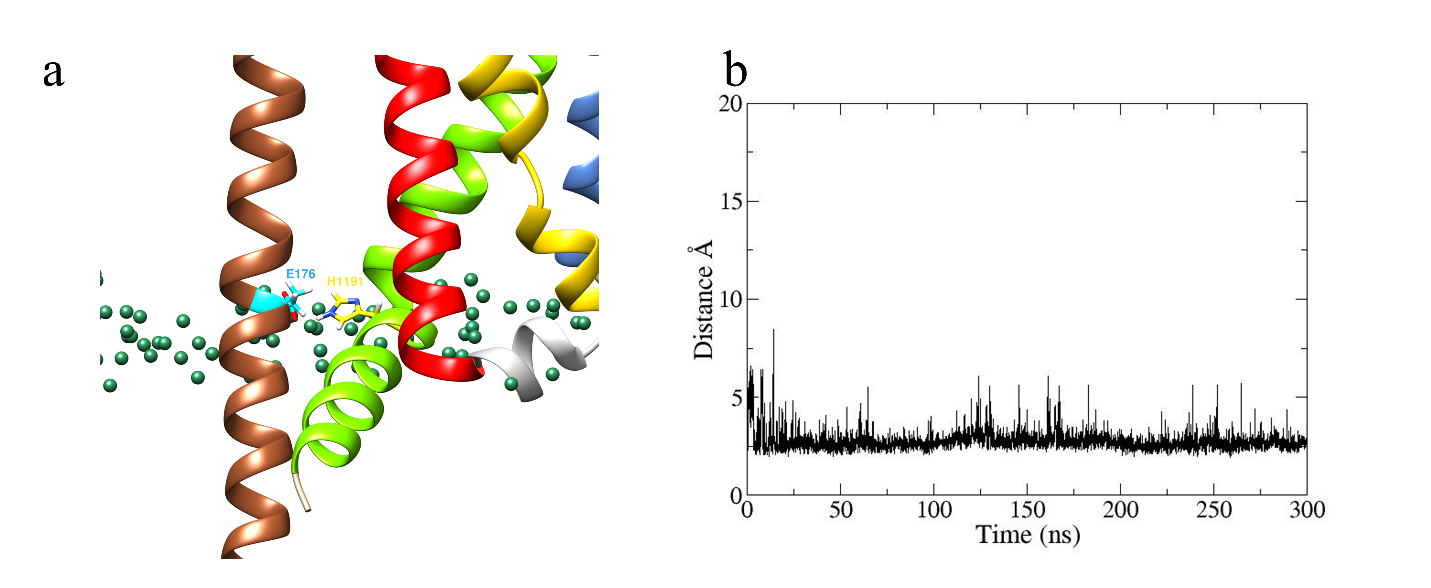


**Fig. S23** Representation of the interaction between E176 and H1191. (a) Interaction between E176 (cyan)-H1191 (yellow) at 10ns; Figure made with chimera. Colour coding: β3 subunit in sienna, segment S1, S2, S3, S4 in chartreuse, red, yellow and cornflower blue, respectively. The phosphate group of the POPC lipid is represented by the spheres in green. (b) Graph of the distance in the formation of a hydrogen bond between E176 and H1191 the α subunit of the sodium channel hNav1.7 during the 100 ns. The lower distance on y-axis is under 2.3 Å at on x-axis 5 ns.


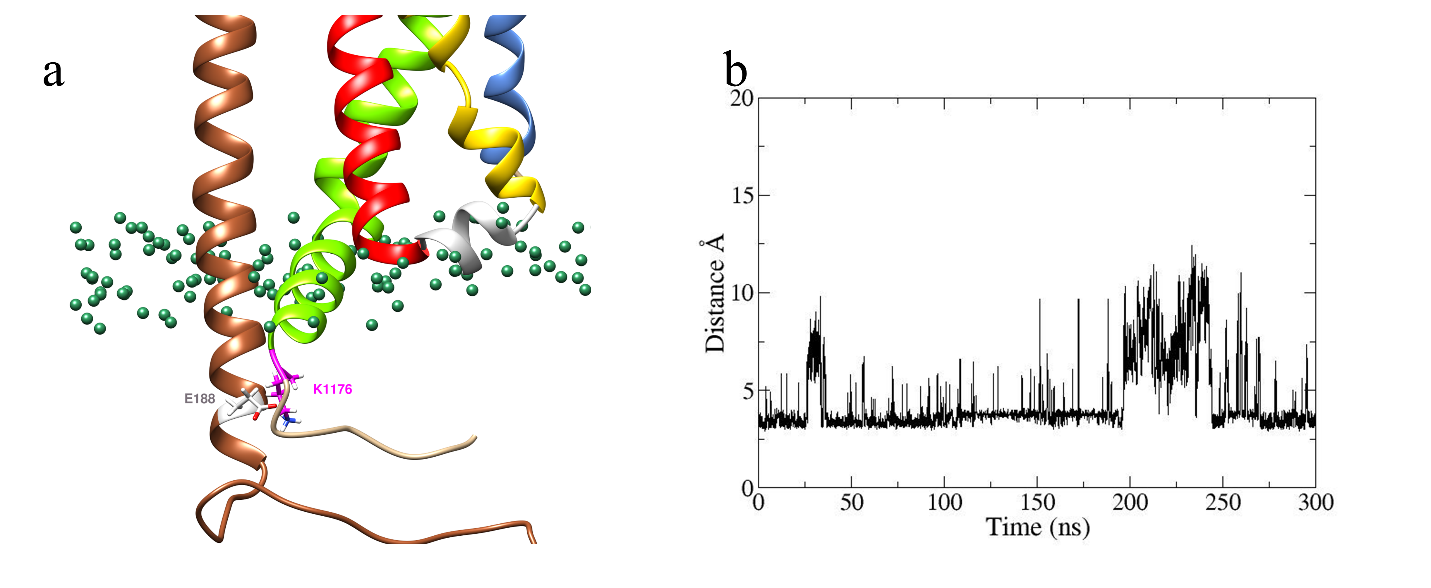


**Fig. S24** Representation of the interaction between E188 and K1176. (a) Interaction between E188 (gray)-K1176 (pink) at 10ns; Figure made with chimera. Colour coding: β3 subunit in sienna, segment S1, S2, S3, S4 in chartreuse, red, yellow and cornflower blue, respectively. The phosphate group of the POPC lipid is represented by the spheres in green. (b) Graph of the distance in the formation of a hydrogen bond between E188 and K1176 the α subunit of the sodium channel hNav1.7 during the 100 ns. The lower distance on y-axis is under 3.5 Å at on x-axis 2 ns.


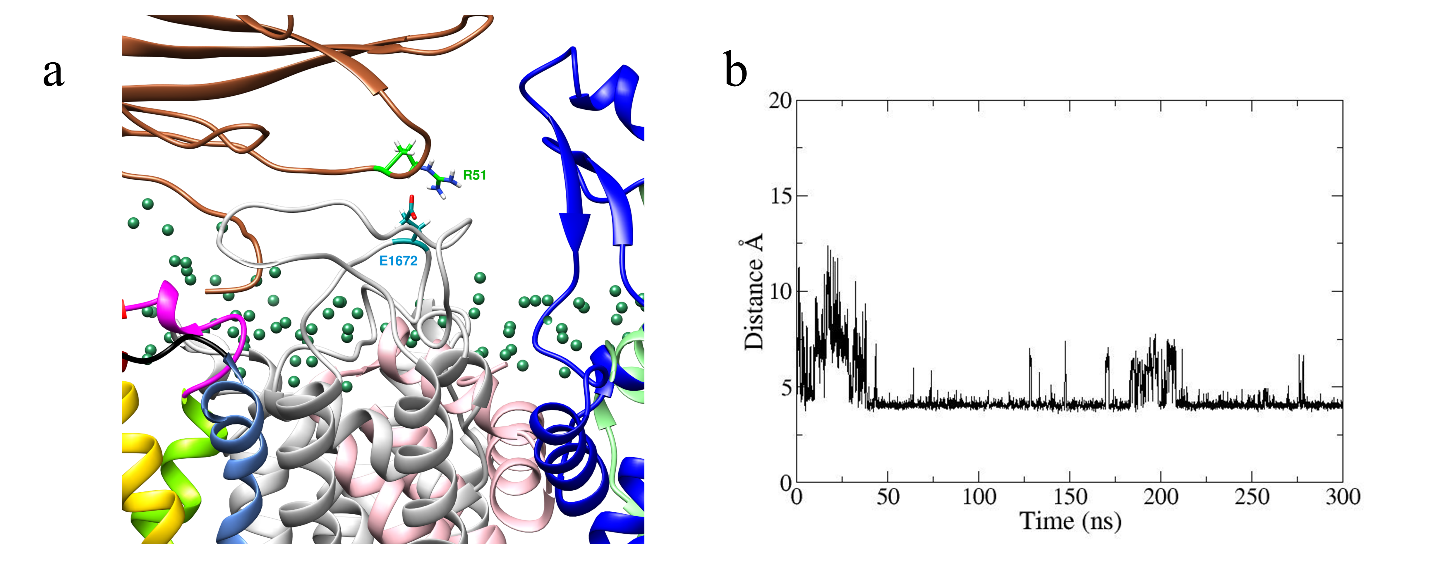


**Fig. S25** Representation of the interaction between R51 - E1672. (a) Interaction between R51 (green)–E1672 (light blue) at 10ns; Figure made with chimera. (b) Plot of the distance in the formation of a salt bridge between R51 and E1672 the α subunit of the sodium channel hNav1.7 during the 100 ns. The lower distance on y-axis is under 5 Å at on x-axis 10 ns. Representation of the interaction between R51-E1672. (a) Interaction between R51 (green)–E1672 (light blue) at 10ns; Figure made with chimera. Colour coding: β3 subunit in sienna, segment S1, S2, S3, S4 in chartreuse, red, yellow and cornflower blue, respectively. The phosphate group of the POPC lipid is represented by the spheres in green (b) Plot of the distance in the formation of a salt bridge between R51 and E1672 the α subunit of the sodium channel hNav1.7 during the 100 ns. The lower distance on y-axis is under 5 Å at on x-axis 10 ns.


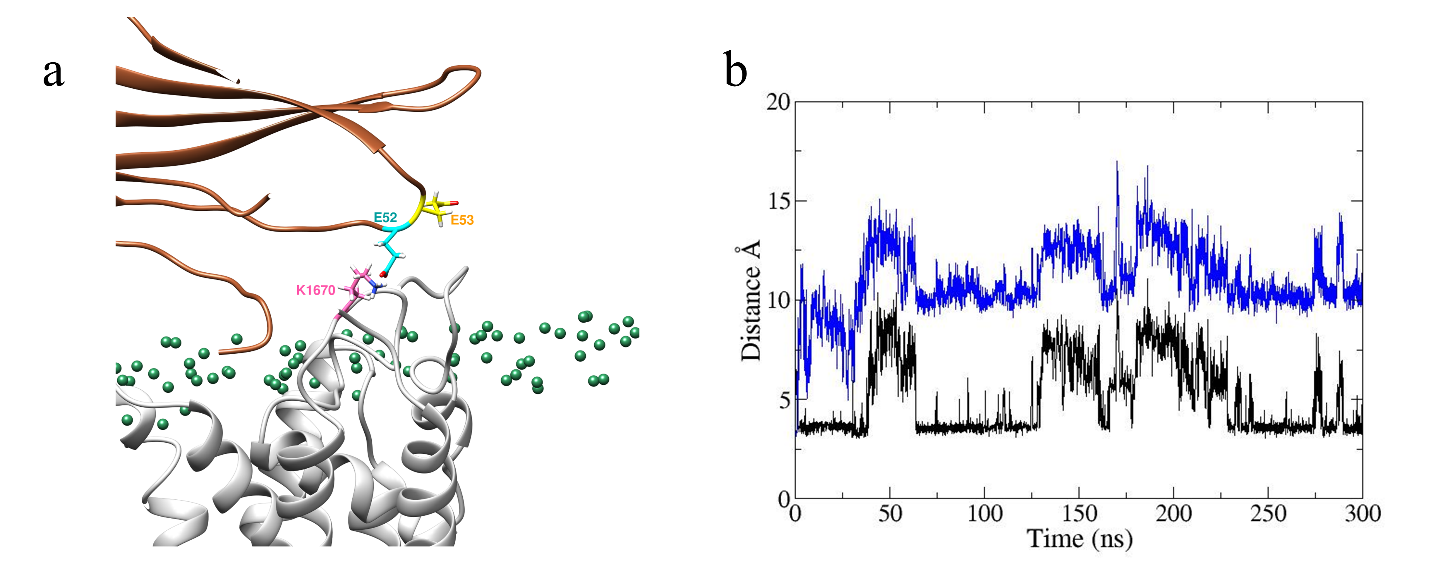


**Fig. S26** Representation of the interaction between E52/E53-K1670. (a) Interaction between E52 (blue)–K1670 (pink) and E53 (yellow)–K1670 (pink) at 10ns; Figure made with chimera. Colour coding: β3 subunit in sienna, segment S1, S2, S3, S4 in chartreuse, red, yellow and cornflower blue, respectively. The phosphate group of the POPC lipid is represented by the spheres in green. (b) Plot of the distance in the formation of a salt bridge between R51 and E1672 the α subunit of the sodium channel hNav1.7 during the 100 ns. The lower distance on y-axis is under 4 Å at on x-axis 75 ns for E52–K1670 interaction.


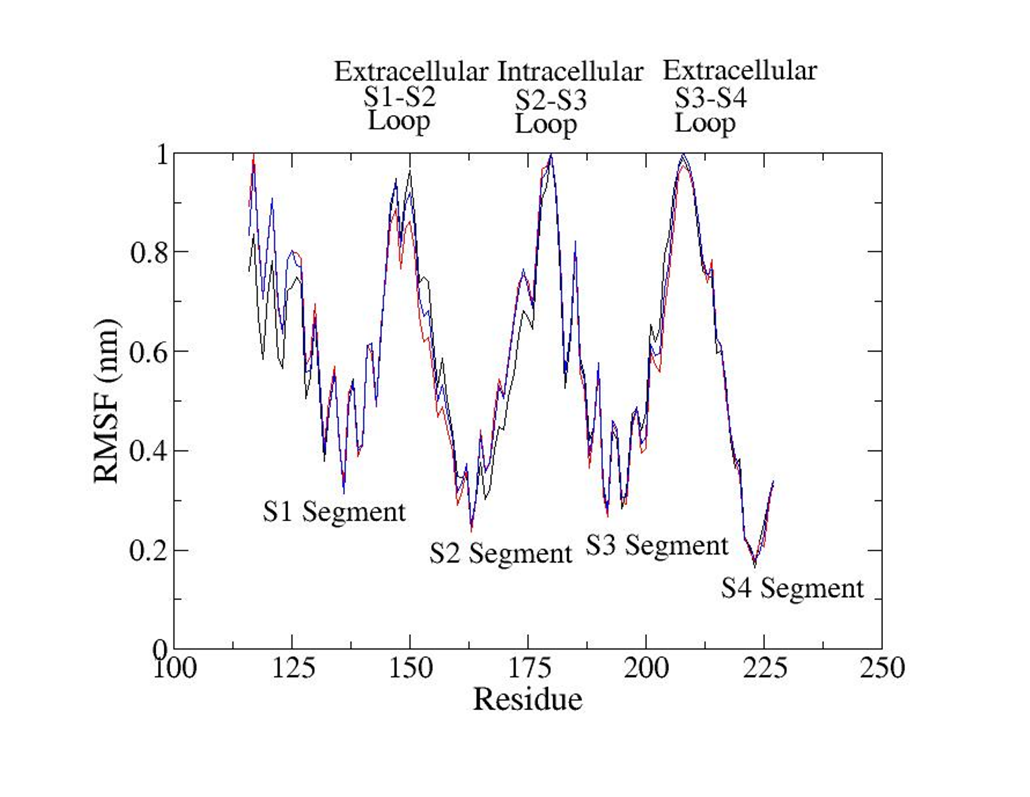


**Fig. S27** RMSF plot for VSD_DI_ domain of the α subunit over the 300 ns of registered productive run time. The high mobility of the amino acid residues shows the elevation on the RMS: the first peak is formed with amino acids from S1-S2 extracellular loop. The latter embraced amino acids 173 to 185. The second peak was composed of amino acids from the S2-S3 intracellular loop with residues 205 to 210. The lines show the movement of the structures through the 300 ns; Black line = 0 – 100 ns. Red line = 100 – 200 ns. Blue line = 200 - 300 ns.


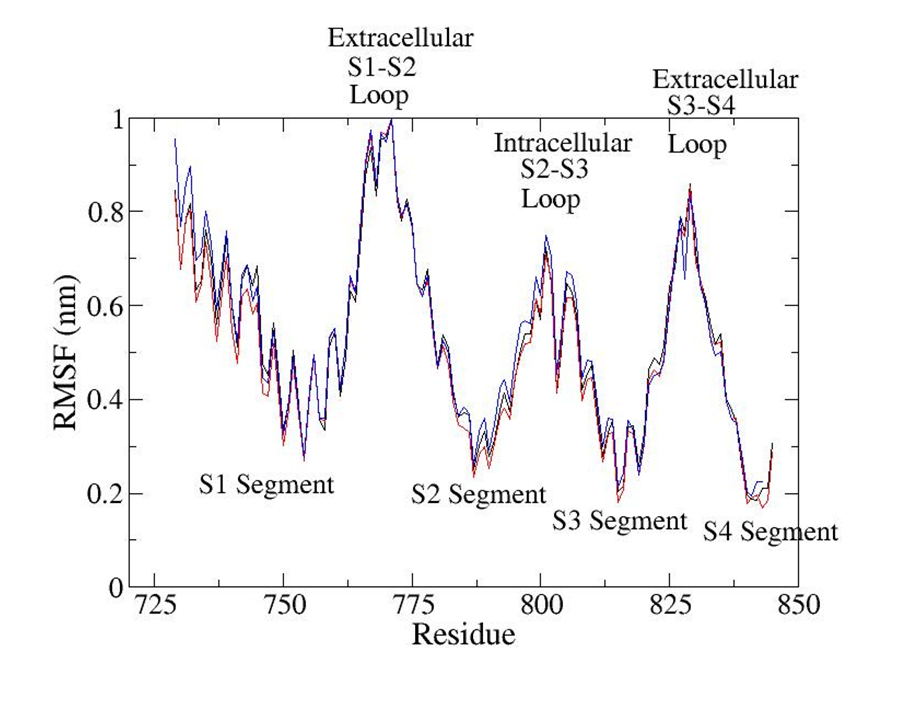


**Fig. S28.** RMSF plot for VSD_DII_ domain of the α subunit over the 300 ns of registered productive run time. The high mobility of the amino acid residues shows the elevation on the RMS: the first peak is formed with amino acids from S1-S2 extracellular loop. The latter embraced amino acids 763 to 771. The second peak was composed of amino acids from the S2-S3 intracellular loop with residues 797 to 805. The lines show the movement of the structures through the 300 ns; Black line = 0 – 100 ns. Red line = 100 – 200 ns. Blue line = 200 - 300 ns.


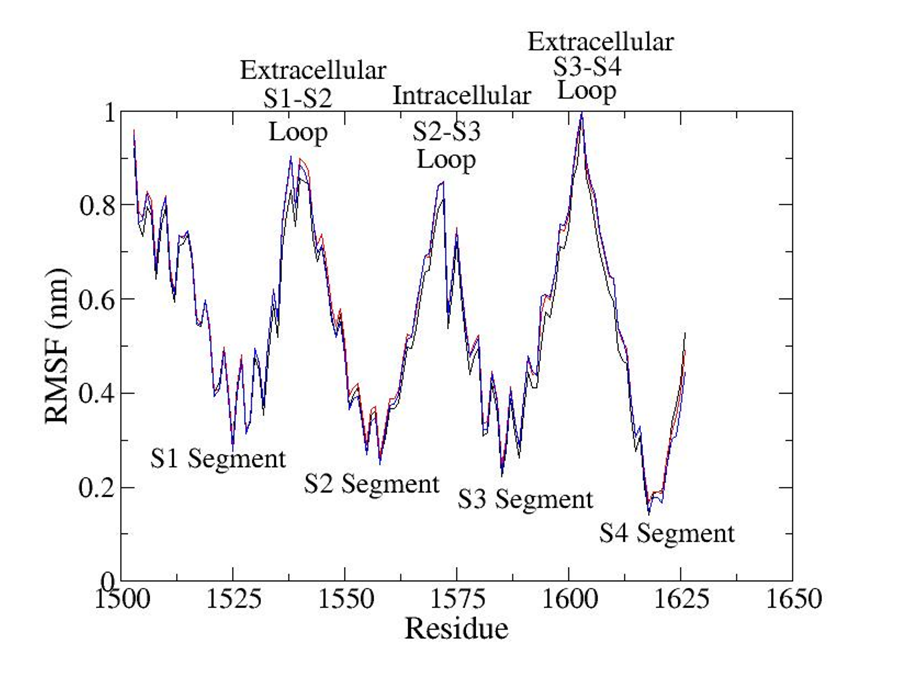


**Fig. S29** RMSF plot for VSD_DIV_ domain of the α subunit over the 300 ns of registered productive run time. The high mobility of the amino acid residues shows the elevation on the RMS: the first peak is formed with amino acids from S1-S2 extracellular loop. The latter embraced amino acids 1458 to 1514. The second peak was composed of amino acids from the S2-S3 intracellular loop with residues 1535 to 1545. The lines show the movement of the structures through the 300 ns; Black line = 0 – 100 ns. Red line = 100 – 200 ns. Blue line = 200 - 300 ns.


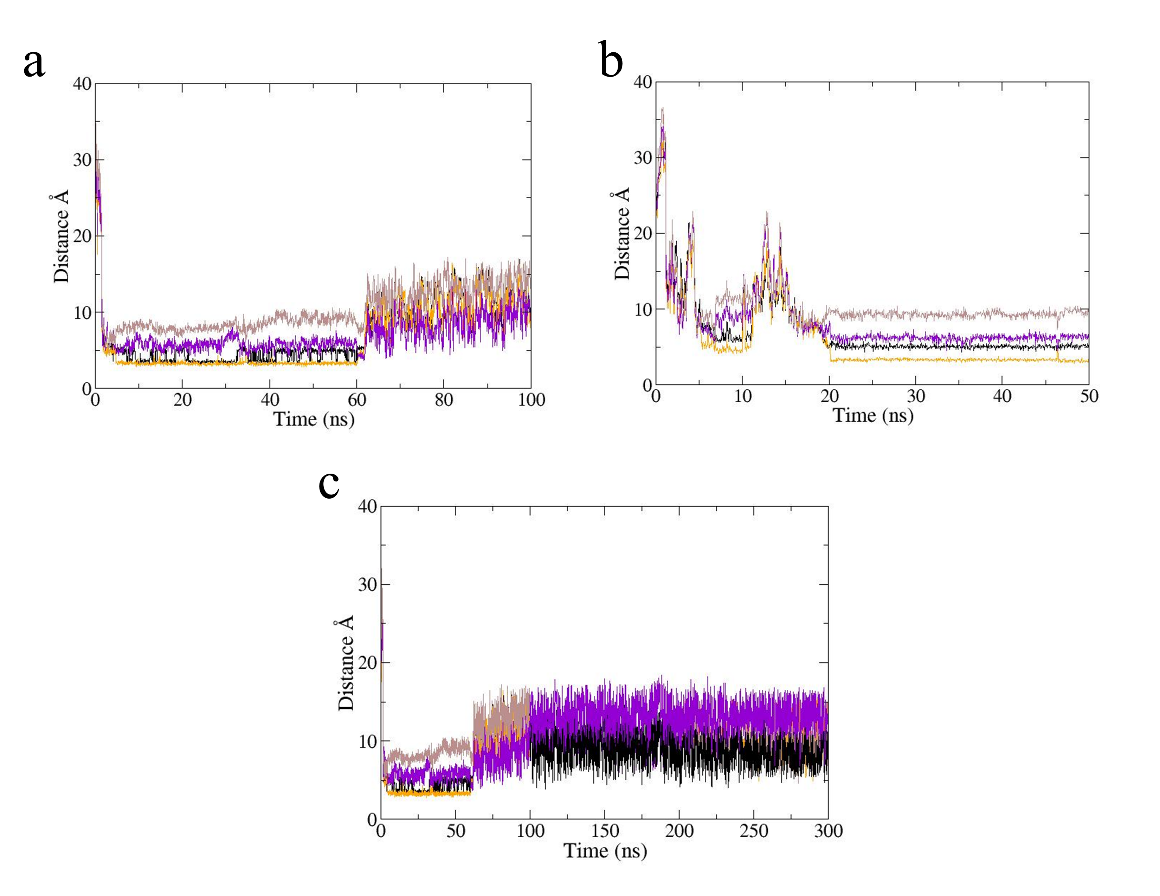


**Fig. S30** Distance plots for DEKA-selectivity filter residues of the α-subunit of the hNav1.7 sodium channel to the sodium ion. Colour code for the four lines in the chart: black Na^+^ to aspartate residue D; orange Na^+^ to glutamate E; purple Na^+^ to K; brown Na^+^ to A. a) is the measurement of the distance between the sodium ion and the DEKA selectivity filter of the first 100 ns between the α subunit complex and the subunit β3. B) is the measurement of the distance between the sodium ion and the DEKA selectivity filter for the first 50 ns in the system consisting only of α subunit. c) measurement of the distance between the sodium ion and the DEKA selectivity filter up to 300 ns, this complex is formed between the α subunit and the β3subunit.

**Part 5: Molecular dynamics DIII analysis**


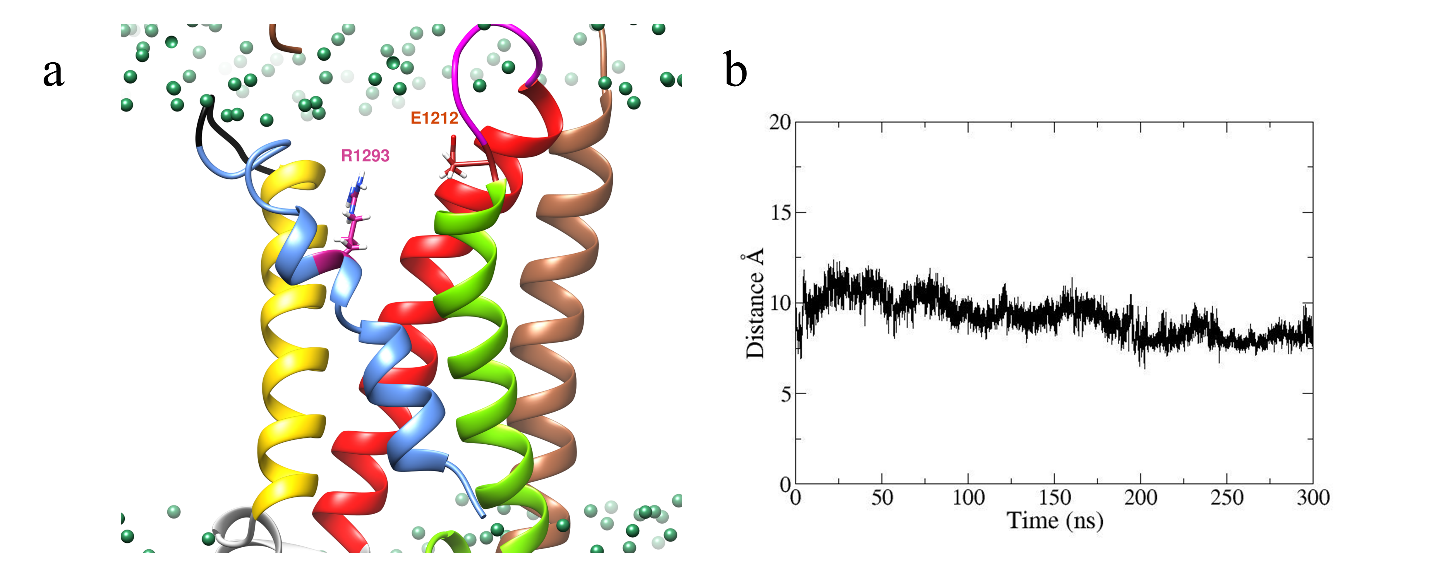


**Fig. S31** Graphic representation of the distances between R1293 and E1212. (a) This interaction between R1293–E1212 through a salt bridge at the beginning of the dynamics. Colour coding: β3 subunit in sienna, segment S1, S2, S3, S4 in chartreuse, red, yellow and cornflower blue, respectively. The phosphate group of the POPC lipid is represented by the spheres in green. (b) this interaction fluctuates because at times it stabilizes the VSD that would allow the channel in an activated state. The lower distance on y-axis is under 4 Å at on x-axis 5 ns.


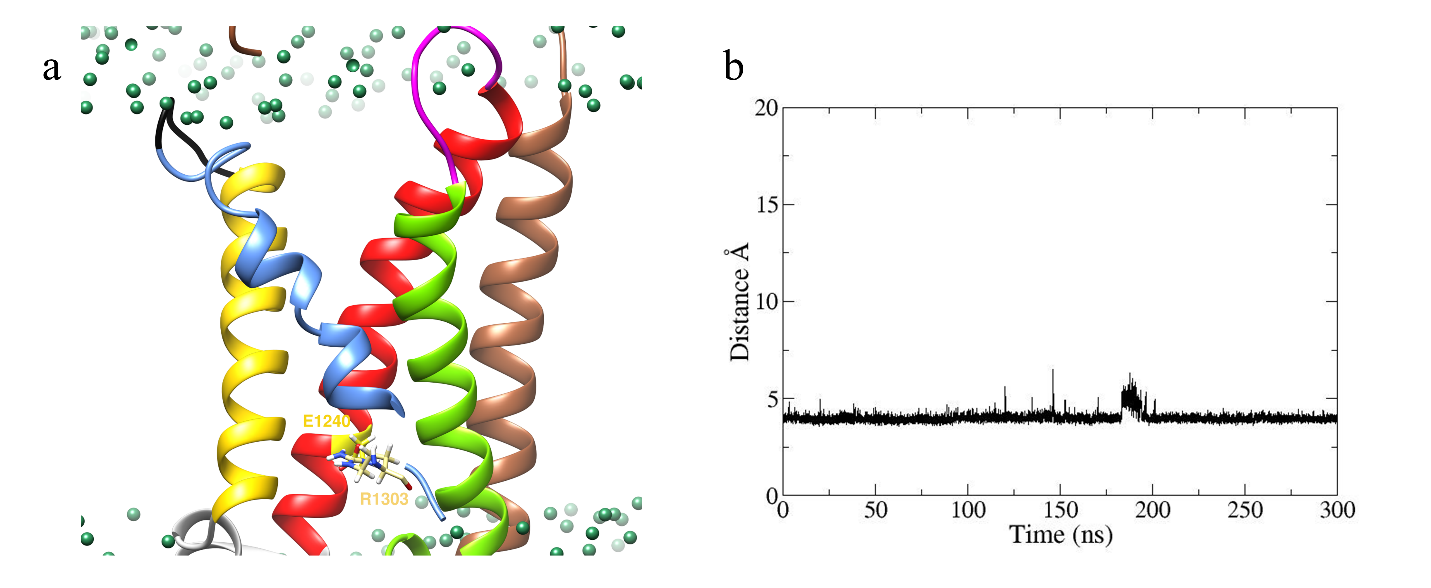


**Fig. S32** Graphic representation of the interaction between E1240 and R1303. (a) This interaction stabilizes the VSD which would allow the channel be the activated state and is not seriously affected when the S4 segment is twisted. Colour coding: β3 subunit in sienna, segment S1, S2, S3, S4 in chartreuse, red, yellow and cornflower blue, respectively. The phosphate group of the POPC lipid is represented by the spheres in green. (b) this interaction is maintained almost all the time at 4Å distance, because the S4 segment shows a bending process as the distance increases due to the movement of S4. The lower distance on y-axis is 4 Å at on x-axis 0 - 180 ns.


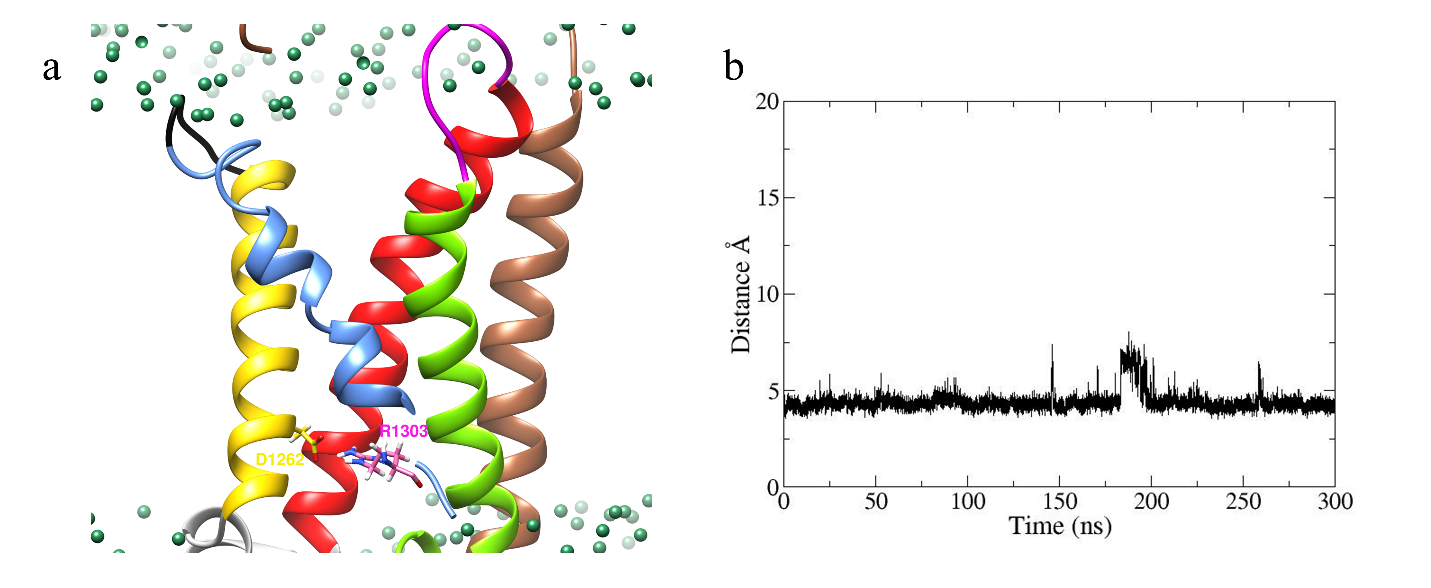


**Fig. S33** Graphic representation of the distances between D1262 and R1303. (a) This interaction stabilizes the VSD which would allow the channel be the activated state and is not seriously affected when the S4 segment is twisted. Colour coding: β3 subunit in sienna, segment S1, S2, S3, S4 in chartreuse, red, yellow and cornflower blue, respectively. The phosphate group of the POPC lipid is represented by the spheres in green. (b) The interaction distance in the formation of the salt bridge is maintained throughout the dynamics except for 180–200ns. The lower distance on y-axis is 4 Å at on x-axis 0–180 ns.


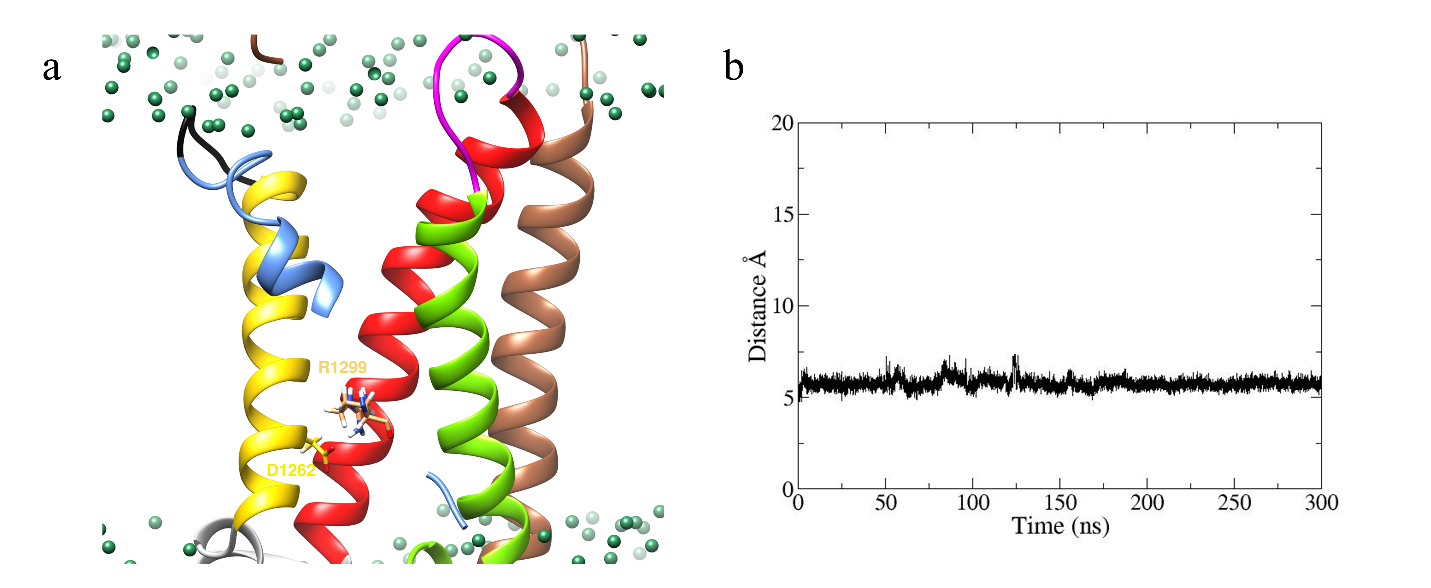


**Fig. S34** Graphic representation of the interaction between D1262–R1299 (a) The interaction is generated by forming a salt bridge. Colour coding: β3 subunit in sienna, segment S1, S2, S3, S4 in chartreuse, red, yellow and cornflower blue, respectively. The phosphate group of the POPC lipid is represented by the spheres in green (b) The formation of the salt bridge takes place during the 250 ns of molecular dynamics. The lower distance on y-axis is 4.5 Å at on x-axis 0–120 ns.


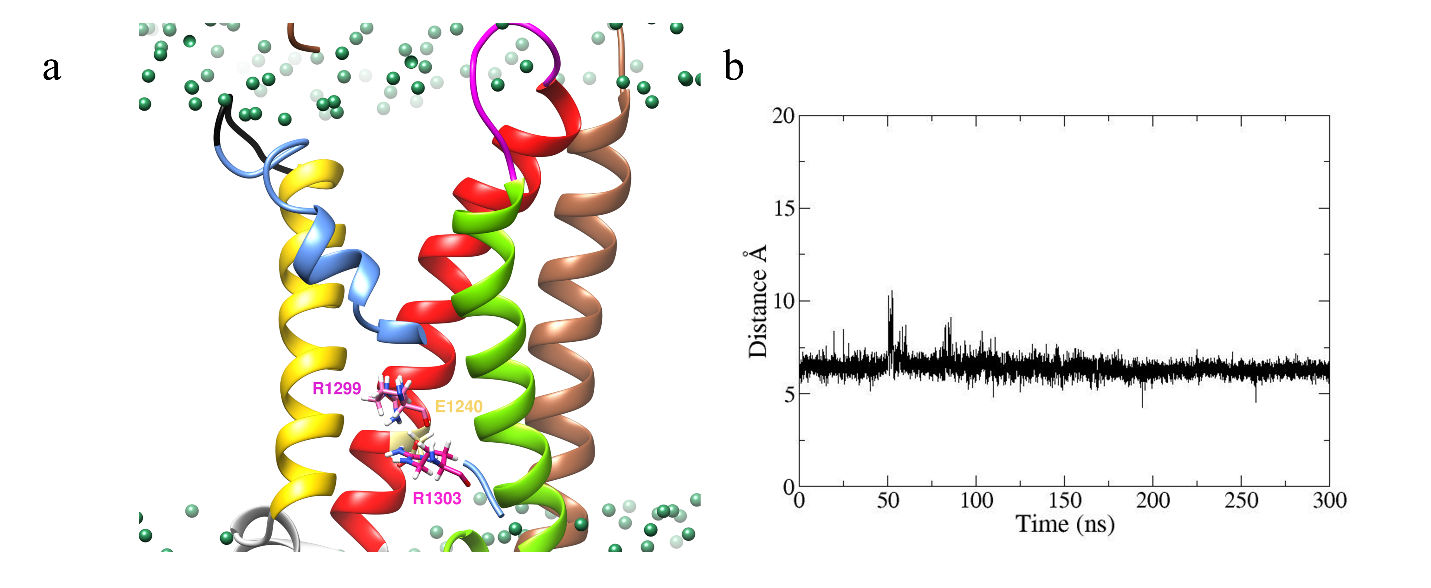


**Fig. S35** Graphic representation of the interaction between E1240–R1299. (a) This interaction is stabilized thanks to electrostatic forces, together with the repulsion caused by R1303 on R1299. Colour coding: β3 subunit in sienna, segment S1, S2, S3, S4 in chartreuse, red, yellow and cornflower blue, respectively. The phosphate group of the POPC lipid is represented by the spheres in green. (b) The distance of 6Å during the 300ns of molecular dynamics, thanks to the repulsion effect generated by R1303 and the “farness” of E1240. The lower distance on y-axis is under 6 Å at on x-axis all time.


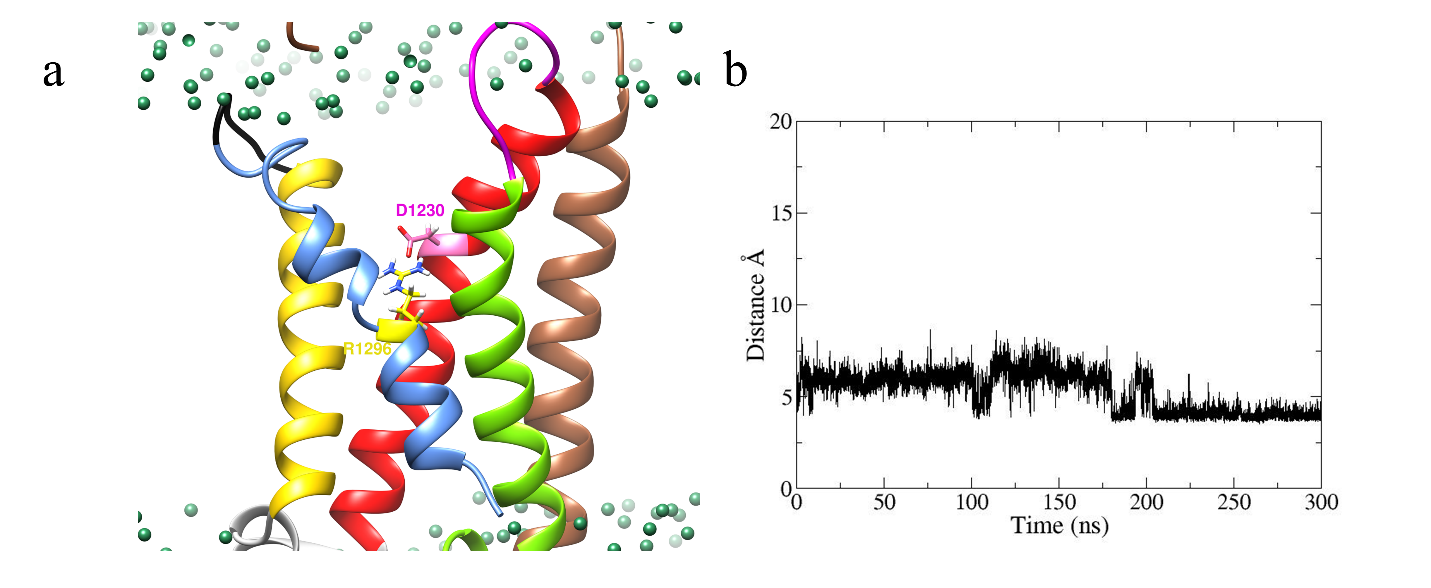


**Fig. S36** Graphic representation of the interaction between D1230–R1296. (a) The interaction is through electrostatic forces and its stabilization at 6Å distance. Colour coding: β3 subunit in sienna, segment S1, S2, S3, S4 in chartreuse, red, yellow and cornflower blue, respectively. The phosphate group of the POPC lipid is represented by the spheres in green (b) Because residue D1230 is located more towards the extracellular portion and the reduction is due to the increase in the angle of movement of R1296, thanks to the twisting process of the S4 segment. The lower distance on y-axis is under 4 Å at on x-axis 180 ns.


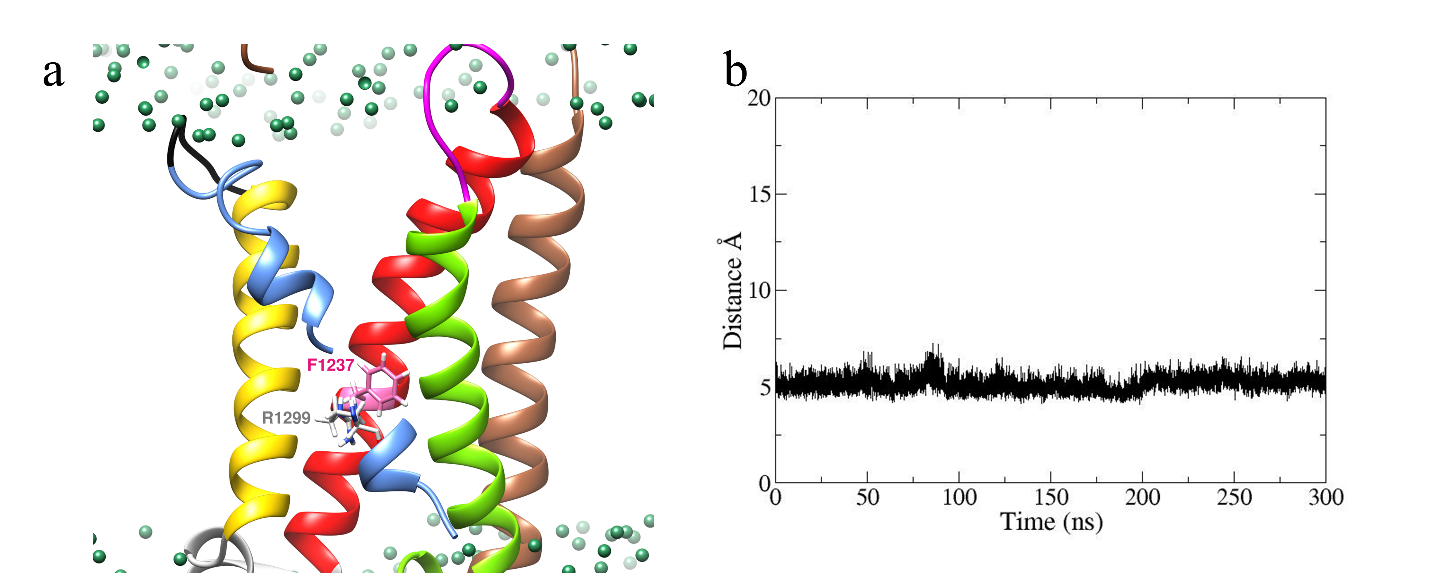


**Fig. S37** Graphic representation of the interaction between F1237–R1299. (a) This interaction allows stabilization at 5 Å distance; Because residue F1237 is located towards the central part of DIII and allows the interaction between both residues in a T-shape between the guanidinium group of R1299 and the aromatic ring of F1237. Colour coding: β3 subunit in sienna, segment S1, S2, S3, S4 in chartreuse, red, yellow and cornflower blue, respectively. The phosphate group of the POPC lipid is represented by the spheres in green. (b) The interaction distance is maintained between 4.5–5.5Å distance. The lower distance on y-axis is under 4 Å at on x-axis 200 ns.


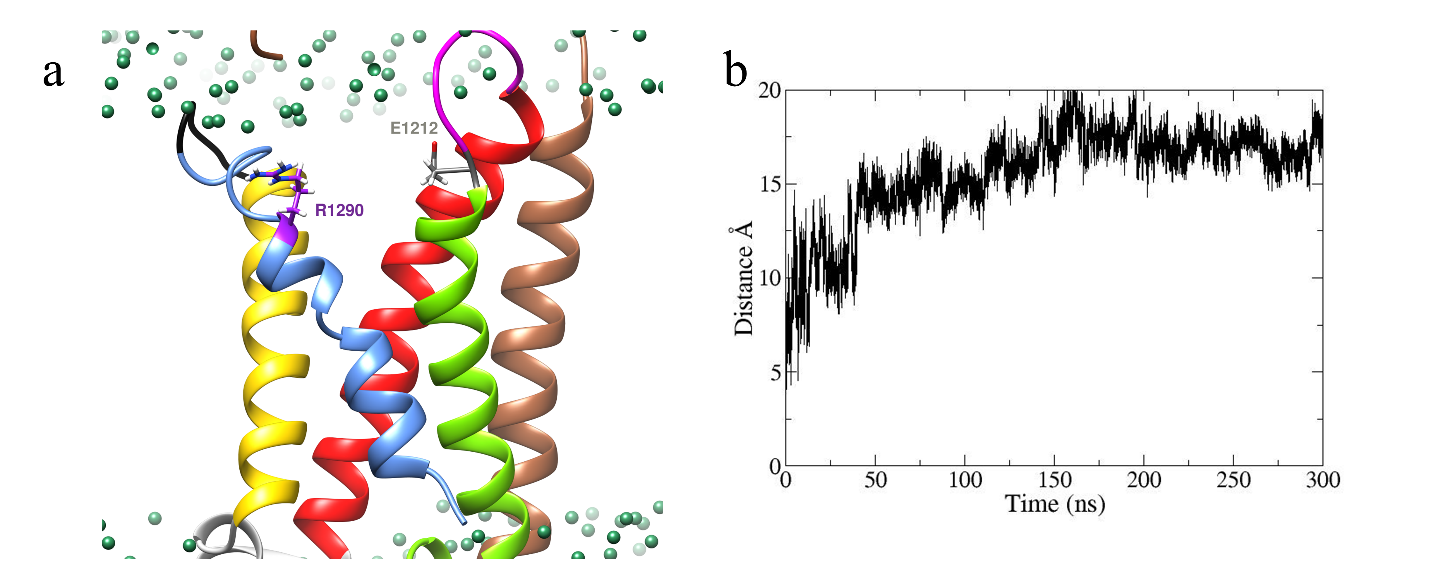


**Fig. S38** Graphic representation of the interaction between E1212–R1290. (a) This interaction presents the formation of a salt bridge at the beginning but later due to the release and interaction with the β3 subunit, this residue moves towards the extracellular part. Colour coding: β3 subunit in sienna, segment S1, S2, S3, S4 in chartreuse, red, yellow and cornflower blue, respectively. The phosphate group of the POPC lipid is represented by the spheres in green. (b) When interacting, it allows us to observe the increase in distance between both residues due to the S4 segment being in the activated state, presenting 16Å distance at 300ns. The lower distance on y-axis is under 4 Å at on x-axis 0–5 ns.


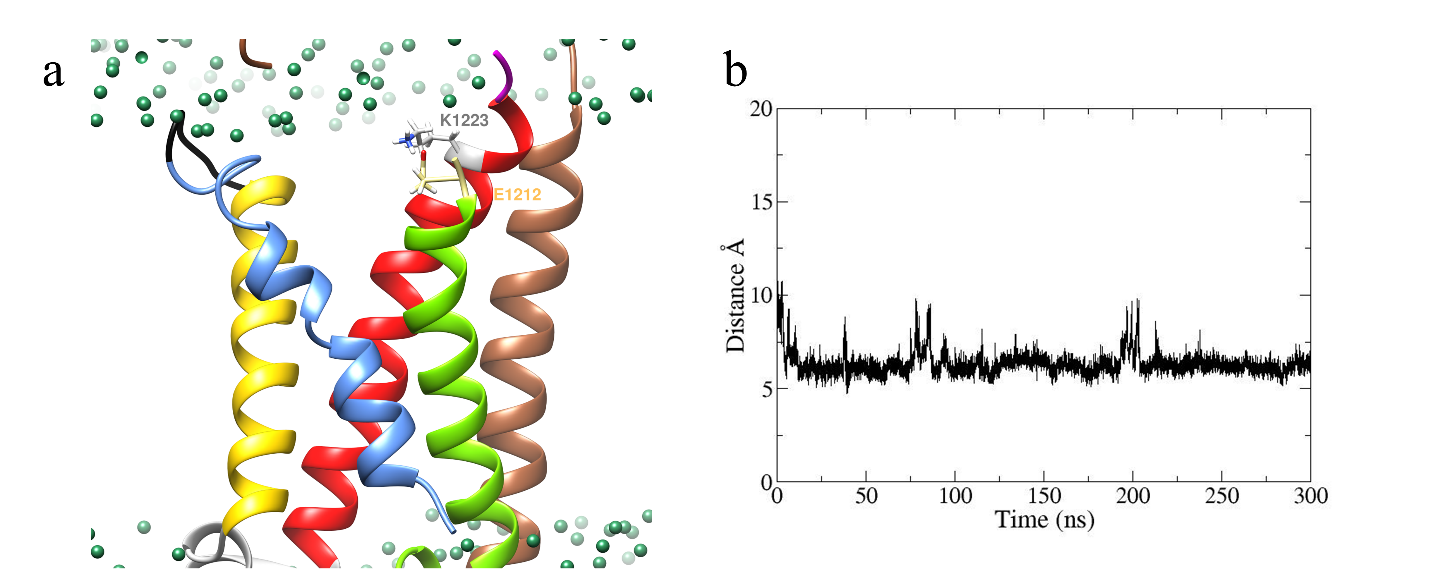


**Fig. S39** Graphic representation of the interaction between E1212–K1223. (a) This interaction presents a salt bridge allowing the stabilization of the loop between segments S1 and S2. Colour coding: β3 subunit in sienna, segment S1, S2, S3, S4 in chartreuse, red, yellow and cornflower blue, respectively. The phosphate group of the POPC lipid is represented by the spheres in green. (b) Said stabilization presents 3Å distance. The motion peaks are due to the electrostatic interaction of E1212 with R1290. The lower distance on y-axis is under 3 Å at on x-axis all time (ns).


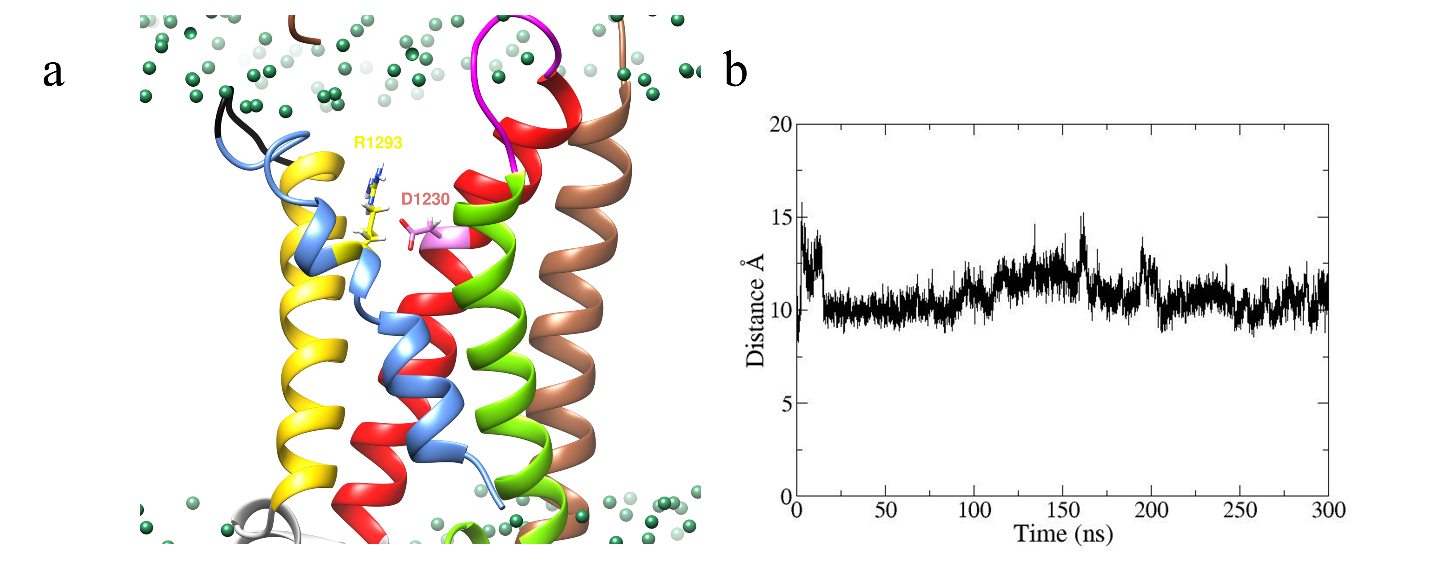


**Fig. S40** Graphic representation of the interaction between D1230–R1293. (a) This interaction allows segment S4 to stabilize in the activated position. The high movement of the R side chain of R1293 is due to the side chain being solvated and oriented towards the extracellular portion and after desolvation, this chain is oriented towards residue E1212. Colour coding: β3 subunit in sienna, segment S1, S2, S3, S4 in chartreuse, red, yellow and cornflower blue, respectively. The phosphate group of the POPC lipid is represented by the spheres in green. (b) The interaction distance is stable between 30–180 ns and after the movement process of R1290 and the solvation of this residue moves towards the extracellular part. The lower distance on y-axis is under 6 Å at on x-axis 20 ns.

**References**

1. Wang, C.; Chung, B.C.; Yan, H.; Wang, H.-G.; Lee, S.-Y.; Pitt, G.S. Structural Analyses of Ca2+/CaM Interaction with Nav Channel C-Termini Reveal Mechanisms of Calcium-Dependent Regulation. Nat. Commun. 2014, 5, 4896, https://doi.org/10.1038/ncomms5896.

2. Mahling, R.; Fowler, C.A.; Hovey, L.; Yu, L.; Gakhar, L.; Lin, Z.; Pandey, N.; Martins, T.; Shea, M.A. Structural Differences in Calmodulin Bound to Voltagate-Gated Sodium Channel IQ Motifs. Biophys. J. 2016, 110, 109a, https://doi.org/10.1016/j.bpj.2015.11.646.

3. Namadurai, S.; Balasuriya, D.; Rajappa, R.; Wiemhöfer, M.; Stott, K.; Klingauf, J.; Edwardson, J.M.; Chirgadze, D.Y.; Jackson, A.P. Crystal Structure and Molecular Imaging of the Nav Channel β3 Subunit Indicates a Trimeric Assembly. J. Biol. Chem. 2014, 289, 10797–10811, https://doi.org/10.1074/jbc.M113.527994.

4. Glass, W.G.; Duncan, A.L.; Biggin, P.C. Computational Investigation of Voltage-Gated Sodium Channel Β3 Subunit Dynamics. Front. Mol. Biosci. 2020, 7, 40, https://doi.org/10.3389/fmolb.2020.00040.

5. Yan, Z.; Zhou, Q.; Wang, L.; Wu, J.; Zhao, Y.; Huang, G.; Peng, W.; Shen, H.; Lei, J.; Yan, N. Structure of the Na v 1.4-Β1 Complex from Electric Eel. Cell 2017, 170, 470-482.e11, https://doi.org/10.1016/j.cell.2017.06.039.

6. Li, T.; Chen, J. Voltage-Gated Sodium Channels in Drug Discovery. In Ion Channels in Health and Sickness; Shad, K.F., Ed.; InTech, 2018 ISBN 978-1-78984-227-2.

7. Pan, X.; Li, Z.; Zhou, Q.; Shen, H.; Wu, K.; Huang, X.; Chen, J.; Zhang, J.; Zhu, X.; Lei, J.; et al. Structure of the Human Voltage-Gated Sodium Channel Nav1.4 in Complex with Β1. Science 2018, 362, eaau2486, https://doi.org/10.1126/science.aau2486.

8. Sanchez-Sandoval, A.L.; Hernández-Plata, E.; Gomora, J.C. Voltage-Gated Sodium Channels: From Roles and Mechanisms in the Metastatic Cell Behavior to Clinical Potential as Therapeutic Targets. Front. Pharmacol. 2023, 14, 1206136, https://doi.org/10.3389/fphar.2023.1206136.

9. Shen, H.; Liu, D.; Wu, K.; Lei, J.; Yan, N. Structures of Human Na v 1.7 Channel in Complex with Auxiliary Subunits and Animal Toxins. Science 2019, 363, 1303–1308, https://doi.org/10.1126/science.aaw2493.

10. Wang, G.; Xu, L.; Chen, H.; Liu, Y.; Pan, P.; Hou, T. Recent Advances in Computational Studies on Voltage‐gated Sodium Channels: Drug Design and Mechanism Studies. WIREs Comput. Mol. Sci. 2023, 13, e1641, https://doi.org/10.1002/wcms.1641.

11. Nguyen, P.T.; Yarov-Yarovoy, V. Towards Structure-Guided Development of Pain Therapeutics Targeting Voltage-Gated Sodium Channels. Front. Pharmacol. 2022, 13, 842032, https://doi.org/10.3389/fphar.2022.842032.

12. Salvage, S.C.; Jeevaratnam, K.; Huang, C.L. ‐H.; Jackson, A.P. Cardiac Sodium Channel Complexes and Arrhythmia: Structural and Functional Roles of the Β1 and Β3 Subunits. J. Physiol. 2023, 601, 923–940, https://doi.org/10.1113/JP283085.

13. Jiang, D.; Zhang, J.; Xia, Z. Structural Advances in Voltage-Gated Sodium Channels. Front. Pharmacol. 2022, 13, 908867, https://doi.org/10.3389/fphar.2022.908867.

14. Li, Z.; Wu, Q.; Yan, N. A Structural Atlas of Druggable Sites on Nav Channels. Channels 2024, 18, 2287832, https://doi.org/10.1080/19336950.2023.2287832.

15. Pan, X.; Li, Z.; Huang, X.; Huang, G.; Gao, S.; Shen, H.; Liu, L.; Lei, J.; Yan, N. Molecular Basis for Pore Blockade of Human Na+ Channel Nav1.2 by the μ-Conotoxin KIIIA. Science 2019, 363, 1309–1313, https://doi.org/10.1126/science.aaw2999.

16. Monastyrnaya, M.M.; Kalina, R.S.; Kozlovskaya, E.P. The Sea Anemone Neurotoxins Modulating Sodium Channels: An Insight at Structure and Functional Activity after Four Decades of Investigation. Toxins 2022, 15, 8, https://doi.org/10.3390/toxins15010008.

17. Clairfeuille, T.; Cloake, A.; Infield, D.T.; Llongueras, J.P.; Arthur, C.P.; Li, Z.R.; Jian, Y.; Martin-Eauclaire, M.-F.; Bougis, P.E.; Ciferri, C.; et al. Structural Basis of α-Scorpion Toxin Action on Nav Channels. Science 2019, 363, eaav8573, https://doi.org/10.1126/science.aav8573.

18. Noland, C.L.; Chua, H.C.; Kschonsak, M.; Heusser, S.A.; Braun, N.; Chang, T.; Tam, C.; Tang, J.; Arthur, C.P.; Ciferri, C.; et al. Structure-Guided Unlocking of Nax Reveals a Non-Selective Tetrodotoxin-Sensitive Cation Channel. Nat. Commun. 2022, 13, 1416, https://doi.org/10.1038/s41467-022-28984-4.

19. Guo, Y.; Luo, N.; Kang, X. Potential Mechanism of the Shunaoxin Pill for Preventing Cognitive Impairment in Type 2 Diabetes Mellitus. Front. Neurol. 2022, 13, 977953, https://doi.org/10.3389/fneur.2022.977953.

20. Zhang, J.; Shi, Y.; Huang, Z.; Li, Y.; Yang, B.; Gong, J.; Jiang, D. Structural Basis for Nav1.7 Inhibition by Pore Blockers. Nat. Struct. Mol. Biol. 2022, 29, 1208–1216, https://doi.org/10.1038/s41594-022-00860-1.

21. Li, X.; Xu, F.; Xu, H.; Zhang, S.; Gao, Y.; Zhang, H.; Dong, Y.; Zheng, Y.; Yang, B.; Sun, J.; et al. Structural Basis for Modulation of Human Nav1.3 by Clinical Drug and Selective Antagonist. Nat. Commun. 2022, 13, 1286, https://doi.org/10.1038/s41467-022-28808-5.

22. Zidar, N.; Tomašič, T.; Kikelj, D.; Durcik, M.; Tytgat, J.; Peigneur, S.; Rogers, M.; Haworth, A.; Kirby, R.W. New Aryl and Acylsulfonamides as State-Dependent Inhibitors of Nav1.3 Voltage-Gated Sodium Channel. Eur. J. Med. Chem. 2023, 258, 115530, https://doi.org/10.1016/j.ejmech.2023.115530.

23. Huang, G.; Liu, D.; Wang, W.; Wu, Q.; Chen, J.; Pan, X.; Shen, H.; Yan, N. High-Resolution Structures of Human Nav1.7 Reveal Gating Modulation through α-π Helical Transition of S6IV. Cell Reports 2022, 39, 110735, https://doi.org/10.1016/j.celrep.2022.110735.

24. Huang, X.; Jin, X.; Huang, G.; Huang, J.; Wu, T.; Li, Z.; Chen, J.; Kong, F.; Pan, X.; Yan, N. Structural Basis for High-Voltage Activation and Subtype-Specific Inhibition of Human Na v 1.8. Proc. Natl. Acad. Sci. U.S.A. 2022, 119, e2208211119, https://doi.org/10.1073/pnas.2208211119.

25. Raffo, A.; Fugacci, U.; Biasotti, S. GEO-Nav: A Geometric Dataset of Voltage-Gated Sodium Channels. Comput. Graph. 2023, 115, 285–295, https://doi.org/10.1016/j.cag.2023.06.023.

26. Huang, G.; Wu, Q.; Li, Z.; Jin, X.; Huang, X.; Wu, T.; Pan, X.; Yan, N. Unwinding and Spiral Sliding of S4 and Domain Rotation of VSD during the Electromechanical Coupling in Na v 1.7. Proc. Natl. Acad. Sci. U.S.A. 2022, 119, e2209164119, https://doi.org/10.1073/pnas.2209164119.

27. Liu, Y.; Bassetto, C.A.Z.; Pinto, B.I.; Bezanilla, F. A Mechanistic Reinterpretation of Fast Inactivation in Voltage-Gated Na+ Channels. Nat. Commun. 2023, 14, 5072, https://doi.org/10.1038/s41467-023-40514-4.

28. Huang, J.; Fan, X.; Jin, X.; Teng, L.; Yan, N. Dual-Pocket Inhibition of Na v Channels by the Antiepileptic Drug Lamotrigine. Proc. Natl. Acad. Sci. U.S.A. 2023, 120, e2309773120, https://doi.org/10.1073/pnas.2309773120.

29. Li, Y.; Yuan, T.; Huang, B.; Zhou, F.; Peng, C.; Li, X.; Qiu, Y.; Yang, B.; Zhao, Y.; Huang, Z.; et al. Structure of Human Nav1.6 Channel Reveals Na+ Selectivity and Pore Blockade by 4,9-Anhydro-Tetrodotoxin. Nat. Commun. 2023, 14, 1030, https://doi.org/10.1038/s41467-023-36766-9.

30. Wu, Q.; Huang, J.; Fan, X.; Wang, K.; Jin, X.; Huang, G.; Li, J.; Pan, X.; Yan, N. Structural Mapping of Nav1.7 Antagonists. Nat. Commun. 2023, 14, 3224, https://doi.org/10.1038/s41467-023-38942-3.

31. Studer, G.; Biasini, M.; Schwede, T. Assessing the local structural quality of transmembrane protein models using statistical potentials (QMEANBrane). Bioinformatics 2014, 30, i505-i511, https://doi.org/10.1093/bioinformatics/btu457.

32. Benkert, P.; Biasini, M.; Schwede, T. Toward the estimation of the absolute quality of individual protein structure models. Bioinformatics 2011, 27, 343-350, https://doi.org/10.1093/bioinformatics/btq662.

33. Johansson, M.U.; Zoete V.; Michielin O. & Guex N. Defining and searching for structural motifs using DeepView/Swiss-PdbViewer. BMC Bioinformatics 2012, 13, 173 https://doi.org/10.1186/1471-2105-13-173.

34. Guex, N and Peitsch, M.C. Swiss-PdbViewer: A Fast and Easy-to-use PDB Viewer for Macintosh and PC. Protein Data Bank Quaterly Newsletter 1996, 77, 7. https://doi.org/10.1002/elps.1150181505.

35. Zhu, W.; Voelker, T.L.; Varga, Z.; Schubert, A.R.; Nerbonne, J.M.; Silva, J.R. Mechanisms of Noncovalent β Subunit Regulation of Nav Channel Gating. J. Gen. Physiol. 2017, 149, 813–831, https://doi.org/10.1085/jgp.201711802.

36. Barro-Soria, R.; Liin, S.I.; Larsson, H.P. Using Fluorescence to Understand β Subunit–Nav Channel Interactions. J. Gen. Physiol. 2017, 149, 757–762, https://doi.org/10.1085/jgp.201711843.

37. Yan Y, Zhang D, Zhou P, Li B, Huang S-Y. HDOCK: a web server for protein-protein and protein-DNA/RNA docking based on a hybrid strategy. Nucleic Acids Res. 2017, 45(W1):W365-W373, https://doi.org/10.1093/nar/gkx407

38. Yan Y, Wen Z, Wang X, Huang S-Y. Addressing recent docking challenges: A hybrid strategy to integrate template-based and free protein-protein docking. Proteins 2017, 85:497-512, https://doi.org/10.1002/prot.25234

39. Honorato, R.V.; Trellet, M.E.; Jiménez-García, B.; Schaarschmidt, J.J.; Giulini, M.; Reys, V.; Koukos, P.I.; Rodrigues, J.P.G.L.M.; Karaca, E.; van Zundert, G.C.P.; Roel-Touris, J.; van Noort, C.W.; Jandová, Z.; Melquiond, A.S.J. and Bonvin, A.M.J.J. The HADDOCK2.4 web server: A leap forward in integrative modelling of biomolecular complexes. Nature Prot., In Press. 2024, https://doi.org/10.1038/s41596-024-01011-0.

40. Honorato, R.V.; Koukos, P.I.; Jimenez-Garcia, B.; Tsaregorodtsev, A.; Verlato, M.; Giachetti, A.; Rosato, A. and Bonvin, A.M.J.J. Structural biology in the clouds: The WeNMR-EOSC Ecosystem. Frontiers Mol. Biosci. 2021, 8, https://doi.org/fmolb.2021.729513
